# Diverse priming outcomes under conditions of very rare precursor B cells

**DOI:** 10.1016/j.immuni.2025.03.003

**Published:** 2025-03-31

**Authors:** Patrick J. Madden, Ester Marina-Zárate, Kristen A. Rodrigues, Jon M. Steichen, Monolina Shil, Kaiyuan Ni, Katarzyna Kaczmarek Michaels, Laura Maiorino, Amit A. Upadhyay, Swati Saha, Arpan Pradhan, Oleksandr Kalyuzhiny, Alessia Liguori, Paul G. Lopez, Ivy Phung, Claudia Flynn, Amelia Zhou, Mariane B. Melo, Ashley Lemnios, Nicole Phelps, Erik Georgeson, Nushin Alavi, Michael Kubitz, Danny Lu, Saman Eskandarzadeh, Amanda Metz, Oscar L. Rodriguez, Kaitlyn Shields, Steven Schultze, Melissa L. Smith, Brandon S. Healy, Deuk Lim, Vanessa R. Lewis, Elana Ben-Akiva, William Pinney, Justin Gregory, Shuhao Xiao, Diane G. Carnathan, Sudhir Pai Kasturi, Corey T. Watson, Steven E. Bosinger, Guido Silvestri, William R. Schief, Darrell J Irvine, Shane Crotty

**Affiliations:** 1 La Jolla Institute for Immunology, La Jolla, CA, USA; 2 Consortium for HIV/AIDS Vaccine Development (CHAVD), The Scripps Research Institute, La Jolla, CA, USA; 3 Koch Institute for Integrative Cancer Research, Massachusetts Institute of Technology, Cambridge, MA, USA; 4 Department of Immunology and Microbiology, The Scripps Research Institute, La Jolla, CA, USA; 5 Emory National Primate Research Center and Emory Vaccine Center, Emory University School of Medicine, Atlanta, GA, USA; 6 Department of Pathology and Laboratory Medicine, Emory School of Medicine, Atlanta, GA, USA; 7 Department of Biochemistry and Molecular Genetics, University of Louisville School of Medicine, Louisville, KY, USA; 8 IAVI Neutralizing Antibody Center, The Scripps Research Institute, La Jolla, CA, USA; 9 Moderna, Inc., Cambridge, MA, USA; 10 Department of Biological Engineering, Massachusetts Institute of Technology, Cambridge MA USA; 11 Howard Hughes Medical Institute, Chevy Chase, MD, USA; 12 Department of Medicine, Division of Infectious Diseases and Global Public Health, University of California, San Diego, La Jolla, CA, USA; 13 These authors contributed equally; 14 Lead contact

## Abstract

Rare naive B cells have special pathogen-recognition features that enable outsized contributions to protective immunity, but infrequently participate in immune responses. We investigated how germline-targeting vaccine delivery and adjuvant selection affect priming of exceptionally rare BG18-like HIV broadly neutralizing antibody-precursor B cells (<1-in-50 million) in non-human primates. Only escalating dose (ED) priming immunization using the saponin adjuvant SMNP elicited detectable BG18-like cells in germinal centers (GCs) compared to other conditions. All groups had strong GC responses, but only ED+SMNP and bolus+SMNP induced BG18-like memory B cells in >50% of animals. One group had vaccine-specific GC responses equivalent to ED+SMNP, but scarce BG18-like B cells. Following homologous boosting, BG18-like memory B cells were present in a bolus priming group, but with lower somatic hypermutation and affinities than ED+SMNP. This outcome inversely associated with post-prime antibody titers, suggesting antibody feedback significantly influences rare precursor B cell responses. Thus, antigen and inflammatory stimuli extensively impact priming and affinity maturation of rare B cells.

## eTOC

B cells that give rise to neutralizing antibody responses can be extremely rare in the naive B cell repertoire. Madden et al. show that delivery strategy and adjuvant selection affect the recruitment and expansion of rare broadly neutralizing antibody precursor B cells after immunization with an HIV germline-targeting vaccine and that antibody feedback appears to substantially influence the boosting response.

## Graphical Abstract

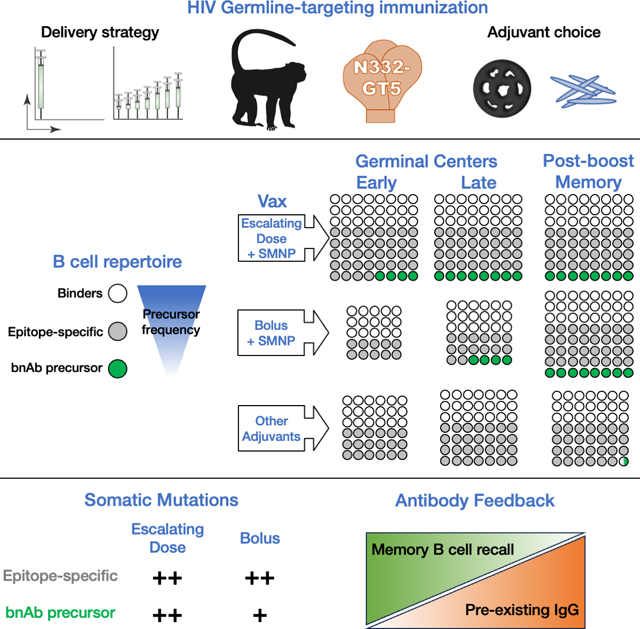

## INTRODUCTION

The aim of many vaccines against viruses is to induce neutralizing antibodies that protect against infection^1–3^. B cell precursors that give rise to these types of neutralizing responses can be extremely rare in the naive B cell repertoire and infrequently participate in immune responses^4–12^. Germline-targeting vaccine design is one approach to prime these rare B cell specificities by designing immunogens that have affinity for the precursors of choice^13–16^. HIV broadly neutralizing antibody (bnAb) precursors have been the focus of germline-targeting immunogen design^5,8,12,17–28^. This approach relies on the concept that immunogens that can be recognized with high affinity by bnAb-precursor B cells can overcome their inherent rarity and allow for sufficient B cell activation and recruitment into germinal center (GC) reactions^12,19,29^. Using adoptive transfer experiments in mice to model human precursor frequencies, rare bnAb-precursor B cells can be successfully primed if the immunogen has sufficient affinity for the target naive B cell receptors (BCRs)^4^. These outcomes have been tested and validated in a first-in-humans germline-targeting vaccine clinical trial wherein rare VRC01-class bnAb-precursors were primed in 97% of vaccine recipients^26^.

It was recently shown that a novel HIV germline-targeting immunogen, N322-GT5, designed to target and prime rare precursors similar to the HIV bnAb BG18, successfully primed BG18-class responses in 8-of-8 rhesus macaques (RMs)^12,19,25^. In humans, N332-GT5 reactive BG18-class precursors were found at a frequency of 1-in-53 million in the naive B cell repertoire^12^, and are estimated to be 8-fold rarer in RM^25^. In addition to being an important advance in HIV candidate vaccine development in the germline-targeting vaccine field, the success of N332-GT5 in NHPs provides opportunities for a model system to study rare B cell priming (defined as precursor frequencies <1-in-1 million naive B cells) in outbred, non-transgenic animals. It is particularly advantageous to be able to do so in a primate species, given the much more similar immunoglobulin gene repertoire features and heavy chain complementarity determining region 3 (H-CDR3) lengths between non-human primates and humans compared to rodents^30,31^.

It is yet to be elucidated how adjuvants and antigen delivery strategies affect recruitment of rare B cells into GCs, despite much activity in adjuvant development and new vaccine technologies^32–36^. To build on the success of germline targeting immunogen design and explore the immunology of rare B cell recruitment, here we investigated the impact of various delivery strategies and adjuvants on priming BG18-class precursor B cells in RMs. The results reveal key features about successful versus failed priming and maturation of rare naive B cells in different contexts.

## RESULTS

### Adjuvant/delivery strategies and vaccine study design

**Figure 1:**
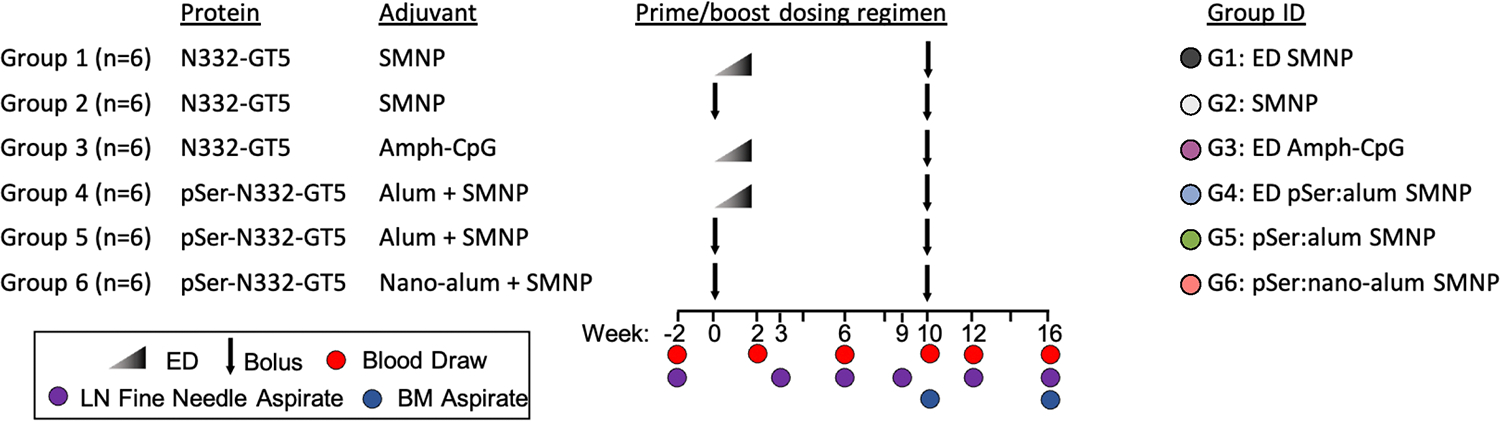
Study schematic showing immunization groups and sampling timepoints

To assess whether different immunization strategies affect recruitment of rare bnAb-precursor B cells after a germline-targeting priming immunization, 36 RMs were split into six groups (n = 6/group) and immunized with the recombinant HIV Env-based germline-targeting immunogen N332-GT5 (Figure 1). ED immunization is a slow antigen delivery technique^37,38^. Saponin and monophosphoryl lipid A (MPLA)-containing nanoparticle (SMNP) is a potent adjuvant^25,39^ being developed for clinical use^40^. ED immunization adjuvanted with other immune stimulating complexes (ISCOMs) has been previously shown to recruit a more diverse set of naive B cells to participate in GCs^38^, including rare neutralizing antibody-precursor B cells, when compared to traditional bolus immunization^37^. ED immunization adjuvanted with SMNP (ED+SMNP) successfully primed BG18-class B cells in NHPs^25^.

We hypothesized that the extended-antigen availability afforded by ED immunization may lead to better recruitment and priming of BG18-class B cells^25,41^. An additional method to extend antigen availability is via adsorption of phosphoserine-tagged immunogens on aluminum hydroxide (alum) (pSer:alum)^42^. Tethering immunogens to alum particles slows antigen clearance while simultaneously increasing the potential avidity of the immunogen^42–44^. Previously, we have shown that pSer:alum delivery of a native-like HIV Env trimer in RMs enhanced responses compared to unmodified antigen with alum, and further adjuvanting with SMNP led to even better responses^45^. BG18 targets the V3/glycan site on HIV Env, which includes the N322 glycan. By orienting the N332-GT5 Env trimer on alum, it may promote responses to the V3/glycan bnAb epitope and reduce off-target Env base-binding responses^42,43,45^. We thus synthesized N332-GT5 trimers with an optimized C-terminal Cys linker^43^, and coupled a peptide containing 4 phosphoserines to the base of each protomer to enable pSer-mediated anchoring to alum (Figure S1A). pSer-modified N332-GT5 showed stable binding to alum in the presence of serum and exhibited high binding of HIV bnAbs (particularly inferred-germline BG18) and low binding of trimer base-specific Abs (Figure S1B–D).

Alum is a suspension of aluminum hydroxide nanocrystals that aggregate to form particles of varying sizes^42,46,47^. Stable dispersions of sub-micron sized aluminum hydroxide particles, termed nano-alum, have been shown to elicit stronger antibody responses when compared to normal alum^48^. To test nano-alum in the setting of germline targeting, we generated nano-alum by dispersing Alhydrogel in the presence of a poly(ethylene glycol) (PEG) phospholipid. The PEG-lipid bound to the alum through its phosphate group, allowing individual needle-like aluminum hydroxide crystals to be stably dispersed in buffer (Figure S2A). Nano-alum had a particle size under 100 nm diameter (Figure S2B–C) and formed a stable opalescent solution in water (Figure S2D). When mixed with pSer-trimer, Env trimers could be observed decorating the length of individual nano-alum crystals by TEM (Figure S2E). In mice, nano-alum loaded with pSer-modified Env trimers elicited substantial increases in antigen-specific GC B cells (B_GC,_) and serum IgG titers compared to traditional alum (Figure S2F–H).

Toll-like receptor (TLR) agonists are also being widely studied as vaccine adjuvants^33,34,36,39^, and this study evaluated multiple TLR agonist formulations. The MPLA in SMNP is a TLR-4 agonist. CpG is a TLR9 agonist that can be further enhanced by the addition of an albumin-binding lipid tail (Amph-CpG, Figure S3)^49^. Amph-CpG takes advantage of the fact that albumin cannot efficiently pass from tissue to blood and therefore must enter lymphatics^49 50^.

Six study groups comprised different combinations of three delivery strategies (bolus, ED, and pSer) and four adjuvants (SMNP, alum, nano-alum, Amph-CpG) and are detailed in Figure 1. All immunizations were administered subcutaneously in each thigh (50μg protein/limb, 100μg total) at weeks 0 and 10 (Figure 1A). The ED regimen consists of 7 immunizations across 12 days with an exponentially increasing dose of antigen and adjuvant to total 50μg protein/limb. The week 10 boosting immunizations were bolus, using the same immunogen and adjuvant combination used at the priming timepoint. To date, only ED+SMNP delivery of N332-GT5 has been tested and shown to prime BG18 type I precursor cells. A primary objective of this study was to thus determine the immunology of rare bnAb-precursor B cell priming under a range of immunization conditions. Two secondary objectives were also explored to determine whether the pSer:alum based immunizations were superior to traditional bolus immunization, and to assess how pSer:alum behaved when delivered in an ED immunization with different adjuvants. Statistical tests presented here were designed to reflect these specific objectives (see Methods).

### ED immunizations and SMNP prime larger GC responses

**Figure 2:**
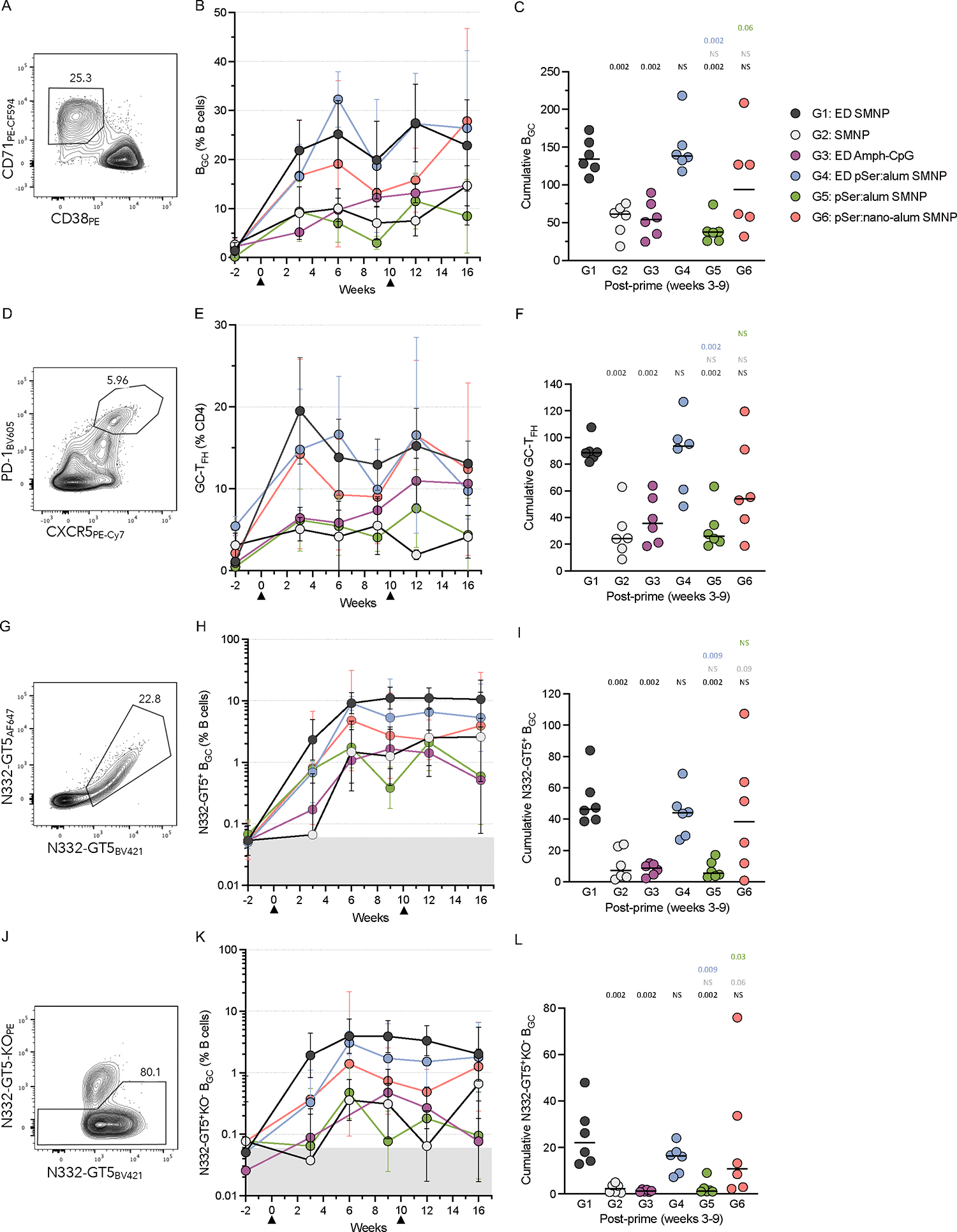
ED+SMNP prime larger GC responses. **A)** Flow cytometry gating of B_GC_ cells (CD38^−^CD71^+^), **B)** frequency of total B cells (CD20^+^), and **C)** cumulative frequencies post-prime (weeks 3–9) based on area-under-the-curve (AUC) of B. Full gating shown in Figure S4A. **D)** Flow cytometry gating of GC-T_FH_ cells (PD-1^Hi^CXCR5^+^), **E)** frequency of total CD4^+^ cells, and **F)** cumulative frequencies post-prime (AUC of E). **G)** Flow cytometry gating of antigen-specific B_GC_ cells (N332-GT5-AF647^+^N332-GT5-BV421^+^;N332-GT5^++^), **H)** frequency of total B cells, and **I)** cumulative frequencies post-prime (AUC of H). **J)** Flow cytometry gating of epitope-specific B_GC_ cells (N332-GT5-AF647^+^N332-GT5-BV421^+^N332-GT5KO-PE^−^), **K)** frequency of total B cells, and **I)** cumulative frequencies post-prime (AUC of K). Triangles (B,E,H,K) represent immunizations. Mean and SEM (B,E) or geometric mean and SD (H,K) are plotted in longitudinal figures. Median is plotted in all per animal figures. Statistical significance was tested using unpaired two-tailed Mann-Whitney tests as described in Methods, NS: p>0.1. Gray regions (H,K) represent B_GC_ LOD. See also Figure S4.

GC responses were measured by flow cytometry from lymph node fine needle aspirates (LN FNAs) at multiple timepoints (Figure S4A). In addition, cumulative post-prime GC responses were quantified by integrating data from weeks 3–9. ED immunizations led to significantly larger total B_GC_ (CD38^−^CD71^+^) and GC-T_FH_ (PD-1^Hi^CXCR5^+^) responses post-prime when compared to bolus groups using the same adjuvant combinations (G1 vs G2, and G4 vs G5, Figure 2A–F). G3 had lower B_GC_ and GC-T_FH_ responses throughout the study compared to G1 and G4, indicating that SMNP was more effective in driving GC responses than Amph-CpG in the setting of escalating-dose immunization (Figure 2B, E).

B_GC_ cells were stained with fluorophore-labeled N332-GT5 probes to detect antigen-specific (two colors of N332-GT5 probes; N332-GT5^++^) and epitope-specific (N332-GT5^++^N332-GT5KO^−^) B_GC_ cells (Figure 2G–L). All groups had detectable antigen- and epitope-specific B_GC_ cells starting at week 3, but the kinetics of GC priming differed (Figure 2G–L). G1 and G4 had the largest post-prime antigen- and epitope-specific B_GC_ frequencies. At week 3, bolus+SMNP vaccination (G2) elicited a frequency of antigen-specific B_GC_ cells barely above the limit of detection (LOD, baseline determined pre-immunization), followed by a sharp rise at week 6, which was mirrored by the expansion of epitope-specific B_GC_ cells (Figure 2H, K). Interestingly, all of the groups employing SMNP in an extended-delivery regimen (either ED or pSer) expanded antigen-specific B_GC_ cells 10-fold or more by week 3 (Figure 2H). This was most pronounced in G1, where by week 3 both antigen- and epitope-specific B_GC_ cells were rapidly increased 30–40-fold above baseline. Despite the rise at week 6, G2 still had a ~10-fold lower frequency of epitope-specific B_GC_ cells at weeks 6 and 9 compared to G1 (Figure 2K, p = 0.002 week 6 and 0.002 week 9, Mann-Whitney). When looking at cumulative post-prime responses, G1 had significantly higher values for both antigen- and epitope-specific B_GC_ cells compared to all groups except G4 and G6 (Figure 2I, L). These data suggest that ED+SMNP generated robust GCs faster than other groups and recruited epitope-specific cells to GCs to a greater extent than all but G4 and G6.

Antigen- and epitope-specific non-B_GC_ cells were examined at weeks 3 and 9 to determine if certain groups drove a more extrafollicular or memory response in lymph nodes. The hierarchy of the non-B_GC_ cell responses for the animal groups mirrored that seen for the B_GC_ for both antigen- and epitope-specific cells (Figure S4B–C).

Unexpectedly, the slow-delivery behavior of pSer:alum combined with SMNP immunization did not enhance total GC or antigen-specific B_GC_ cell responses over SMNP alone in either bolus or ED administration. However, pSer-conjugated trimer delivered on nano-alum led to significantly more epitope-specific B_GC_ cells out to week 10 compared to traditional alum (G6 vs G5) and was the only bolus-administered formulation to approach ED dosing in terms of GC response magnitude (Figure 2I, L), suggesting that pSer:nano-alum has a unique mechanism of action.

Overall, these longitudinal data indicate that the two ED+SMNP-containing immunizations primed the most robust total and antigen-specific GC responses, with notably accelerated B_GC_ expansion compared to bolus vaccination. Enhanced frequencies of epitope-specific B_GC_ suggested that ED immunization with SMNP can better recruit diverse B cells after priming compared to bolus or non-SMNP adjuvanted immunizations.

### ED+SMNP leads to a more diverse BCR response

**Figure 3:**
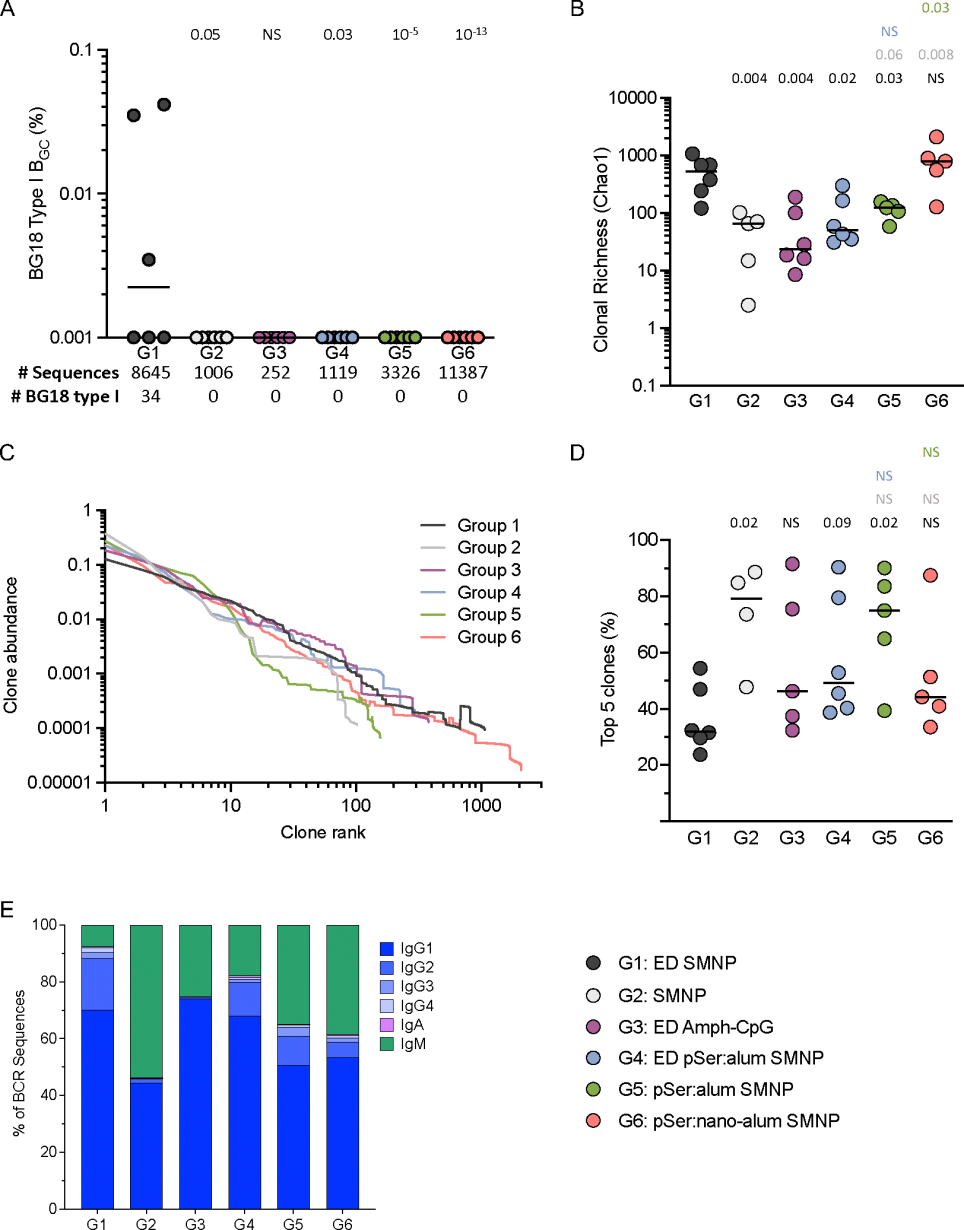
ED+SMNP leads to a larger, more diverse, composition of BCRs 3-weeks post-prime. **A)** Frequency of BG18 type I B_GC_ from week 3 among total B cells. Table indicates total and number of BG18 type I. Definitions shown in Figure S5A. **B)** Clonal richness of the B_GC_ BCRs plotted as Chao1 index. **C)** Clonal abundance curves for each group, and **D)** cumulative abundance of the top-5 clones per animal. **E)** Ig isotype distribution from each group. (A,B,D) lines represent medians. Statistical significance was tested using unpaired two-tailed Mann-Whitney tests as described in Methods. In A) multiple Fisher’s exact tests were used to compare each group to G1, NS: p>0.1. See also Figure S5.

Epitope-specific B_GC_ cells were sorted and single cell BCR sequencing was completed at week 3 post-immunization to assess the ability of different delivery and adjuvant combinations to recruit and prime rare BG18 type I precursors. A total of 25,735 epitope-specific paired BCRs from week 3 LN FNAs were sequenced across all groups. BG18 type I BCRs are defined based on immunoglobulin heavy chain (HC) sequence features and H-CDR3 length (Figure S5A). BG18 type I BCRs were detected in 3/6 animals from G1 but none in any other group (Figure 3A; Table S1). The results from G1 were consistent with the original N332-GT5 RM study, where 5/8 ED+SMNP animals had BG18 type I B_GC_ cells at week 3 (ref. ^25^). G4 and G6 had similar total post-prime antigen- and epitope-specific B_GC_ cells compared to G1 (Figure 2H, K), thus it was surprising that no BG18 type I BCRs were found in these groups. If precursor priming was equivalent, BG18 type I BCRs were expected in all groups except the non-SMNP group (G3) based on total sequence number (Figure 3A). These data indicate that only ED+SMNP immunization possessed properties necessary for successful recruitment and expansion of rare BG18 type I precursor cells above a limit of detection of ~1-in-3,000 B_GC_ by 3-weeks post-vaccination.

As an additional measure of rare B cells, we searched for two other populations: cells similar to BG18 type I cells but with shorter H-CDR3 length, and potential BG18 type III cells. The standard definition used for BG18 type I cells in RMs includes H-CDR3s of at least 22 amino acids (AA)^12^, but we observed BCRs that otherwise met the criteria for being BG18 type I but with 20–21 AA H-CDR3s (“BG18_20–21AA_” Figure S5A–B). BG18_20–21AA_ cells were found in the same three animals from G1 where the traditional BG18 type I cells were found (Figure S5B). We also observed a single BG18_20–21AA_ BCR in one animal each from G2 and G3 despite no traditional BG18s being found. BG18 type III BCRs, defined as those having H-CDR3 lengths ≥20 AAs and a binding angle of approach and footprint similar to BG18 type I but lacking other sequence features of BG18 type I or type II, were previously identified from RMs immunized with N332-GT5 ^25^. Potential type III cells were found herein at similar frequencies to the traditional and BG18_20–21AA_ responses and were identified in two additional animals (Figure S5C). These results further corroborate that rare potential bnAb-precursor B cells are recruited and expanded after ED+SMNP immunization better than other immunization strategies.

To further understand the composition of the B_GC_ response at the population level, clonal richness (Chao1) and diversity (Simpson index) were calculated for epitope-specific B_GC_. ED+SMNP elicited a larger and more diverse population of B_GC_ cells compared to all others groups except G6 (Figure 3B; S5D). There were no significant differences between pSer:alum groups G2, G4, and G5 in either clonal richness or diversity, indicating that pSer:alum delivery, by bolus or ED, offered no clear advantage over bolus immunization for increasing on-target B cell diversity (Figure 3B; S5D). In contrast, G6 had clonal richness comparable to G1 and significantly increased clonal richness compared to G5 (Figure 3B), indicating that pSer:nano-alum changed the overall B cell composition of the GC responses compared to pSer:alum.

Clonal abundance curves were plotted for each group (Figure 3C), and the cumulative abundance of the top 5 clones was calculated for each animal (Figure 3D). For G1, the top 5 clones accounted for ~30% of the total B_GC_ response (median per group), while for G2 the median was significantly higher at ~80% (Figure 3D). Together, these parameters show that ED+SMNP recruited large numbers of clones with moderated immunodominance, whereas bolus+SMNP immunization recruited fewer total B cell clones and a small number of those clones dominated the GC response.

Ig isotype was determined for each sequenced BCR (Figure 3E). All ED groups had a higher frequency of class-switched isotypes (IgG1–4, IgA) than bolus immunization. For G2, over 50% of epitope specific B_GC_ cells at week 3 were IgM. This was in contrast to 90% of epitope-specific B_GC_ cells being IgG in G1 (Figure 3E). When interpreted with the GC kinetics data (Figure 2H, K), the class-switch frequency differences suggest that GC responses were delayed after bolus immunization compared to ED+SMNP (Figure 3E).

Overall, the antigen-specific BCR sequencing data showed that in GCs 3-weeks post-prime, only ED+SMNP recruited detectable BG18 type I cells, which correlated with a distinctly clonally rich, diverse, and rapid B_GC_ cell response compared to other groups. Regarding kinetics of the GCs, antigen- and epitope-specific B_GC_ frequencies showed that many groups still had low vaccine-specific GC responses at the 3-week time point, which subsequently peaked at 6, or even 10, weeks post-prime (Figure 2H, K). It therefore remained possible that the clonal composition of those more slowly amplifying B cell responses may evolve after week 3.

### Vaccine-specific T cells

**Figure 4:**
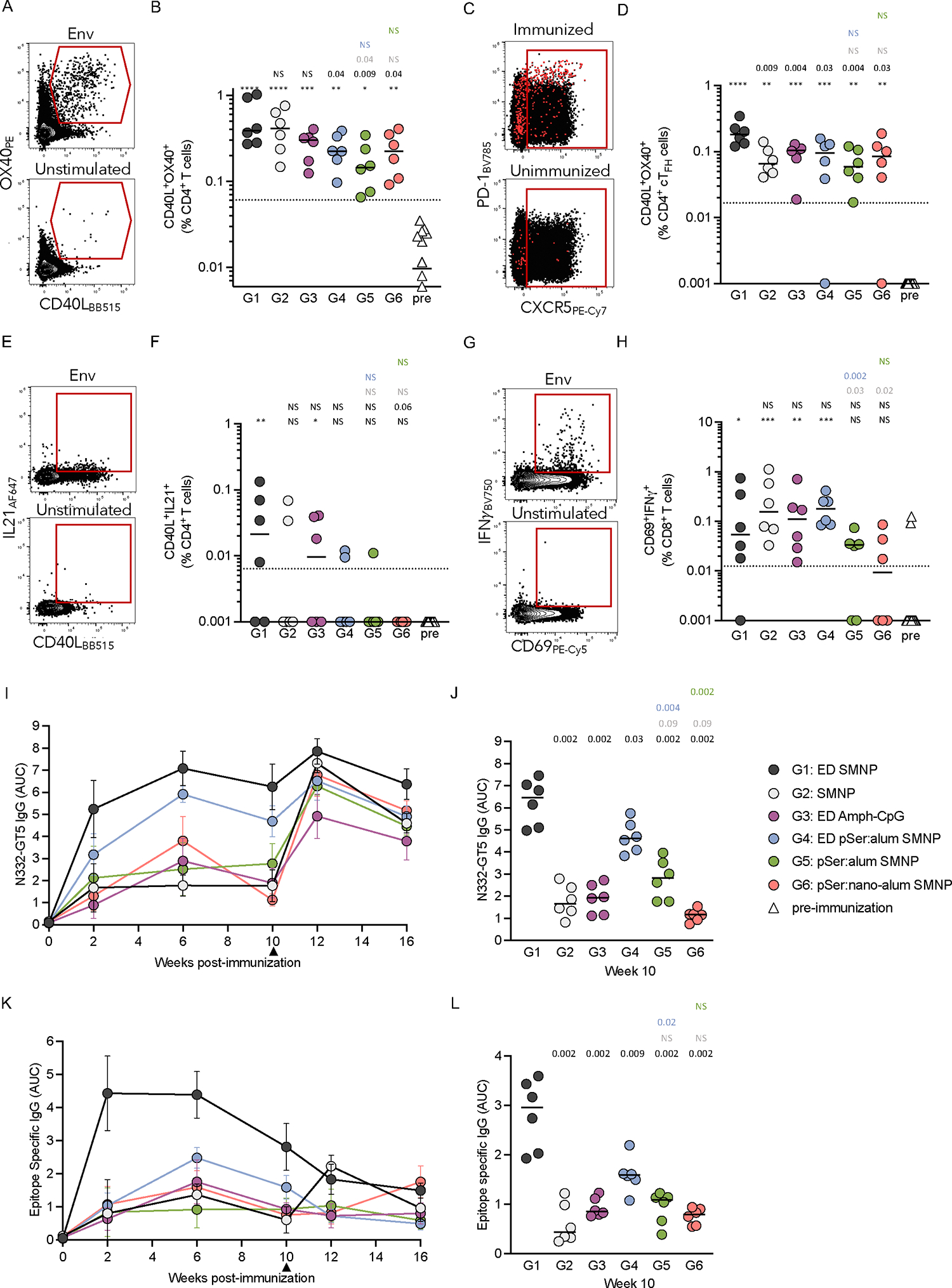
Vaccine-elicited T cell and serum IgG responses. **A)** Flow cytometry gating of AIM assay performed at week 2 using N332-GT5;(Env) overlapping peptide pools or a DMSO control (unstimulated). Full gating shown in Figure S6A, and **B)** AIM^+^(CD40L^+^OX40^+^) T cell frequency of total CD4^+^ T cells. **C)** Flow cytometry gating of AIM^+^cT_FH_, and **D)** frequency of total CD4^+^cT_FH_ cells. Red dots are Env+. **E)** Flow cytometry gating of AIM^+^ICS^+^(CD40L^+^IL21^+^), and **F)** frequency of total CD4^+^ T cells. **G)** Flow cytometry gating of AIM^+^ICS^+^(CD69^+^IFNg^+^), and **H)** frequency of total CD8^+^ T cells. **I)** Longitudinal area-under-the-curve (AUC) of antigen-specific serum IgG, and **J)** AUC at week 10 per animal. **K)** Longitudinal AUC of epitope-specific serum IgG, and **L)** AUC at week 10 per animal. Mean and SD plotted in (I,K), all others are median. Statistical significance was tested using unpaired two-tailed Mann-Whitney tests as described in Methods, NS: p>0.1. Asterixis in (B,D,F,H) indicate statistical significance compared to the pre-immunizations samples: NS>0.05, *<0.05 ,**<0.01, ***<0.001, ****<0.0001. See also Figure S6 and S7.

Germinal center responses require CD4 T cell help, specifically T_FH_ cells^51^. N332-GT5 specific T cell responses were measured by activation-induced marker (AIM) and intracellular cytokine staining (ICS) assays at 2-weeks post-immunization for multiparametric enumeration and functional analysis of the CD4 T cell response (Figure 4A; S6A). N332-GT5–specific (AIM^+^, CD40L^+^OX40^+^) CD4^+^ T cell responses were detected in all six groups above the pre-immunization levels (Figure 4B). The three pSer:alum groups had the lowest T cell responses (G4-G6, Figure 4B). Similar trends between groups were seen with other AIM parameters, and by ICS for IFNγ, granzyme B, IL2, and TNF (Figure S6B–F). Antigen-specific circulating T_FH_ (cT_FH_) cells were measured (Figure 4C). G1 had a significantly higher frequency of antigen-specific (AIM^+^CD40L^+^OX40^+^) cT_FH_ cells compared to all other groups (Figure 4D; S6G). The remaining groups had similar frequencies of cT_FH_ cells, despite clear differences in GC-T_FH_ frequencies. For G4 and G6, both had ~3-fold higher frequencies of GC-T_FH_ compared to other groups but similar levels of cT_FH_ (Figure 4D, 2E–F, week 3).

AIM+ICS was performed to assess whether the vaccine-specific cells were able to produce IL-21 (AIM^+^ICS^+^, CD40L^+^IL-21^+^, Figure 4E). Detectable IL-21^+^ antigen-specific CD4 T cells were identified in some animals, with only G1 (4/6) and G3 (3/6) having significantly higher responses compared to pre-immunization (p = 0.001 (G1) and 0.013 (G3), Figure 4F).

CD8^+^ T cell responses were examined by AIM+ICS to determine whether these protein immunizations primed antigen-specific CD8^+^ T cells (AIM^+^ICS^+^, CD69^+^IFNγ^+^). All groups had at least 3 animals with detectable IFNγ-secreting CD8^+^ T cells, with all but G5 and G6 significantly higher than background (Figure 4G–H).

Altogether, these data show that all immunization strategies primed antigen-specific CD4^+^ T cells. cT_FH_ cell and IL-21 responses were particularly robust after ED+SMNP immunization, consistent with the larger early GC responses seen in G1, which may have contributed to the strongest early recruitment of rare BG18 type 1 precursors in G1.

### ED+SMNP led to rapid serum IgG

Serum IgG titers were measured longitudinally by ELISA (Figure 4I–L; S7A–C). G1 had rapid induction of high titers of N332-GT5 specific serum IgG at week 2, which peaked at week 6 and remained high at the time of boosting (Figure 4I). In contrast, G2 had low levels of serum IgG that remained stagnant after seroconversion at week 2 (Figure 4I). G1 maintained significantly higher titers at week 10 than all other groups (Figure 4I–J). IgG AUC values for G1 were 5-times higher than G2 at week 10, demonstrating a significant enhancement in the ability of ED immunizations to induce high titers of antigen-specific IgG post-priming compared to bolus. Epitope-specific serum IgG in G1 was 6-times higher at week 10 compared to G2 (IgG AUC; Figure 4K–L. IgG titers; S7B). G6 (nano-alum) exhibited the lowest IgG titers post-prime (Figure 4K–L), contrasting with the relatively strong GC responses measured (Figure 2). When post-prime antigen- and epitope-specific B_GC_ responses were graphed together with IgG AUC values at week 10, G6 is a clear outlier (Figure S7D–E). Thus, while for most groups there is a strong association between GC and serum antibody responses, nano-alum immunization generates unusually divergent B_GC_ and plasma cell (B_PC_) responses.

### ED primed larger B_mem_ responses

**Figure 5:**
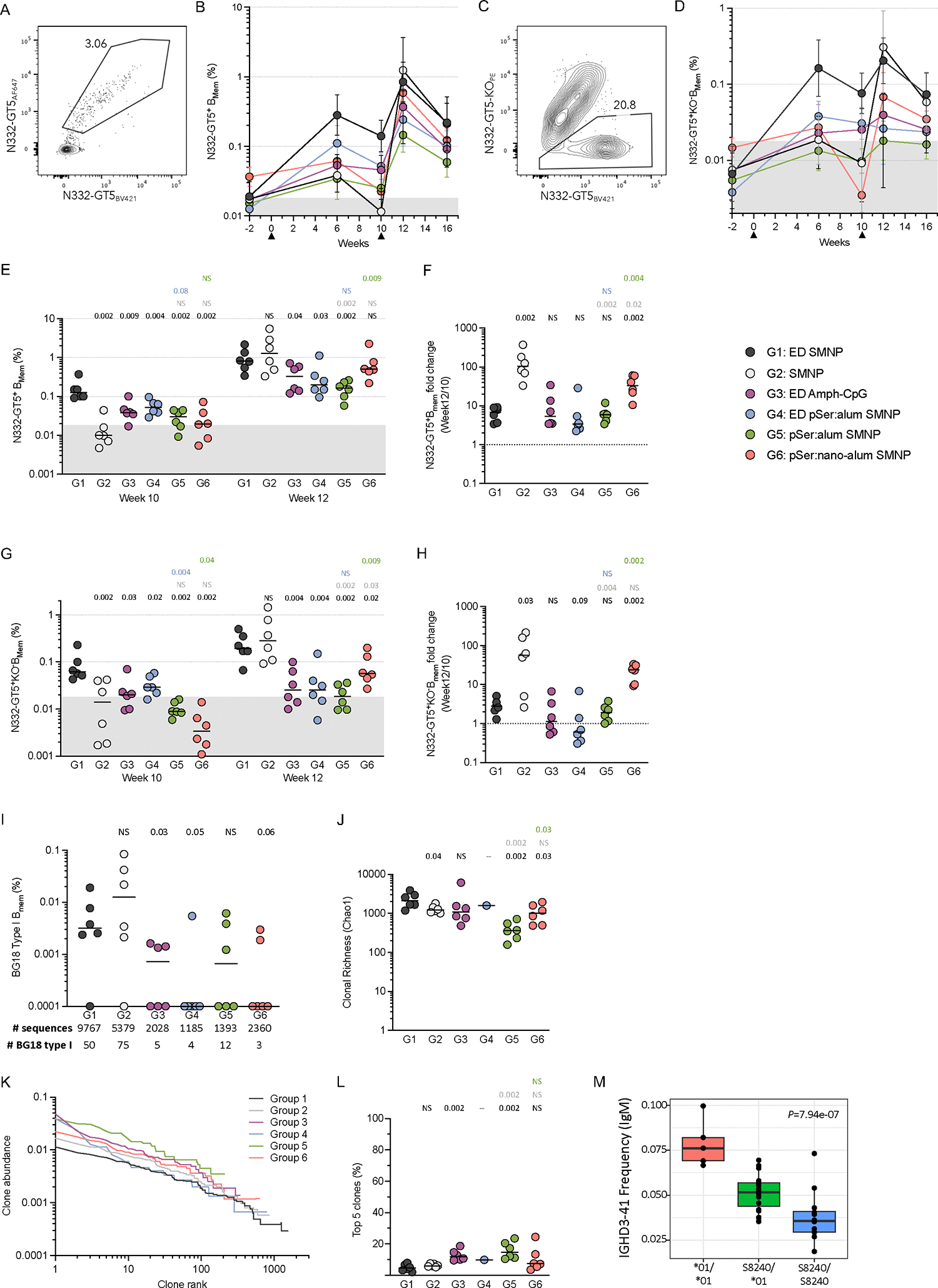
Antigen-specific B_mem_ responses. **A)** Flow cytometry gating of antigen-specific B_mem_, and **B)** longitudinal frequency of total B cells. Full gating shown in Figure S8A. **C)** Flow cytometry gating of epitope-specific B_mem_, and **D)** longitudinal frequency of total B cells. **E)** Week 10 and 12 antigen-specific B_mem_ frequency by animal, and **F)** fold change from week 10 to 12. **G)** Week 10 and 12 epitope-specific B_mem_ frequency by animal, and **H)** fold change from week 10 to 12. **I)** Frequency of BG18 type I B_mem_ among total B cells at week 12. Table indicates total and number of BG18 type I. **J)** Clonal richness of the B_mem_ from week 12. **K)** Clonal abundance curves for each group, and **L)** cumulative abundance of the top-5 clones per animal. **M)** Frequency of IGHD3–41 usage in IgM^+^ naive B cells, plotted by genotype. Lines represent medians. Statistical significance was tested using unpaired two-tailed Mann-Whitney tests as described in Methods, NS: p>0.1. Gray regions (B,D,E,G) represent B_mem_ LOD. See also Figures S7,S8,S9.

To evaluate the immune memory elicited by the different adjuvants and vaccine delivery mechanisms, antigen- and epitope-specific IgD^−^ memory B (B_mem_) cells were interrogated longitudinally by flow cytometry (Figure 5A, C; S8A). G1 had the highest B_mem_ frequencies for both antigen-specific and epitope-specific cells at week 6 (Figure 5B, D). Most groups had modest antigen- and epitope-specific B_mem_ cells at week 6 or 10 post-prime (Figure 5B, D, E, G). At week 10, G1 had a significantly higher frequency of epitope-specific B_mem_ cells compared to all other groups and was 10-fold higher than G2 (Figure 5G). Despite antigen-specific GC responses similar to G1 (Figure 2I), G4 and G6 had significantly lower levels of antigen-specific B_mem_ at week 10 compared to G1 (Figure 5E, G).

After boosting at week 10, an increase in antigen-specific B_mem_ frequencies were observed for all groups. Unexpectedly, G2 exhibited a median antigen-specific B_mem_ frequency equal to G1 at week 12, despite having the lowest pre-boost frequency (Figure 5E). The median epitope-specific B_mem_ frequency at week 12 was also equal between G2 and G1 (Figure 5G). To explore this change further, the week 10 to 12 B_mem_ fold change was calculated for each animal (Figure 5F, H). G2 had a 100-fold increase in B_mem_ (Figure 5F), ~20-times greater than the increase observed for G1. G6 had the 2^nd^ lowest B_mem_ frequency post-prime, and exhibited a 30-fold increase at week 12 (Figure 5F). Similar results were observed with epitope-specific B_mem_ (Figure 5H). Despite major shifts in B_mem_ frequencies, the LN FNA antigen- and epitope-specific B_GC_ frequencies were mostly unchanged from week 10 to 12 (Figure 2E, H). Notably, the G2 B_GC_ frequencies at week 12 were still 5- and 20-fold lower than G1 for antigen- and epitope-specific B_GC_ cells respectively, despite similar B_mem_ frequencies (Figure 2E, H). In sum, ED immunizations with SMNP led to the highest frequencies of antigen- and epitope-specific B_mem_ cells post-prime; however, booster immunization drastically increased the frequency of B_mem_ in response to bolus+SMNP or nano-alum with SMNP, suggesting a substantial shift in the parameters regulating immunogenicity between the prime and booster immunization.

### Bolus+SMNP induced a high frequency of BG18 type I B_mem_ cells post-boost

Epitope-specific B_mem_ were sorted and sequenced from PBMCs at week 12. BG18 type I B_mem_ sequences were found in 5 of 6 animals in G1 (Figure 5I; Table S2), consistent with the GC data (Figure 3A) and results from the previous study^25^. Unexpectedly, while no BG18 type I B_GC_ were detected in G2 at week 3, G2 had a similar frequency of BG18 type I B_mem_ at week 12 compared to G1 (Figure 5I). The remaining groups inconsistently developed BG18 type I B_mem_, which were only detected in 9 of 24 animals (Figure 5I). Of note, despite robust GC responses in G4 and G6, only one animal from G4 and two from G6 had detectable BG18 type I B_mem_ (Fig 5I). The results observed for BG18_20–21AA_ and potential type III cells were similar to BG18 type I responses across the groups, with the caveat that G2 appeared to have more sporadic responses (Figure S8B–C).

B_mem_ were more clonally rich and diverse across all groups compared to B_GC_, likely due to the inherently less clonal nature of circulating B_mem_ cells compared to B cells participating in a GC reaction in a single LN (Fig 5J; S8D compared to Figure 3B–E; S5D). G1 had the greatest B_mem_ clonal diversity across multiple measures (Fig 5J–L; S8D). However, similar to B_mem_ frequencies above, G2 was much more similar to G1, with only clonal richness showing a significant difference between G1 and G2 (Fig 5J–K; S8D). Rarefaction analysis revealed that G1 and G2 had similar sample coverage (Figure S8E). Immunodominance in immune memory, as assessed by the dominance of the top 5 B_mem_ clones, was lowest in G1, G2, and G6 (Figure 5K–L).

All animals carried *IGHD3–41* in their genome and were determined to have alleles capable of making BG18 type I responses using reading frame 1 or 3 (Table S3). Animals that were *01/*01 homozygotes used *IGHD3–41* at a higher frequency in naive IgM^+^ B cells than animals that were *01/*01_S8240 heterozygotes or *01_S8240/*01_S8240 homozygotes (~1.5- and 2-fold higher, respectively. p=7.94×10^−7^, Figure 5M). The three genotypes were well distributed throughout the groups (Figure S9A–B) and all three were capable of making BG18 type I responses after N332-GT5 vaccination (Figure S9B–C). Thus, *IGHD3–41* genotype influence on B cell precursor frequencies did not explain the majority of the inter-group BG18 type I B cell response differences observed. Overall, the totality of the post-boost B_mem_ data showed that bolus or ED immunizations with SMNP could prime rare BG18 type I precursors after N332-GT5 immunization. All other combinations inconsistently led to priming of BG18 type I precursors. In addition, a homologous booster immunization after a bolus N332-GT5+SMNP prime could generate highly expanded BG18 type I B_mem_ cells and a more clonally rich and diverse response than initially expected.

### Serum IgG levels inversely predicted booster immunization outcomes

**Figure 6:**
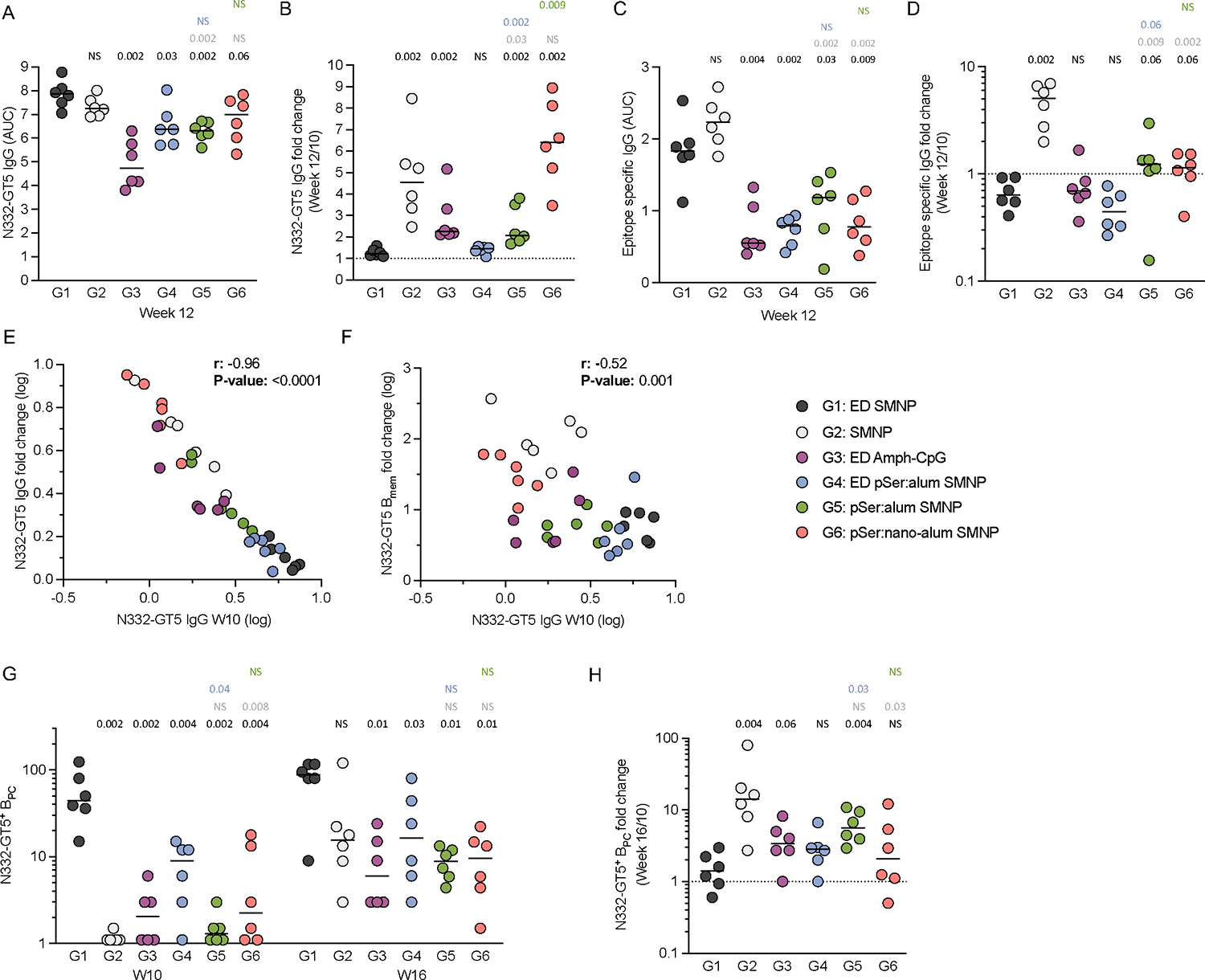
Level of antigen-specific circulating IgG predict boost outcomes **A)** AUC of antigen-specific serum IgG at week 12, and **B)** fold change from week 10 to 12. **C)** AUC of epitope-specific serum IgG at week 12, and **D)** fold change from week 10 to 12. **E)** Antigen-specific IgG fold change and week 10 IgG AUC correlation. **F)** Antigen-specific B_mem_ fold change from week 10 to 12 and week 10 IgG AUC correlation. **G)** Number of antigen-specific BM-B_PC_ per million BM cells at weeks 10 and 16, and **H)** fold change from week 10 to 16. r and p-values (E,F) are from pearson correlation analysis. Statistical significance was tested using unpaired two-tailed Mann-Whitney tests as described in Methods, NS: p>0.1. See also Figure S7 and S10.

Post-boost serum IgG titers were quantified (Figure 4I, K). G2 antigen-specific IgG increased 5-fold from week 10 to 12, while only a modest change was observed for G1 (Figure 6B, D). At week 12, antigen- and epitope-specific IgG AUC values for G2 were similar to G1 (Figure 4I, K; 6A, C). A clear inverse relationship was observed for all groups between pre- and post-boost IgG, whereby the higher the week 10 IgG the smaller the fold increase after boosting (r = −0.96. P < 0.0001. Figure 6E). A similar relationship was seen with epitope-specific IgG (r = −0.71. P < 0.0001. Figure S10A). A significant inverse relationship was also seen between the B_mem_ fold changes (boost:pre-boost) and pre-boost serum IgG titers (week 10) across study groups (r=−0.52, p=0.001. Figure 6F). These data indicate that very high levels of circulating N332-GT5 specific IgG pre-boost at week 10 in G1 were associated with a blunted B cell recall response to homologous booster immunization, as observed in the modest increases in B_mem_ (Figure 5H), BG18 type I BCRs (Figure 5I), and serum IgG (**Figure GD**). In contrast, the low levels of pre-boost IgG in G2 allowed for strong responses to the booster immunization, involving a dramatic 100-fold increase in antigen-specific B_mem_, detectable BG18 type I B_mem_ in most animals, and large increases in serum IgG.

### Bone marrow plasma cells

The potential durability of the humoral immune response was assessed by quantifying N332-GT5 specific bone marrow (BM) B_PC_ (Figure 6G; S10B–C). Only G1 developed substantial antigen-specific BM B_PC_ after priming (week 10, Figure 6G), with levels ~40-fold greater than G2. All groups showed an increase in BM B_PC_ 6-weeks after the booster immunization (week 16, Figure 6G–H). G2 had the most dramatic increases from week 10 to 16 (Figure 6H), which correlated with the strength of the IgG and B_mem_ response post-boost. G2 had the second highest BM B_PC_ responses post-boost, although still 5-fold lower than G1 (Figure 6G). However, the ELISA data (Figure 4I, K) indicated much of the G2 post-boost B_PC_ response was short-lived, while both antigen- and epitope-specific IgG appeared more stable in G1, evident by the more rapid decline in titers from week 12 to 16 for G2. The BM B_PC_ data reinforced that SMNP adjuvanted bolus or ED immunizations led to the strongest outcomes across all measures of humoral immunity.

### BG18 type I BCRs had more SHM and higher affinity for boosting candidates

Similar levels of circulating BG18 type I B_mem_ cells were observed after booster immunization for both G1 and G2 (ED and bolus+SMNP, Figure 5I). BG18 type I B_GC_ cells were undetectable in G2 at week 3, and antigen-specific B_GC_ cells were 50-fold lower in G2 than G1 at week 3 (Figure 3A, 2H). Thus, the week 3 and week 12 BG18 type I cell outcomes were discordant. We therefore endeavored to better understand the underlying immunological processes resulting in these large changes. This topic was of particularly interest given the very rare precursor cells involved. Three plausible scenarios were considered. One possibility was that many fewer BG18 type I cells were primed in G2 compared to G1, but that post-boost those few clones in G2 massively expanded because of the high affinity interaction with N332-GT5 and the absence of competing circulating IgG. A second possibility was that BG18 type I cells were only primed in G2 after the booster immunization, whereas BG18 type I cells were primed and expanded after the initial ED immunization in G1. A third possibility was that the BG18 type I cells were recruited into GCs substantially later post-prime for G2 compared to G1, given that G2 had low vaccine-specific GC responses at week 3 that subsequently peaked 6–10 weeks post-prime (Figure 2H, K)), with a booster immunization required to expand the BG18 type I cells sufficiently to be detectable in G2. We resolved these three scenarios through a series of experiments, including assessment of clonality, quantitation of somatic hypermutation (SHM), and measurements of antibody function derived from G1 and G2 BG18 type I BCRs.

**Figure 7:**
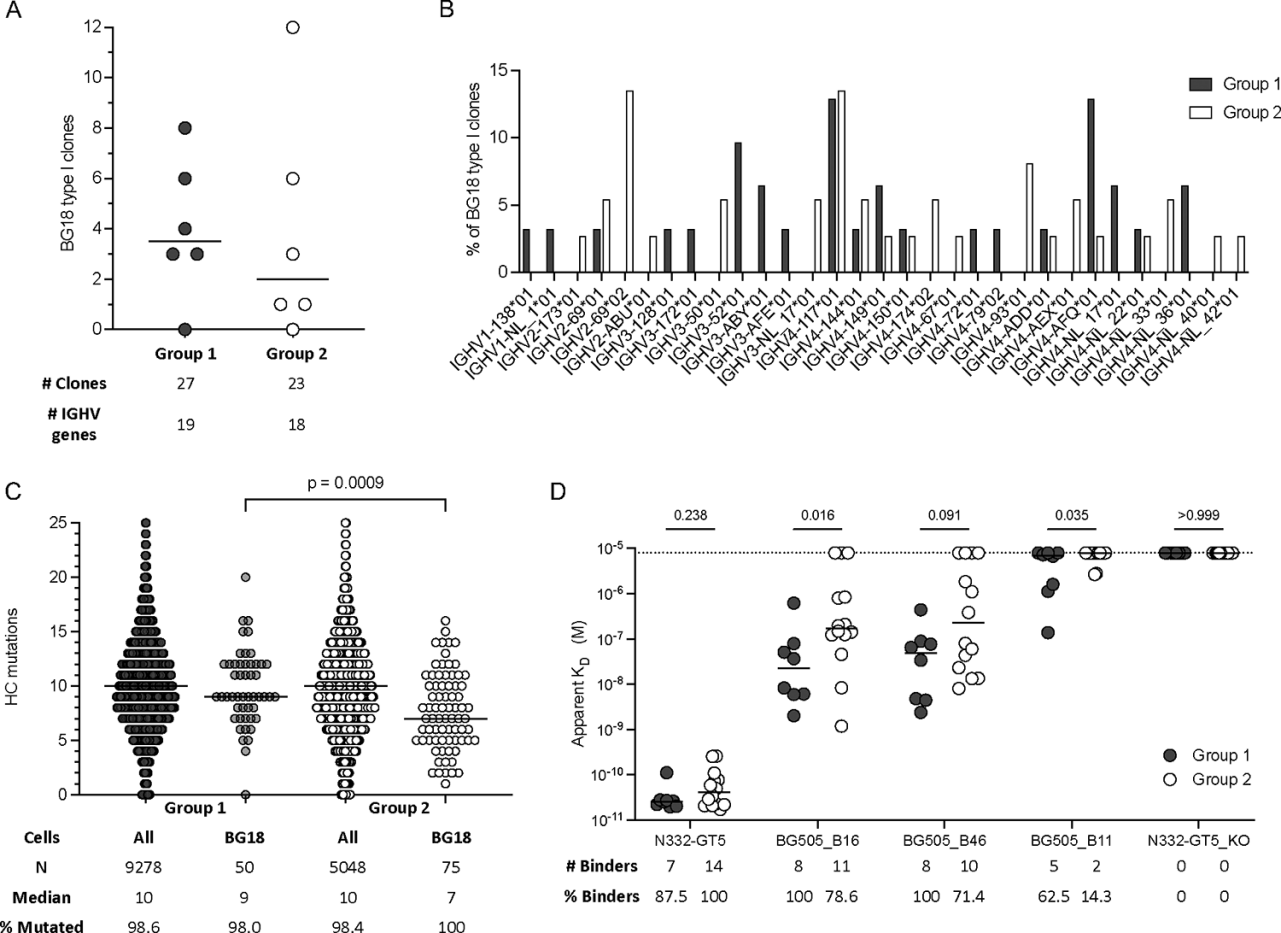
ED+SMNP leads to more SHM and higher affinity for boosting candidates. **A)** Number of BG18 type I clones and IGHV genes used per animal. **B)** Frequency of IGHV gene usage by BG18 type I clones. **C)** Heavy-chain nucleotide mutations per cell. Total and BG18 type I BCRs are shown with the number of sequences, median mutations, and percent of mutated sequences. **D)** Binding affinities (Apparent K_D_) for a subset of BG18 type I BCRs to N332-GT5 and three potential boosting immunogens. Number and percent of binders are listed (G1: n = 8, G2: n = 14). Dotted line represents the LOD for this assay. Statistical significance (C,D) was tested between groups using unpaired two-tailed Mann-Whitney tests. See also Figure S11.

First, BCR sequence analysis revealed that responding B cells in both G1 and G2 had similar numbers of total BG18 type I clonal families (Figure 7A), with equally diverse usage of different IGHV genes (Figure 7B) and light chain usage (Figure S11A). These data indicated scenario #1 was unlikely.

Scenario #2 could be tested by examination of SHM between G1 and G2. In that scenario, BG18 type I BCRs from G2 B_mem_ at week 12 would have low or no SHM, whereas those from G1 would have substantial SHM. As a control, the total antigen-specific B_mem_ were expected to have similar mutations between groups. Scenario #3 could also be resolved by SHM analysis, as cells experiencing less time in GCs would be expected to have less SHM. SHM was analyzed for all groups (Figure S11B–C). Overall SHM HC mutation counts of antigen-specific B cells were similar between G1 and G2 (Figure 7C), with > 98% of BCRs containing HC SHM in both groups, consistent with the vast majority of the B_mem_ derived from the antigen-specific B_GC_ responses observed in both groups post-prime (Figure 2H–I). Notably, when BG18 type I BCRs were examined, > 98% of BCRs also contained HC SHM in both groups (Figure 7C). G2 did have more BG18 type I BCRs that had 1–4 HC mutations at week 12 (Figure 7C); however, all but one of these cells belonged to clonal families with BCRs that had >5 mutations, making it unlikely that they were primed just two weeks earlier (Table S2). These results suggested that scenario #2 was implausible to explain the majority of the BG18 response in G2. In contrast, when SHM of BG18 type I BCRs was quantitatively assessed, G2 had fewer HC mutations per BCR than G1 (p = 0.0009, Figure 7C), while SHM of non-BG18 BCRs was indistinguishable between G1 and G2 (Figure 7C). This difference was also significant at the amino acid level for BG18 type I HC (p = 0.003, Figure S11D). Thus, a parsimonious interpretation of the data is that BG18 type I B cells from G2 likely participated in GC reactions for a shorter period of time after priming than did the BG18 type I cells from G1, based on SHM levels.

To examine the functional consequences of the SHM differences, a selection of BG18 type I BCRs from each group were produced as IgG. Antibodies from both G1 and G2 exhibited very high affinity for N332-GT5, and no significant difference was observed between the groups (Figure 7D). All antibodies were epitope-specific, as expected, exhibiting no binding to N332-GT5KO (Figure 7D). Affinity to three heterologous Env trimer boosting candidates^9,25^ was determined. 100% of BG18-like antibodies from G1 had detectable affinity for heterologous booster candidate BG505_B16, the most similar antigen to N332-GT5, while only 79% (11/14) of the antibodies from G2 bound BG505_B16. BG505_B16 affinities were significantly stronger for G1 antibodies compared to G2 (p = 0.016, Figure 7D). Affinities were also significantly stronger for G1 antibodies compared to G2 for BG505_B11, the most native-like Env booster candidate tested (p = 0.035, Figure 7D). Neutralization was tested against a panel of V1 modified HIV pseudoviruses for the highest affinity antibodies (Figure S11E). As expected, no difference was observed in neutralization of the GT5-pseudovirus (Figure S11E). Adding back one glycosylation site reduced or completely abrogated neutralization to all antibodies and none could neutralize the pseudovirus with both glycosylation sites added back (Figure S11E). Overall, these SHM and binding data consistently indicate that BG18 type I precursors from G2 were primed by the initial immunization but spent less time participating in the GC reaction compared to G1, with functional consequences observed by reduced binding affinity for heterologous boosting candidates.

## DISCUSSION

Despite their value in generating high quality antibody responses, the processes governing recruitment of rare bnAb-precursor B cells, and competition between rare and common B cell specificities, are only partially understood^6^. These processes have primarily been studied in mice using precisely controlled frequencies of adoptively transferred B cells^4,9–12,60,61^. Here we explored recruitment and differentiation of rare B cells in an outbred, non-transgenic, model using an HIV germline-targeting priming immunogen and demonstrate that different antigen delivery strategies and adjuvants can extensively impact priming, expansion, differentiation, and affinity maturation of rare precursor B cells.

Kinetics of naive B cell recruitment may play a large role in the results seen here, particularly the early GC data. Finding BG18 type I cells in at least one animal in every group at week 12 indicated that some recruitment of rare precursors could occur under all six conditions tested, but was inconsistent at best in most cases. Despite all six groups inducing antigen-specific GC responses, only ED+SMNP (G1) drove early recruitment of BG18 type I precursors, detectable at week 3. In the case of the bolus immunization group, the cytometry data suggest that the BG18 cells may enter GCs later under those conditions. The SHM data also suggested that the BG18 type I cells from the bolus group spent less time in GCs than from the ED group, consistent with later recruitment of the cells. Those outcomes are consistent with early T cell help likely playing a crucial role in the kinetics of early GC development and diverse B cell recruitment to GCs^38,62,63^. This was evidenced by the most robust early GC-T_FH_ and cT_FH_ responses being observed in G1, which may be driving many of the other outcomes.

The BG18 cell frequencies seen amongst the week 12 B_mem_ cells were likely representative of the priming recruitment efficiency for each group by the initial immunization. We previously estimated it was conceivable that ~1.2×10^9^ naive B cells could be “screened” in LNs for antigen-recognition under optimized immunization conditions in humans^6^. We can extend this calculation to RMs using updated data. There are ~5 million B cells per RM inguinal LN. We and others have shown that multiple LNs in a drainage region will uptake antigen^39,64–67^ and after ED immunization with SMNP this may be 3–5 LNs in a region^39^ (~20 million B cells). It has also been shown that after SMNP immunization antigen drains to deeper LN clusters, probably doubling the number of LNs engaged (~40 million B cells)^68^. Immunizations in this study were done bilaterally, doubling the number of LNs engaged (~80 million B cells). Naive B cells are constantly moving through lymphatics, and in humans exhibit a LN dwell time of 10–16 hours^69–71^. After a bolus immunization it is reasonable to assume that naive B cells can be recruited during the first 48-hours^72^, which would perhaps represent 4x turnover of the naive B cell population during that timeframe. The data suggest that after ED immunization naive B cells may be recruited for 2- to 3-weeks^37,38,73^, which plausibly represents 28 to 42x turnover of the naive B cell population per LN during that time. Furthermore, influx of cells into LNs after immunization can lead to swelling and 2- to 4-fold increased cell numbers^72^. Those estimations result in a calculated number of ~9×10^9^ naive B cells potentially being “screened” *in vivo* for antigen binding after ED+SMNP immunization in RMs.

Although we do not know the true number of BG18 type I precursors that can bind to and be activated by N332-GT5 in RMs, in humans, BG18 type I precursors were found at ~1-in-50 million naive B cells^12,25^. That number is likely to be 2–8-fold lower in RMs (1-in-100–400 million^25^). Taken together, these calculations estimate that between 23 and 90 BG18 type I naive B cells are potentially screened for antigen binding in LNs after N332-GT5 ED immunization with SMNP in RMs.

Experimentally, we observed that in G1 there was a median of 3.5 BG18 type I clonal families per animal in the B_mem_ compartment. Using rarefaction analysis, we estimated that ~50% (range of 40–75%) of the B_mem_ repertoire was sampled in the animals from G1. This indicated that the true number of BG18 type I clonal families present in each animal was likely double that observed experimentally (median 7, range 0–16). The numbers are likely much lower in non-SMNP and bolus immunization animals, which underscores the difficulty in eliciting these types of rare responses.

One of the more striking outcomes was the large boosting response seen for the bolus immunization group, which inversely correlated with circulating IgG titers pre-boost, implicating antibody feedback as a major factor in the booster immunization outcomes. Antibody feedback has recently seen a resurgence in research^74–77^. Antibody feedback can exert influence through multiple mechanisms, including antigen clearance, which can occur through Fc receptor mediated uptake of immune complexes^77^. In a recent knock-in mouse study of N332-GT5, authors found excess antigen could overcome antibody-dependent clearance of antigen by effectively depleting circulating IgG, allowing for better secondary GC responses^78^. IgG against dominant epitopes can subsequently enhance responses to sub-dominant epitopes by re-focusing GC responses^74,76,79–82^. Slow delivery immunization strategies likely enhance priming of more diverse B cell responses due to engagement of this mechanism involving the earliest antibodies^37,38,73^. It is conceivable that in the bolus group, low circulating off-target IgG could lead to immune complex formation after booster immunization and deposition on FDCs, while the lack of epitope-specific IgG allows for recall B_mem_ against the desired epitope^83^. Extremely high titers of antigen- and epitope-specific IgG in Group 1 appear to have led to both antigen clearance and almost complete epitope masking of the BG18 epitope from B_mem_ recall.

When pSer modification was used to deliver a native HIV Env trimer, improvements in both antigen-specific GC B cells and IgG were seen^43 45^. It was therefore unexpected that recruitment of BG18 type I cells was poor when delivering pSer modified N332-GT5 by ED. This group had some of the highest GC responses, but those large numbers of B_GC_ did not translate into efficient recruitment of rare BG18-like precursors. The early antibody response to soluble native-like Env trimers is dominantly focused on the irrelevant base of the immunogen^37,38,42^. By contrast, N332-GT5 is engineered with a prominent glycan hole around the target epitope, relatively distant from the Env base. Adoptive transfer mouse models of rare precursor B cell recruitment using this immunogen have shown that the Env trimer base is no longer dominant in this scenario; instead, responses in the vicinity of the new glycan hole dominate^12^. In that situation, antibody feedback may hinder rather than help the development of the desired on-target B cell response.

The comparison between ED and bolus immunization here is additionally valuable as it informs ongoing clinical development of N332-GT5 as an HIV vaccine candidate. Currently, the first-in-human clinical trial for both N332-GT5 and SMNP is ongoing (NCT06033209). In that study, ED and bolus immunizations with N332-GT5+SMNP are being compared head-to-head. The results presented here will help inform the analysis of that trial and provide a benchmark for comparing the priming efficiency in humans. Our results suggest that bolus immunization with suitably designed germline-targeting trimer immunogens and appropriate adjuvants may suffice for inducing bnAb precursor responses in humans. The higher affinity for booster candidates seen after ED+SMNP suggest ED may allow for earlier introduction of native-like antigens in the immunization sequence. These data demonstrate that adjuvant selection is key, and the capacity of SMNP to induce large GC responses recruiting highly diverse B cells, including very rare precursors, will be an important component of any protein-based germline-targeting immunization regimen.

The next step for development of a germline-targeting vaccine will rely on shepherding of primed precursors towards bnAbs. Therefore BG18-class B cells induced by N332-GT5 must be able to engage more native-like HIV Env trimers, without being blocked by serum antibodies. At this time, the *in vivo* affinity threshold for boosting primed BG18 type I responses in NHPs is not clear, but knock-in mouse data suggests that even modest affinities can lead to robust boosting^9^. We hypothesize that BG18 type I cells after ED or bolus+SMNP+N332-GT5 have sufficient affinity to the tested immunogens as to be boosted *in vivo*. However, higher affinity for booster candidates seen after ED+SMNP suggest ED may allow for earlier introduction of native-like antigens in the immunization sequence.

In sum, here we demonstrated that both ED and bolus immunization can lead to priming and expansion of rare BG18 type I B cells in NHPs when adjuvanted with SMNP and represent the first direct study of rare B cell recruitment under multiple conditions in an outbred, non-transgenic, animal model. In the context of germline-targeting immunizations, the quality of the B cells was higher in the ED group, consistent with more rapid recruitment of B cells to GCs and more extensive SHM in GCs, along with differential regulation of the rare B cell responses by the varying concentrations of circulating IgG generated by the different immunization conditions.

### Limitations of the study

Amph-CpG, performed worse than SMNP in ED immunization. Amph-CpG has shown promise in modulating immune responses against SARS-CoV-2 vaccines in NHPs^50^. Further work is needed to elucidate how engaging different TLRs leads to differences in adjuvanticity compared to SMNP. The nano-alum group exhibited unique immunological characteristics suggesting that nano-alum may have a distinct mechanism of action compared to traditional alum and further study is warranted. It remains an interesting observation that although circulating titers at week 10 predict the post-boost B_mem_ frequencies, there is no correlation with the antigen- and epitope-specific B_GC_ frequencies from week 10 to 12.

## Resource Availability

### Lead Contact

Requests for further information and resources should be directed to the lead contact, Shane Crotty (shane@lji.org).

### Material availability

This study did not generate new reagents.

### Data and code availability

- The sequencing data have been deposited in the SRA database under the following accession numbers: SRA:PRJNA1233342(for epitope-specific B cell), SRA: PRJNA1231401 and PRJNA1207082 (for IgM repertoire and long-read genomic).
- Code used to analyze the IgM repertoire data can be found at https://github.com/BosingerLab/AIRRSeq_IGHD_freq_NHP.
- Any additional information required to reanalyze the data reported in this paper is available from the lead author upon request.

## STAR METHODS

### Adjuvant production and formulation

Alhydrogel aluminum hydroxide adjuvant was purchased from Invivogen and used as received. SMNP adjuvant was synthesized and characterized as previously described^39^. Amph-CpG consisting of the class B CpG 7909 sequence (5’-TCG TCG TTT TGT CGT TTT GTC GTT-3’) conjugated at the 5’ end to a diacyl lipid tail (ref ^84^)was prepared by solid phase synthesis by Axo Labs.

Nano-alum was generated by dispersing aluminum hydroxide adjuvant in the presence of a phosphate-containing PEG-lipid surfactant. Alhydrogel (10 mg, Invivogen) was diluted to 1 mg/mL in deionized water and combined with 10 mg of 1,2-distearoyl-sn-glycero-3-phosphoethanolamine-N-[amino(polyethylene glycol (PEG))-2000] (Avanti Polar Lipids). The mixture was transferred to a 20 mL glass scintillation vial and cooled to 4°C in ice water. While still on ice, the mixture was sonicated with a Misonix Microson Ultrasonic Cell Disruptor XL2000 probe tip sonicator for 30 minutes, during which it transitioned from opaque to opalescent. Care was taken to position the sonication probe deep enough in the mixture to prevent foaming. Output power at the start of the sonication was set to ~20, with the power turned down as sonication progressed and the solution transitioned from opaque to translucent. To remove excess alum aggregates, the sonicated material was then incubated at 25°C for five days to enable precipitation of any residual non-stabilized alum. The supernatant containing the suspended nano-alum was collected, and nanoparticle formation was verified via dynamic light scattering on a Malvern Zetasizer Nano instrument. Nano-alum used in this study was measured to have a Z-average diameter of ~70 nm and a low PDI.

### Protein production and pSer modification

For the expression of the N332-GT5 immunogen, a stable CHO cell line clone was developed utilizing the Leap-In transposon expression system (ATUM, US10041077). The DNA sequence encoding the N332-GT5 trimer and its corresponding signal peptide was first synthesized. Subsequently, expression constructs were designed and engineered based on the proprietary Leap-In transposon platform. The production of the trimer protein involved comprehensive upstream cell culture and downstream purification process development, followed by scale-up to large-scale cGMP manufacturing (details to be published in a forthcoming article). With the exception of the signal peptide, the amino acid sequence of this trimer, matches the N332-GT5 protein that was reported previously^25^.

The N332-GT5 trimer used for pSer conjugation contained the C-terminal linker sequence “GTKKKC”, previously optimized for Env trimers and referred to as nohis8^43^, placing a free terminal cysteine for subsequent pSer peptide coupling. The trimer was co-transfected with furin into 293F cells and purified using Galanthus nivalis lectin affinity chromatography (Vector Laboratories) followed by SEC purification using Superdex 200 16/600 PG column (Cytiva).

Trimers that were used as SPR reagents were expressed with C-terminal 6xHis-tag and purified using a HIS-TRAP column followed by size exclusion chromatography (SEC) on a Superdex 200 Increase 10/300 column (Cytiva) as described previously^25^. N332-GT5 and N332-GT5-KO trimers that were used as biotinylated sorting reagents were expressed with a C-terminal His-tag and avi-tag, purified using a HIS-TRAP column followed by SEC and biotinylated using a BirA biotin-protein ligase reaction kit (Avidity, catalog no. BirA500) as described previously^25^. Compared to the immunogens, the N332-GT5 sorting probes lacked the 241 and 289 glycosylation sites.

N332-GT5 trimers with a C-terminal Cys were conjugated with maleimide-functionalized pSer peptide tags and characterized as previously described^43^. Briefly, peptides carrying 4 phosphoserines and N-terminally functionalized with a maleimide group (mal-pSer_4_) were produced by solid phase synthesis, purified by HPLC, and their mass confirmed by matrix-assisted laser desorption/ionization-time of flight mass spectrometry. N332-GT5-Cys trimers were gently reduced with tris(2-carbox- yethyl)phosphine then reacted with 5 molar equivalents of mal-pSer4 in tris-buffered saline, followed by centrifugal filtration to remove unconjugated peptide. The degree of modification of trimers was quantified using a Malachite Green Phosphoprotein Phosphate Estimation Assay Kit (Thermo Scientific). Alum binding of pSer-conjugated N332-GT5 was assessed by labeling the trimer with an AlexaFluor dye and assessing bound protein following mixing with alum (loading) or following incubation of loaded alum with 10% mouse serum in tris-buffered saline for 24 hr as previously described^43^. Antigenicity profiles of alum-bound pSer-N332-GT5 were assessed by capturing alum particles on ELISA plates followed by addition of pSer-trimer to allow binding, washing, and then probing with indicated dilutions of monoclonal antibodies as previously described^42^.

### Mouse immunogenicity testing of nano-alum

All mouse studies were carried out at MIT under an IACUC-approved protocol following federal, state, and local guidelines. Female BALB/c mice (Jackson Laboratory) 6–8 weeks of age were immunized s.c. bilaterally at the tail base with alum/pSer-trimer or nano-alum/pSer-trimer. For flow cytometry analysis, lymph nodes were harvested, crushed to form a single-cell suspension, and subjected to staining for viability (Zombie Aqua Fixable Viability Kit, BioLegend), CD4 (BV711, BioLegend, RM4–5 clone), B220 (PE-Cy7, BioLegend RA3–6B2 clone), CD38 (FITC, BioLegend 90 clone), CXCR5 (PE, BioLegend L138D7 clone), PD-1 (BV421, BioLegend 29F.1A12 clone), and GL7 (PerCP-Cy5.5, BioLegend GL7 clone). Antigen-specific staining was done using biotinylated trimers conjugated to either streptavidin-BV605 (BioLegend) or streptavidin-APC (BioLegend). Analysis was performed on a Becton-Dickinson Symphony flow cytometer, and the resulting data were processed using FlowJo software. Anti-trimer serum Ig titers were assessed by ELISA. 96-well plates (Costar Corning) were coated overnight at 4°C with 1 μg/ml (50 μl/well) of streptavidin in PBS. Coated plates were blocked with 2% BSA in PBS, and incubated overnight at 4°C. Plates were then washed and incubated with 1 μg/ml biotinylated trimer in blocking buffer (2% BSA in PBS) for 2 h at 25°C, following which serial dilutions of serum samples were added to the plates. After 2 h incubation, plates were washed and incubated with HRP-conjugated anti-mouse IgG diluted 1:5,000 in blocking buffer. The reaction mixture was incubated at 25°C for 30 min. Plates were washed and treated with 50 μL TMB substrate for 10 minutes followed by addition 1M H_2_SO_4_ stop solution. Optical densities were read on a microplate reader at 450 nm with background correction at 540 nm. Endpoint serum titers were determined as the reciprocal of the highest serum dilution leading to a signal 0.1 OD units above background.

### Non-human Primates and Study Design

Adult Indian rhesus macaques (Macaca mulatta) were housed at the Emory National Primate Research Center and maintained in accordance with NIH guidelines. All procedures were approved by the Emory University Institutional Animal Care and Use Committee (IACUC) under protocols 202100128 and 201700666. Animal care facilities are accredited by the U.S. Department of Agriculture (USDA) and the Association for Assessment and Accreditation of Laboratory Animal Care (AALAC) International. RMs for this study were of mixed sex, an average age of 5.5 years, and a weight range of 4–7.2kgs at the time of the priming immunization. Animals were pair housed for the duration of the study. Thirty-six NHPs were assigned to one of six experimental groups—each group was comprised of 6 RMs. The immunizations were as follows: Group 1 (ED+SMNP), Group 2 (SMNP), Group 3 (ED Amph-CpG), Group 4 (ED pSer:alum SMNP), Group 5 (pSer:alum SMNP), and Group 6 (pSer:nano-alum SMNP). Immunizations were administered subcutaneously (s.c.) in the left and right mid-thighs at weeks 0 and 10. A total of 100ug of N332-GT5 were administered in each immunization (50ug each side). The ED immunizations were performed as previously described ^25, 37^. Seven immunizations were given, administered every other day over the course of 12 days with an exponentially increasing dose of antigen and adjuvant for a total of 100ug antigen (0.1, 0.215, 0.58, 1.575, 4.28, 11.65 and 31.6 ug per side for the antigen, with adjuvants dosed in the same exponentially-increasing pattern). SMNP was dosed at 375mg per injection site for all SMNP-containing groups. All alum-containing adjuvant (alum and Nanoalum) were dosed at 500 μg per injection site. This is the alum dose that has been previously tested in pSer-based HIV Env immunizations in mice and RMs^43,45^. The Amph-CpG was administered at a dose of 2.5mg per injection site which was the optimal dose determined from a previous RM immunization experiment using SARS-CoV-2 RBD base protein immunogen^50^.

Veterinarians performed fine need aspirates (FNAs) to bilaterally sample lymph nodes (LNs) in the RMs. Palpation was used to identify draining LNs. A 22-gauge needle with an attached 3 ml syringe was passed into the LN a maximum of five times. Samples were then placed into RPMI media with 10% FBS and 1% Penicillin/Streptomycin. If red blood cell contamination was observed, an Ammonium Chloride-Potassium lysing buffer was used. Samples were counted, frozen down, and stored in liquid nitrogen until the time of analysis. Blood was collected throughout the study in NaCitrate CPT tubes (BD biosciences) for peripheral blood mononuclear cells (PBMCs) and plasma isolation and frozen. Serum was collected via serum clot tubes and frozen. Bone marrow aspirates were collected in heparin coated tube and analyzed via ELISPOT fresh. Animals were treated with anesthesia (ketamine 5–10 mg/kg or telazol 3–6 mg/kg) and analgesics for s.c. immunizations, LN FNA, bone marrow aspirates, and blood draws as per veterinarian recommendations and IACUC approved protocols. After completion of the proposed study and approval of the vet staff, animals were released to the center for reuse by other researchers.

### ELISA analysis of serum IgG titers

Serum IgG titers were measured by ELISA. Nunc MaxiSorp plates were coated with streptavidin 18 hr at 4°C and blocked with 2% BSA in PBS. Blocked plates were washed with washing buffer (PBS with 0.05% Tween-20) and incubated with Avi-tagged biotinylated antigen 18 hr at 4°C. Plates were washed again and serial dilutions of sera were added to plates and incubated for 2 hr at 25°C, following which the plates were washed and incubated with anti-rhesus IgG-HRP (BioRad). ELISA plates were developed with TMB substrate and stopped by sulfuric acid 2 M. Absorbance values at 450 nm were measured on a Tecan Infinite 200PRO plate reader, with a background correction wavelength of 540 nm. Area under the curve (AUC) was calculated individually for each animal at each timepoint by integration of the entire ELISA titer curve which allows for the capture of both to quantitative and qualitative aspects of ELISA.

### Immunoglobulin DNA and repertoire sequencing (AIRR-Seq) sample preparation

For immunoglobulin sequencing (AIRR-Seq), PBMCs were isolated from 15–20 ml of blood from each animal. For immunoglobulin repertoire sequencing. Approximately 5 M PBMCs were lysed in QIAGEN RLT buffer + 1% BME and stored at −80 C until RNA was purified using QIAGEN RNeasy kits. For sequencing of the immunoglobulin DNA loci, 5 M PBMCs were snap frozen and stored at −80 C.

### IGHD3–41 Genotyping

BG18-like type I BCRs utilize the RM D gene *IGHD3–41*^25^. In both humans and RMs, there is extensive genetic diversity within the immunoglobulin (IG) gene loci, including coding and non-coding single nucleotide variants^30,31,38,52–55^. To date, >800 IG alleles have been characterized in RMs, including multiple alleles at the IGHD gene, *IGHD3–41*^30,38^. The coding sequence of IGH genes is known to be important for antigen binding and development of specific responses to pathogens^56^. For BG18, the D gene is an important part of the paratope^12,25^.

To genotype IGHD3–3 (“IGHD3.41”), we utilized a custom genomic IG targeted long-read Pacific Biosciences single molecule real-time (SMRT) sequencing method (KAPA HyperExplore, Roche), following previously published protocols^25,54,61,85^ [1–4]. Briefly, genomic DNA was isolated from peripheral blood mononuclear cells (PBMCs) collected from each animal using the DNeasy kit (Qiagen). DNA (~2.5 ug) was sheared with g-tubes (Covaris); size selected >5–6 Kb (Blue Pippin/Pippin HT, Sage Science); End Repaired and A-tailed (KAPA sequencing library protocol, Roche); and finally, sample-specific sequence barcodes and universal primers were ligated. Initial PCR amplification (8–9 cycles) was conducted using PrimeSTAR GXL polymerase (Takara), followed by size-selection and purification of amplified fragments 0.7X AMPure PB beads (Pacific Biosciences). Target-enrichment was performed by hybridization of custom IGH/K/L locus-specific oligonucleotide probes (KAPA HyperExplore, Roche). Oligo-bound fragments were recovered using streptavidin beads (Roche), and additional PCR amplification (16–18 cycles) were performed using PrimeSTAR GXL (Takara). Long-read sequencing libraries were prepared using the SMRTbell Express Template Preparation Kit 2.0 (Pacific Biosciences). Resulting libraries were sequenced on the Sequel IIe system.

HiFi reads for each animal were mapped to the RheMac10 reference genome (Mmul_10) using minimap2^86^. Phased single nucleotide variants representing two distinct alleles were resolved from HiFi reads spanning the IGHD3–3 gene (chr7: 168251081–168251167). At least 10 representative HiFi reads were required to include a given allele in the genotype of an animal.

All animals carried *IGHD3–41* in their genome and were determined to be either homozygous or heterozygous for the alleles IGHD3–41*01 and IGHD3–41*01_S8240 (Table S3). Both of these alleles are capable of making BG18 type I responses using reading frame 1 or 3 (Table S3).

### AIRR-Seq library preparation:

The library preparation method was based on the protocol provided by Dr. Daniel Douek, NIAID/VRC^87,88^ and the IgM constant region primer was based on Corcoran et al, 2016^89^ and were described previously in Steichen et al, 2024^25^. RNA was extracted from sorted naive B cells in 350μL QIAGEN RLT buffer using the RNeasy Micro-DNase Digest protocol (QIAGEN) on QIAcube automation platforms (Valencia, CA). Clontech SMARTer cDNA template switching was used to perform the reverse transcription step. 8μL of RNA was mixed with 1 μL of 12μM 5’ CDS oligo(dT) primer for 3 seconds and incubated for 3 minutes at 72°C followed by at least 1 minute at 4°C. 8.5 μL of master mix comprising of 3.5 μL of 5x RT Buffer (250 mM Tris-HCl (pH 8.3), 375 mM KCl, 30 mM MgCl2), 1 μL Dithiothreitol, DTT (20 mM),1 μL dNTP Mix (10 mM), 1 μL RNAse Out (40U/μL), 1 μL SMARTer II A Oligo (12 μM), 1 μL Superscript II RT (200U/μL) was added to each reaction. After sealing and spinning the plates briefly, they were incubated for 90 minutes at 42°C and 10 minutes at 70°C. AMPure XP beads were used for purification of the first strand cDNA. For amplification of the rearranged BCR sequences, 19.3 μL of cDNA was mixed with 30.7 μL of master mix (25 μL of 2x KAPA Real-Time Library Amplification Kit (catalog# KK2702), 0.7 μL of 5PIIA (12 μM): forward primer and 5 μL of IgM primer (2 μM): reverse primer). The plates were sealed, vortexed for 5 seconds and centrifuged at 2000 RCF for 1 minute. Real-time PCR machine was used to visualize the amplification and the reaction was terminated in the exponential phase. The amplified BCRs were purified using the AMPure XP beads. Two more rounds of PCR amplification were performed for addition of barcodes and adapters. In the first round, 46 μL of master mix (25 μL of KAPA HotStart ReadyMix (catalog# KK2602), 2.5 μL of SYBR Green 1:10K and 18.5 μL of Nuclease-free water), 1 μL of P5_Seq BC_XX 5PIIA (10 μM), 1 μL of P7_i7_XX IgM (10 μM) and 2 μL of 1:10 diluted amplified rearranged BCR from previous PCR were mixed, sealed, vortexed and centrifuged. Real-time PCR amplification was performed and the resulting library was purified with AMPure XP beads. The last round of PCR amplification was performed for addition of the P5_Graft P5_Seq. Real-time PCR was carried out by mixing 44 μL of master mix ( 25 μL of KAPA HotStart ReadyMix (catalog# KK2602), 1 μL of P5_Graft P5_seq (10 μM): forward primer and 18 μL of Nuclease-free water), 1 μL of P7_i7_XX IgM (10 μM) (reverse primer) and 5 μL of amplified library from previous PCR followed by a final round of purification with AMPure XP beads. Agilent Bioanalyzer was used to assess the quality of the libraries and after pooling the libraries were sequenced on an Illumina MiSeq as 309 base paired-end runs. Primers used for AIRR-Seq library preparation and sequencing are listed below.

**Table:** 

CDS Oligo dT	TTTTTTTTTTTTTTTTTTTTTTTTTVN
SMARTer II A	AAGCAGTGGTATCAACGCAGAGTACATrGrGrG
5PIIA	AAGCAGTGGTATCAACGCAGAGT
P5_Graft_P5_Seq	AATGATACGGCGACCACCGAGATCTACAC TCTTTCCCTA**CACGACGCTCTTCCGATCT**
P5_Seq BC_XX 5PIIA	CACGACGCTCTTCCGATCT (5’ barcode) AAGCAGTGGTATCAACGCAGAGT
P7 i7_XX IgM	CAAGCAGAAGACGGCATACGAGAT (3’ barcode) GGGGCATTCTCACAGGAGACGAGGGGGAAAAG
RhIgM	GGGGCATTCTCACAGGAGACGAGGGGGAAAAG
RhIgM Seq	GGGGCATTCTCACAGGAGACGAGGGGGAAAAG
Index RhIgM	CTTTTCCCCCTCGTCTCCTGTGAGAATGCCCC

### AIRR-Seq data analysis:

Fastq files were demultiplexed using a custom script that searches for an exact match and trims the 5’ barcode followed by the 5’ RACE primer sequence with the corresponding number of upstream random nucleotides. This script uses seqtk v1.2 {https://github.com/lh3/seqtk} for extracting reads. The resulting demultiplexed files were processed by the Immcantation pipeline (docker container v4.4.0)^90,91^. The reads were assembled using the AssemblePairs module followed by filtering with the FilterSeq module with -q set to 20. The ParseHeaders module was used to add the sample name to the sequences. Duplicates were removed using the CollapseSeq module with the -–uf set to the 5’ random nucleotide sequence. SplitSeq was used to filter sequences with -DUPCOUNT of 2. IgBLAST v1.21.0^92^ was used to annotate the sequences using the germline database from Cirelli et al 2019^38^. The MakeDB module was used to create ChangeO format files from the IgBLAST output. The functional sequences were selected using ParseDB module to select productive sequences. The D gene usage frequencies were determined using the countGenes function from alakazam v1.3.0 package^91^.

### Activation-induced marker (AIM) and intracellular cytokine staining (ICS) assay to detect antigen-specific CD4^+^ T cells

Antigen-induced marker-based detection of antigen-specific T cells was performed similar to previously described protocols^93,94^. Cryopreserved PBMCs were thawed and washed in RPMI media containing 10% FBS, 1x penicillin/streptomycin and 1x GlutaMAX (R10 media). Cells were counted and then seeded at 1 million cells per well in a round-bottom 96-well plate. Cells were blocked with 0.5 μg/mL anti-CD40 mAb (Miltenyi Biotec) and incubated with anti-CXCR5 and CCR7 for 15 minutes at 37°C. Then, cells were stimulated for 24 hours with one of the following conditions: (1) 5 μg/mL N332-GT5 Env peptide pool “Env”; (2) 1 ng/mL staphylococcal enterotoxin B (SEB) used as a positive control; or (3) DMSO as a negative, unstimulated control plated in duplicate. N332-GT5 Env peptide pools consist of overlapping 15-mer peptides that cover the entire protein sequence and were resuspended in DMSO. An equimolar amount of DMSO is present in both the peptide pool and the unstimulated, negative control. After 24 hours of incubation, intracellular transport inhibitors – 0.25 μL/well of GolgiPlug (BD Biosciences) and 0.25 μL/well of GolgiStop (BD Biosciences) – were added to the samples along with the AIM marker antibodies (CD25, CD40L, CD69, OX40, 4–1BB) and incubated for 4 hours. After, cells were incubated for 15 min at RT with FC Block (Biolegend) and Fixable Live/Dead Blue (Invitrogen). ells were washed with FACS buffer (2% FBS in PBS) and stained with the surface antibodies for 30 minutes at 4°C. Following, cells were fixed with 4% formaldehyde and permeabilized with a saponin-based buffer and subsequently stained with the intracellular cytokine panel for 30 minutes at RT. Finally, the stained cells were washed and acquired on the Cytek Aurora (Cytek Biosciences). Antibody panels and reagents are summarized in table below.

For data analysis, antigen-specific T cells were measured as background subtracted data, where the linear averages of the DMSO background signal, calculated from duplicate wells for each sample, were deducted from the stimulated signal. A minimum threshold for DMSO background signals was set at 0.005% and the limit of quantitation (LOQ) was defined as the geometric mean of all DMSO samples. For each sample, the stimulation index (SI) was calculated as the ratio between the AIM^+^ response in the stimulated condition and the average DMSO response for the same sample. Samples with an SI lower than 2 for CD4 or 3 for CD8 T cells responses and/or with a background subtracted response lower than the LOQ were considered as non-responders. Non-responder samples were set at the baseline, which is the closest log_10_ value lower than the LOQ.

### AIM/ICS Staining Panel

**Table:** 

Antibodies/ Reagents	Clone	Source	Catalog #	Dilution
LIVE/DEAD Fixable Blue	-	Invitrogen	L23105	1/500
GolgiPlug	-	BD Biosciences	555029	1/1000
GolgiStop	-	BD Biosciences	554724	1/1000
Fc Block	-	BioLegend	422302	1/20
Mouse anti-human CD40	HB14	Miltenyi	130-094-133	1:200
Mouse anti-human CXCR5 PE-Cy7	MU5UBEE	Thermo Fisher Scientific	25-9185-42	1:100
Mouse anti-human CCR7 BV650	G043H7	BioLegend	353233	1:100
Mouse anti-human CD69 PE-Cy5	FN50	BioLegend	310908	1:250
Mouse anti-human CD137 (4-1BB) BV421	4B4-1	BioLegend	309819	1:250
Mouse anti-human CD25 BV605	BC96	BioLegend	302631	1:250
Mouse anti-human CD40L BB515	24-31	BD Biosciences	568170	1:250
Mouse anti-human CD134 (OX40) PE	L106	BD Biosciences	340420	1:250
Mouse anti-human CD8 BUV496	RPA-T8	BD Biosciences	612943	1:100
Mouse anti-human CD14 APC-Cy7	M5E2	BioLegend	301820	1:100
Mouse anti-human CD16 APC-eFluor780	eBioCB16	Thermo Fisher Scientific	47-0168-42	1:100
Mouse anti-human CD20 APC-Cy7	2H7	BioLegend	302314	1:100
Mouse anti-human CD3 BUV395	SP34-2	BD Biosciences	564117	1:100
Mouse anti-human CD4 PerCP-Cy5.5	OKT4	BioLegend	317428	1:100
Mouse anti-human PD-1 BV785	EH12.2H7	BioLegend	329929	1:100
Mouse anti-human CD45RA PE-CF594	5H9	BD Biosciences	565419	1:100
Armenian Hamster anti-ICOS BV480	C398.4A	BD Biosciences	566087	1:100
Mouse anti-human IFN-γ BV750	B27	BD Biosciences	566357	1:100
Rat anti-human IL-2 BUV737	MQ1-17H12	BD Biosciences	612836	1:200
Mouse anti-human TNF-α BV711	MAb11	BioLegend	502940	1:200
Mouse anti-human Granzyme B Alexa Fluor 700	GB11	BD Biosciences	560213	1:1000
Mouse anti-human IL-21 Alexa Fluor 647	3A3-N2.1	BD Biosciences	560493	1:200

### PBMC and FNA Flow Cytometry

Frozen PBMC and LN FNA samples were thawed in RPMI media with 10% FBS, 1% GlutaMAX, and 1% Penicillin/Streptomycin (R10). The recovered cells were then stained with the appropriate antibody panel. Fluorescent antigen probes were constructed by combining fluorophore-conjugated streptavidin and biotinylated N332-GT5 (N332-GT5 WT) and N332-GT5 epitope knock-out (N332-GT5 KO) probes in small increments across 45 minutes with an appropriate volume of PBS at room temperature (RT). Thawed cells were incubated with KO probes for twenty minutes at 4°C then incubated with WT probes for thirty minutes at 4°C. A master mix of surface staining antibodies was then added and incubated for thirty minutes at 4°C (see below for PBMC and FNA panels). Cells were prepared for acquisition for either analysis experiments on Cytek Aurora (Cytek Biosciences) or sorting experiments on BD FACSymphony S6 (BD Biosciences). For sorting experiments, 2 μg per 5 million cells of anti-human Total-Seq-C hashtag antibodies were added to each sample following the surface staining master mix during the staining protocol.

For analysis of bulk BGC and GC-TFH a threshold of 250 B cells and 500 CD4+ T cells was used, respectively. For analysis of Env-binding BGC, a threshold of 75 BGC cells was used. LODs for antigen- and epitope-specific B_mem_ and B_GC_ cells were determined by calculating the mean of 7 pre-immunizations samples.

Cumulative post-prime values were calculated for all GC flow-cytometry measures shown in Figure 2 by integrating the responses from week 3 to week 9 using area under the curve (AUC) to represent the post-prime period. This calculation allows for the integration of the kinetics of the germinal center to assess the overall magnitude of the response per animal in the post-prime period.

### PBMC Staining Panel

**Table:** 

Reagent	Clone	Catalog #	Dilution
AF647 Streptavidin	-	BioLegend (405237)	-
BV421 Streptavidin	-	BioLegend (405225)	-
PE Streptavidin	-	BioLegend (405245)	-
Viability APC-eFluor506	-	Thermo Fisher Scientific (65-0866-18)	1:500
CD8a APC-eFluor780	RPA-T8	BD Biosciences (563256)	1:100
CD16 APC-eFluor780	CB16	Thermo Fisher Scientific (47-0168-42)	1:100
CD14 APC-Cy7	M5E2	BioLegend (301820)	1:100
CD3 APC-Cy7	SP-34	BD Biosciences (557757)	1:100
CD20 BUV395	2H7	BD Biosciences (563781)	1:100
IgG BV605	G18-145	BD Biosciences (563246)	1:100
CD27 PE-Cy7	O323	Thermo Fisher Scientific (25-0279-42)	1:50
IgM PerCP-Cy5.5	G20-127	BD Biosciences (561285)	1:50
IgD AF488	polyclonal	Southern Biotech (2030-30)	1:50

### FNA Staining Panel

**Table:** 

Reagent	Clone	Catalog #	Dilution
AF647 Streptavidin	-	BioLegend (405237)	-
BV421 Streptavidin	-	BioLegend (405225)	-
PE Streptavidin	-	BioLegend (405245)	-
Viability APC-eFluor506	-	Thermo Fisher Scientific (65-0866-18)	1:500
CD4 BV711	OKT4	BioLegend (317440)	1:100
CD8a APC-eFluor780	RPA-T8	Thermo Fisher Scientific (47-0088-42)	1:100
CD16 APC-eFluor780	CB16	Thermo Fisher Scientific (47-0168-42)	1:100
CD20 AF488	2H7	BioLegend (302316)	1:100
CD38 PE-Cy5	OKT10	Produced in house	1:100
CD3 BUV395	SP-34	BD Biosciences (564117)	1:40
IgG BUV737	G18-145	BD Biosciences (612819)	1:40
IgM PerCP-Cy5.5	G20-127	BD Biosciences (561285)	1:40
PD1 BV605	EH12.2H7	BioLegend (329924)	1:20
CXCR5 PE-Cy7	Mu5UBEE	Thermo Fisher Scientific (25-9185-42)	1:20
CD71 PE-CF594	L01.1	BD Biosciences (Custom)	1:20

### Single B cell sequencing

After sorting, antigen specific PBMC and LN FNA B cells were wash with PBS and loaded onto a Chromium chip and controller following the manufacturers protocol (10X Genomics). Indexed V(D)J, Feature Barcode, and Gene Expression libraries were prepared following the 10X Genomics protocol with the Feature Barcoding Kit (10X Genomics) and sequenced on a NovaSeq 6000 (Illumina). Demultiplexing and FASTQ generation were done using Cell Ranger v7.2 (10X Genomics). VDJ reads were assembled de novo using Cell Ranger and contigs were aligned to a custom *Macaca mulatta* germline VDJ reference using IgBLAST and the Change-O 1.3.0 package from the Immcantation framework^91^. Sequences were assigned to animals using the MULTIseqDemux function in Seurat v4^95^. Inferred germline sequences (CreateGermline.py) and clones (DefineClones.py) were determined with Change-O. SHM was quantified using the observedMutations command in SHazaM package v1.2.0 comparing HC sequences to the inferred germline sequences (masking the D gene sequences). For clonal abundance and diversity, clonotypes for each animal were quantified using the countClones function in Alakazam package v1.3.0, only animals that had 5 or more clones were included in abundance and diversity calculations. Clonal richness (Chao1) was calculated using the iNEXT package v3.0.0 in R^96^ , clonal richness was only calculated for animals that had at least 1 sequence recovered at that timepoint.

### ELISPOT

The antibody-secreting cells (ASCs) in NHP bone marrow were quantified using the enzyme-linked immunosorbent spot (ELISpot) assay as described previously with slight modifications^97^. Briefly, 96-well multi-screen HTS filter plates (MSHAN4B50, Millipore) were coated for 16 hours at 4°C with either 5μg/mL of Goat Anti-Human Ig-UNLB (Southern Biotech, Cat# 2010–01) for capturing total rhesus IgG or 20μg/mL of Galanthus Nivalis Lectin (Vector laboratories, Cat# L-1240) for capturing N332 antigen. The plates were then washed four times with sterile PBS and blocked with R10 media for 2 hours in a 5% CO2 incubator at 37°C. For antigen capture, the GNL-coated plate was further incubated with 10μg/mL of N332 antigen for 2 hours at 37°C. Bone marrow (BM) mononuclear cells were harvested using Ficoll Paque Plus from 2 mL of BM aspirates as reported (Kasturi SP et al 2020, Sci Immunol). 100μl of cells (5×106 cells/mL) were added to the wells in the first row in duplicates (~333K cells), and serially diluted three-fold, and incubated overnight for ~16 hours in a 5% CO2 incubator at 37°C. Following incubation, the plates were washed four times with PBS followed by four washes with PBS containing 0.05% Tween 20 (PBS-T). Goat Anti-Human IgG-biotin (Southern Biotech, Cat# 2045–08) was added at a dilution of 1:500 and incubated for two hours at room temperature. Plates were washed with PBS followed by PBS-T four times each and Avidin D-HRP (Vector Labs) was added at a dilution of 1:1000 and incubated for 1 hour at room temperature. Finally, plates were washed four times in PBS-T followed by four washes with PBS, and spots were developed for 5 minutes using a filtered 3-amino 9-ethylcarbazole (AEC) substrate (0.3 mg/mL AEC in 0.1 M sodium acetate buffer, pH 5.0, with a 1:1000 dilution of 3% hydrogen peroxide). The reaction was stopped by washing the plates with running tap water, and air-dried plates before imaging using the Immunospot CTL counter and Image Acquisition 4.5 software (Cellular Technology). Total IgG+ ASC or Env-specific ASCs/spots were counted manually using magnified images and reported as the absolute number of ASCs per million BM cells or % of antigen specific ASCs as a fraction of total ASCs.

### Antibody production

High-throughput antibody production was performed as previously described^61,98^. Briefly, antibodies were produced using the ExpiCHO Expression System (Thermo Fisher) in baffled 96 deep-well blocks (Thomas Scientific) according to manufacturer’s instructions, with the following adjustments: the cell density was increased to 10^7^ viable cells/mL, cell culture volumes were increased to 1 mL/well, ExpiFectamine Reagent (Thermo Fisher) volume was increased to 6 μL/well, and incubator shake speed was set to 1300 RPM. After 7 days incubation, supernatants were supplemented with 40 μL 1M Tris, pH 8.0, harvested by centrifugation and purified using Protein A HP MultiTrap plates (GEHealthcare) per manufacturer’s instructions. The 22 BG18 type I BCRs made as antibodies were selected at random to represent 20% of all sequences from each group (see Table S2). The 8 BCRs from Group 1 represent 8 unique clones while the 14 BCRs from group 2 represent 11 unique clones. The four BG18 type I mAbs with the highest affinity were further tested for neutralization (see Table S2).

### Antibody binding affinities

We measured kinetics and affinity of antibody-antigen interactions on Carterra LSA using CMDP Sensor Chip (Carterra). This chip type has lower ligand capacity and excellent diffusion characteristics. It is recommended for use when reducing avidity is necessary. 1x HBS-EP+ pH 7.4 was our running buffer (20x stock from Teknova, Cat. No H8022) supplemented with BSA at 1mg/ml. We followed Carterra software instructions to prepare chip surface for ligands capture. In a typical experiment about 500–700 RU of capture antibody (SouthernBiothech Cat no 2047–01) in 10 mM Sodium Acetate pH 4.5 was amine coupled. The critical detail here was the concentration range of the amine coupling reagents and capture antibody. We used N-Hydroxysuccinimide (NHS) and 1-Ethyl-3-(3-dimethylaminopropyl) carbodiimidehydrochloride (EDC) from Amine Coupling Kit (GE order code BR-1000–50). As per kit instruction 22–0510-62 AG the NHS and EDC should be reconstituted in 10 ml of water each to give 11.5 mg/ml and 75 mg/ml respectively. However, the highest coupling levels of capture antibody were achieved by using 10 times diluted NHS and EDC during surface preparation runs. Thus, in our runs the concentrations of NHS and EDC were 1.15 mg/ml and 7.5 mg/ml. The activation time was reduced to 1min. The concentrated stocks of NHS and EDC could be stored frozen in −20C for up to 2 months without noticeable loss of activity. The SouthernBiotech capture antibody was used at concentration 25ug/ml with 10 minutes contact time. Phosphoric Acid 1.7% was our regeneration solution with 60 seconds contact time and injected three times per each cycle. Solution concentration of ligands was around 1 ug/ml and contact time was 5 min. Raw sensograms were analyzed using Kinetics software (Carterra), interspot and blank double referencing, Langmuir model. Analyte concentrations were quantified on NanoDrop 2000c Spectrophotometer using Absorption signal at 280 nm. For best results analyte samples should be buffer exchanged into the running buffer using dialysis. We typically cover broad range of affinities in our runs and the best referencing practices are different depending on how fast the off-rate is for particular ligand. For fast off-rate (faster than 9e-3 1/s) we use automated batch referencing that includes overlay y-aline and higher analyte concentrations. For slow off-rates (9e-3 1/s or less) we use manual process referencing that includes serial y-align and lower analyte concentrations. After automated data analysis by Kinetics software, we also did additional filtering to remove datasets with highest response signals smaller than signals from negative controls. This additional filtering could be performed automatically using R-script.

### TZM-bl pseudovirus neutralization assay

Pseudoviruses were produced in HEK293T cells (RRID:CVCL_0063) co-transfected using FuGENE 6 (Promega, cat# E2691) with pseudovirus Env-expressing plasmid and Env-deficient backbone plasmid (PSG3DEnv). Pseudoviruses were harvested 72 hours post-transfection, sterile filtered (0.45 μm), and concentrated (EMD Millipore, Cat# UFC905024). Equal volumes of serially diluted monoclonal antibodies at appropriate concentrations were incubated with HIV pseudovirus in round bottom 96-well plate (Costar Cat# 3788) at 37°C for 1 hour. TZM-bl cells were seeded 24 hours prior at 100,000 cells/ml in 50ul/well of half-area 96-well plates (Costar Cat # 3688). Plates were removed from the 37°C incubator, culture media aspirated and 25 μl of mix of mAb +PSV (with or without DEAE-Dextran (5 μg/ml final concentration) were added to each well and incubated at 37°C for 24 hours in a humidified atmosphere of 5% CO_2_. After 24 hours incubation, 75 μl of culture media was added. Plates were incubated in same conditions for additional 48 hours. After 48 hours (total 72 hours), culture media was removed, and cells were lysed with 45 μL/well 1x Luciferase Culture Lysis buffer (Promega Cat# E1531) for 20 min at RT. Neutralization was measured by adding 30 μL luciferase reagent/well (Promega, Cat #E1501) and measuring luminescence. IC_50_ was calculated using a nonlinear regression curve fit, sigmoidal, 4PL equation constrained from 0–100% in GraphPad Prism 9.3.1. IC_50_is reported as the mean IC_50_ ± SD of two biological replicates.

### Statistical Analysis

Statistical tests were carried out using GraphPad Prism 10. For most graphs unpaired two-tailed Mann-Whitney tests were used for the following comparisons (i) G1 vs 2, G1 vs 3, G1 vs 4, G1 vs 5, G1 vs 6, (ii) G2 vs 5, G2 vs 6, (iii) G4 vs 5, (iv) G5 vs 6. Only (i) tests were done for the BG18 sequencing frequency graphs except in Figure 3A were done using Fishers exact tests comparing each group to G1 individually. P-values are listed if <0.1 or reported as NS if >0.1. Additional tests were carried out in Figure 4 comparing each group to the pre-immunization samples using a Kruskal-Wallis test without correction. Those p-values are represented by the following scale: NS > 0.05, * <0.05 ,** <0.01, *** <0.001, **** <0.0001. All p-values reported in Figure 7 are from Mann-Whitney tests.

## Supplementary Material

1

## Figures and Tables

**Key resources table:** 

REAGENT or RESOURCE	SOURCE	IDENTIFIER
Antibodies		
CD4-BV711	BioLegend	RM4-5 clone
B220-PE-Cy7	BioLegend	RA3-6B2 clone
CD38-FITC	BioLegend	90 clone
CXCR5-PE	BioLegend	L138D7 clone
PD-1-BV421	BioLegend	29F.1A12 clone
GL7-PerCP-Cy5.5	BioLegend	GL7 clone
Zombie Aqua Fixable Viability Kit	BioLegend	
Live/Dead Fixable Blue	Invitrogen	Cat#L23105
GolgiPlug	BD Biosciences	Cat#555029
GolgiStop	BD Biosciences	Cat#554724
Fc Block	BioLegend	Cat#422302
Mouse anti-human CD40	Miltenyi	Cat#130-094-133, Clone HB14
Mouse anti-human CXCR5 PE-Cy7	Thermo Fisher Scientific	Cat#25-9185-42, Clone MU5UBEE
Mouse anti-human CCR7 BV650	BioLegend	Cat#353233, Clone G043H7
Mouse anti-human CD69 PE-Cy5	BioLegend	Cat#310908, Clone FN50
Mouse anti-human CD137 (4-1BB) BV421	BioLegend	Cat#309819, Clone 4B4-1
Mouse anti-human CD25 BV605	BioLegend	Cat#302631, Clone BC96
Mouse anti-human CD40L BB515	BD Biosciences	Cat#568170, Clone 24-31
Mouse anti-human CD134 (OX40) PE	BD Biosciences	Cat#340420, Clone L106
Mouse anti-human CD8 BUV496	BD Biosciences	Cat#612943, Clone RPA-T8
Mouse anti-human CD14 APC-Cy7	BioLegend	Cat#301820, Clone M5E2
Mouse anti-human CD16 APC-eFluor780	Thermo Fisher Scientific	Cat#47-0168-42, Clone eBioCB16
Mouse anti-human CD20 APC-Cy7	BioLegend	Cat#302314, Clone 2H7
Mouse anti-human CD3 BUV395	BD Biosciences	Cat#564117, Clone SP34-2
Mouse anti-human CD4 PerCP-Cy5.5	BioLegend	Cat#317428, Clone OKT4
Mouse anti-human PD-1 BV785	BioLegend	Cat#329929, Clone EH12.2H7
Mouse anti-human CD45RA PE-CF594	BD Biosciences	Cat#565419, Clone 5H9
Armenian Hamster anti-ICOS BV480	BD Biosciences	Cat#566087, Clone C398.4A
Mouse anti-human IFN-γ BV750	BD Biosciences	Cat#566357, Clone B27
Rat anti-human IL-2 BUV737	BD Biosciences	Cat#612836, Clone MQ1-17H12
Mouse anti-human TNF-α BV711	BioLegend	Cat#502940, Clone MAb11
Mouse anti-human Granzyme B Alexa Fluor 700	BD Biosciences	Cat#560213, Clone GB11
Mouse anti-human IL-21 Alexa Fluor 647	BD Biosciences	Cat#560493, Clone 3A3-N2.1
AF647 Streptavidin	BioLegend	Cat#405237
BV421 Streptavidin	BioLegend	Cat#405225
PE Streptavidin	BioLegend	Cat#405245
Viability APC-eFluor506	Thermo Fisher Scientific	Cat#62-0866-18
CD8a APC-eFluor 780	BD Biosciences	Cat#563256, Clone RPA-T8
CD3 APC-Cy7	BD Biosciences	Cat#557757, Clone SP-34
CD20 BUV395	BD Biosciences	Cat#563781, Clone 2H7
IgG BV605	BD Biosciences	Cat#563246, Clone G18-145
CD27 PE-Cy7	Thermo Fisher Scientific	Cat#25-0279-42, Clone O323
IgM PerCP-Cy5.5	BD Biosciences	Cat#561285, Clone G20-127
IgD Alexa Fluor 488	Southern Biotech	Cat#2030-30, polyclonal
CD4 BV711	BioLegend	Cat#317440, Clone OKT4
CD20 Alexa Fluor 488	BioLegend	Cat#302316, Clone 2H7
CD38 PE-Cy5	produced in house	Clone OKT10
IgG BUV737	BD Biosciences	Cat#612819, Clone G18-145
PD1 BV605	BioLegend	Cat#329924, Clone EH12.2H7
CD71 PE-CF594	BD Biosciences	custom, Clone L01.1
Anti-rhesus IgG-HRP	BioRad	Cat#AAI42
Goat Anti-Human Ig-UNLB	Southern Biotech	Cat#2010-01
Galanthus Nivalis Lectin	Vector Laboratories	Cat#L-1240
Goat Anti-Human IgG Biotin	Southern Biotech	Cat#2045-08
Bacterial and virus strains		
BG505_V1_G T5_	Steichen et al^25^	
BG505_V1_5.4_	Steichen et al^25^	
BG505_V1_5.5_	Steichen et al^25^	
BG505_V1_5.6_	Steichen et al^25^	
BG505_T332N	Steichen et al^25^	
Biological samples		
HEK293T cells		RRID: CVCL_0063
N332-GT5 CHO cell line	This paper	ATUM, US10041077
Chemicals, peptides, and recombinant proteins		
Alhydrogel	Invivogen	vac-alu-50
Nano-alum	This study	
SMNP	Silva et al^39^	
N332-GT5	Steichen et al^25^	
N332-GT5-KO	Steichen et al^25^	
pSer-N332-GT5	This paper	
Critical commercial assays		
Leap-in transposon expression system	ATUM	US10041077
BirA biotin-protein ligase reaction kit	Avidity	BirA500
Malachite Green Phosphoprotein Phosphate Estimitation Assay kit	Thermo Scientific	
ExpiCHO Expression System	Thermo Scientific	
Chromium Next GEM Single Cell 5’ Kit v2	10X Genomics	PN-1000263
KAPA HyperExplore	Roche	
SMRTbell Express Template Preparation Kit 2.0	Pacific Biosciences	Product PN: 100-938-900
KAPA HiFi HotStart Real-time PCR Master Mix (2X)	Roche	Cat#KK2702
96-well multi-screen HTS filter plates	Millipore	Cat#MSHAN4B50
FuGENE6	Promega	Cat#E2691
Deposited data		
Sequencing from epitope-specific B cells	This paper	GEO: GSEXXX
Sequencing from IgM repertoire	This paper	GEO: GSEXXX
Sequencing from long read genomic	This paper	GEO: GSEXXX
Experimental models: Organisms/strains		
Rhesus macaque (Macaca Mulatta)	Emory National Primate Research Center	
BALB/c mice	Jackson Laboratory	
Oligonucleotides		
CDS Oligo dT: TTTTTTTTTTTTTTTTTTTTTTTTTVN	Huang et al^87^	
SMARTer II A:AAGCAGTGGTATCAACGCAGAGTACATrGrGrG	Huang et al^87^	
5PIIA: AAGCAGTGGTATCAACGCAGAGT	Huang et al^87^	
P5_Graft P5_Seq: AATGATACGGCGACCACCGAGATCTACACTCTTTCCCTA**CACGACGCTCTTCCGATCT**	Huang et al^87^	
P5_Seq BC_XX 5PIIA: CACGACGCTCTTCCGATCT (5’ barcode) AAGCAGTGGTATCAACGCAGAGT	Huang et al^87^	
P7 i7_XX IgM: CAAGCAGAAGACGGCATACGAGAT (3’ barcode) GGGGCATTCTCACAGGAGACGAGGGGGAAAAG	Huang et al^87^	
RhIgM: GGGGCATTCTCACAGGAGACGAGGGGGAAAAG	Steichen et al^25^	
RhIgM Seq: GGGGCATTCTCACAGGAGACGAGGGGGAAAAG	Steichen et al^25^	
Index RhIgM: CTTTTCCCCCTCGTCTCCTGTGAGAATGCCCC	Steichen et al^25^	
Software and algorithms		
Prism 10	GraphPad	https://www.graphpad.com/features
FlowJo 10 v10.0	FlowJo, LLC	https://www.flowjo.com/
R 4.3.2	The R Foundation	https://www.r-project.org/
CellRanger v7.2	10x Genomics	https://www.10xgenomics.com/support/software/cell-ranger/latest
Seurat v4	Hao et al^95^	
Immcantation Framework	Gupta et al^91^	
iNEXT v3.0.0	Hsieh et al^96^	
IgBLAST v1.21.0	Ye et al^92^	

## Highlights

- Rare precursor B cell recruitment is modulated by adjuvants and delivery strategies
- ED+SMNP immunization led to more bnAb somatic hypermutation than a bolus immunization
- Other adjuvants and delivery strategies only inconsistently primed BG18 type I cells
- Antibody feedback shaped the booster response to germline-targeting immunization
