# Supplementary material for: Diverse priming outcomes under conditions of very rare precursor B cells
Source: Immunity. Author manuscript; Available in PMC 2025 May 8. (PMC12060733; doi:10.1016/j.immuni.2025.03.003)
Supplement: 1 [file NIHMS2063965-supplement-1.pdf]

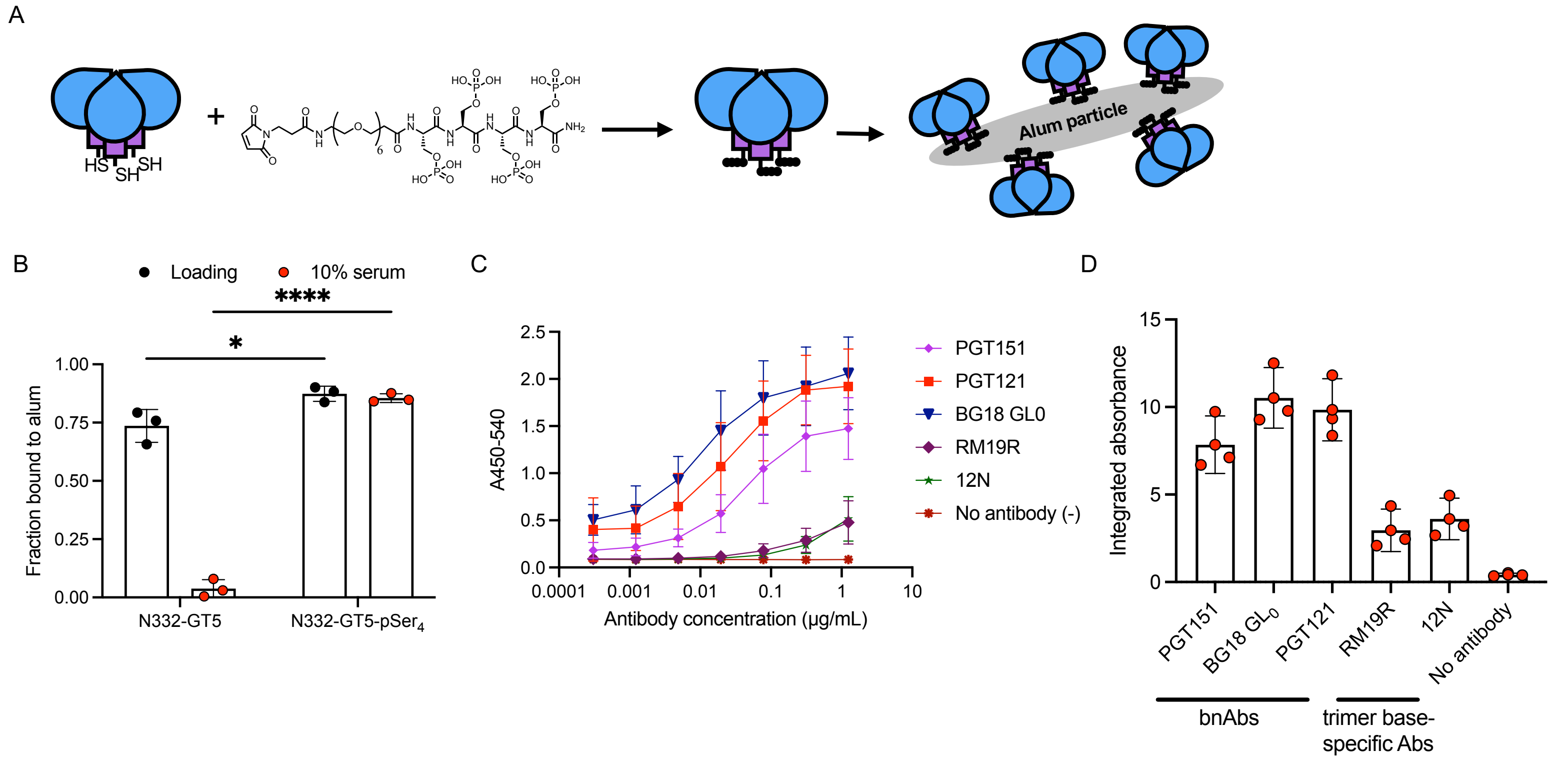

Supplementary Figure 1: pSer modification and antigenicity. Related to Figure 1.

A) Schematic of pSer peptide coupling to c-terminal Cys at the base of Env trimers and subsequent binding to aluminum hydroxide particles.

B) Fluorescently-labeled pSer-conjugated or unmodified N332-GT5 trimers were mixed with alum in TBS, and the fraction of protein bound to alum was assessed after initial 30-minute adsorption ("Loading") or after 24-hour incubation in 10% mouse serum at 37°C by fluorescence spectroscopy.

C) Antigenicity profiling ELISA assessing binding of serial dilutions of the indicated mAbs to pSer-N332-GT5 adsorbed to plate-immobilized alum.

D) Area-under-the-curve values for mAb binding vs. antibody concentration from data shown in (**C**).

Mean and SD are plotted. Statistical significance was tested using two-way ANOVA with Sidak's multiple comparisons test in (**B**). * $p < 0.05$, ****$p < 0.0001$.

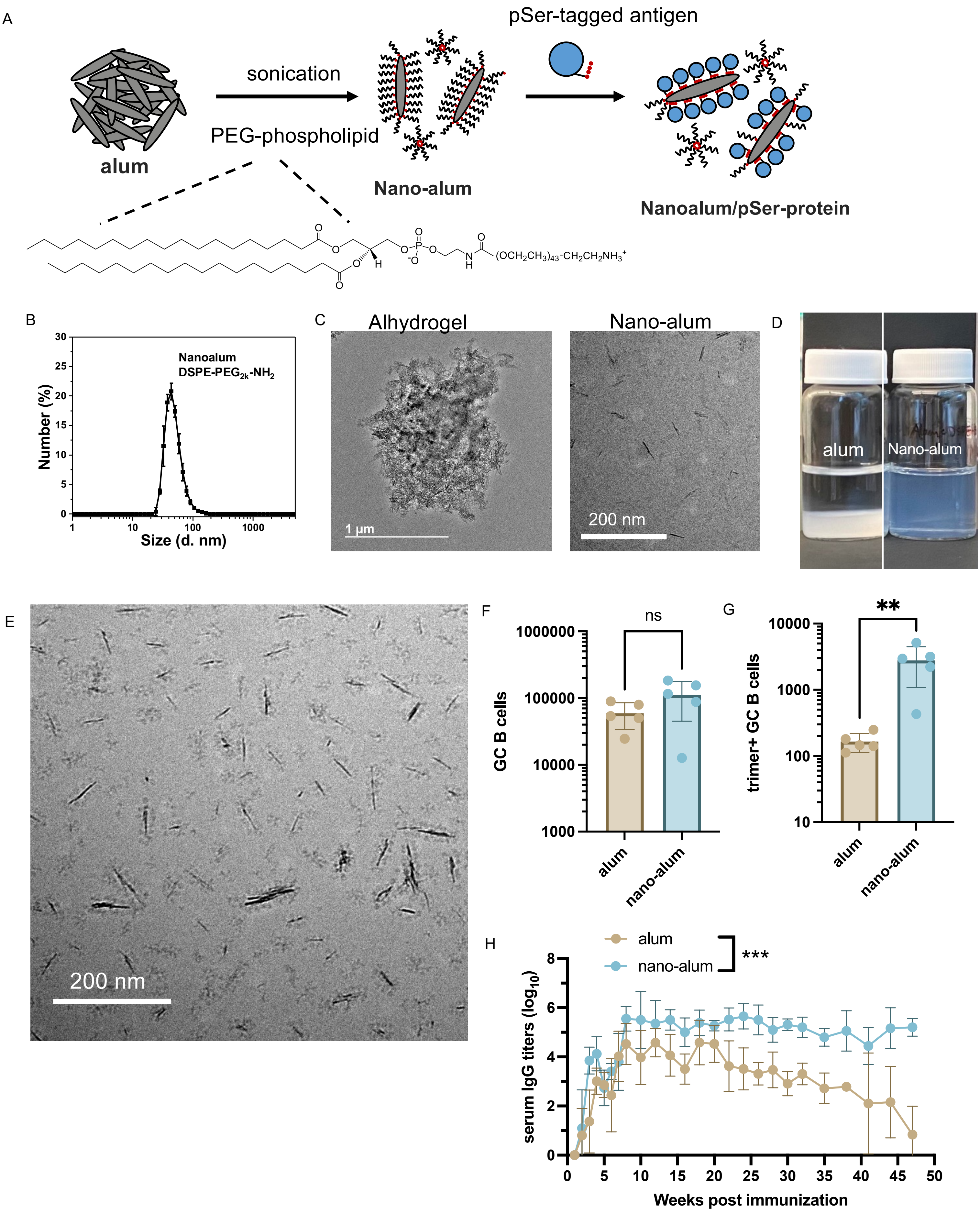
A
pSer-tagged antigen
sonication
PEG-phospholipid
alum
Nano-alum
Nanoalum/pSer-protein
(OCH2CH3)43-CH2CH2NH3+
B
Nanoalum
DSPE-PEG2k-NH2
Number (%)
Size (d. nm)
C
Alhydrogel
Nano-alum
1 μm
200 nm
D
alum
Nano-alum
E
200 nm
F
ns
GC B cells
alum
nano-alum
G
**
trimer+ GC B cells
alum
nano-alum
H
alum
nano-alum
***
serum IgG titers (log10)
Weeks post immunization

Supplementary Figure 2: Nano-alum synthesis and characterization. Related to Figure 1.
A) Schematic of nano-alum formulation and binding with pSer-trimers.
B) Number-average particle size of nano-alum determined by dynamic light scattering.
C) TEM image of neat Alhydrogel and cryoEM image of nano-alum. **D)** Photographs illustrating physical appearance of sedimented alum solution vs. stable opalescent nano-alum solution.
E) CryoEM image of pSer-trimer mixed with nano-alum showing trimers decorating individual aluminum hydroxide nanocrystals.
F-H) BALB/c mice (*n* = 5 animals/group) were immunized with 5 µg pSer-trimer adsorbed to 50 µg Alhydrogel (alum) or 50 µg nano-alum. Shown are flow cytometry analyses of total GC B cells **(F)** and trimer-specific GC B cells **(G)** on day 14 and serum IgG titers determined by ELISA over time (H). Statistical significance determined by two-tailed *t* test in **(F, G)** and two-way ANOVA in (H). ns, not significant; **, $p < 0.01$; ***, $p < 0.001$.

A

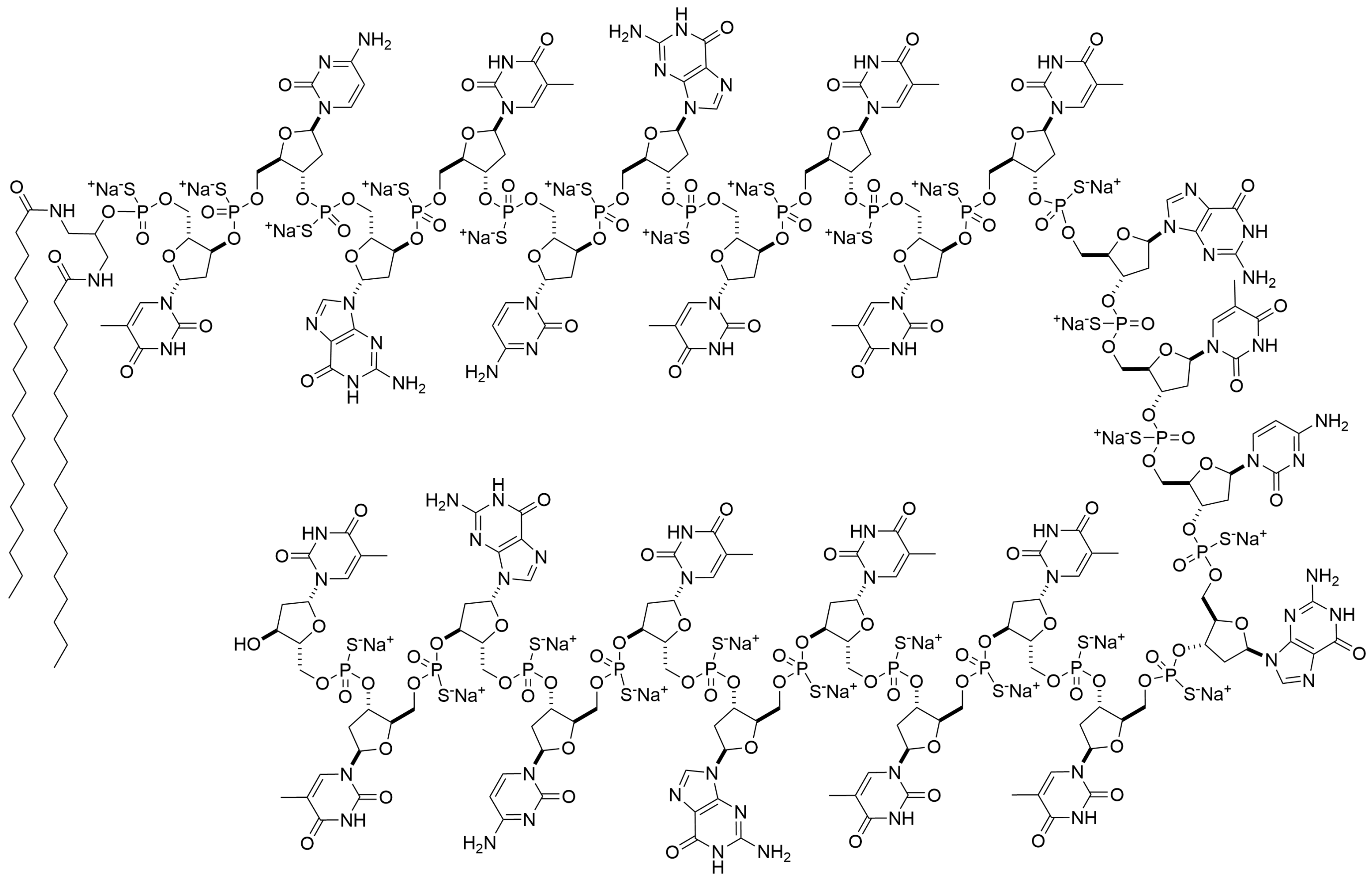

Supplementary Figure 3: Amph-CpG adjuvant. Related to Figure 1.
A) Molecular structure of Amph-CpG.

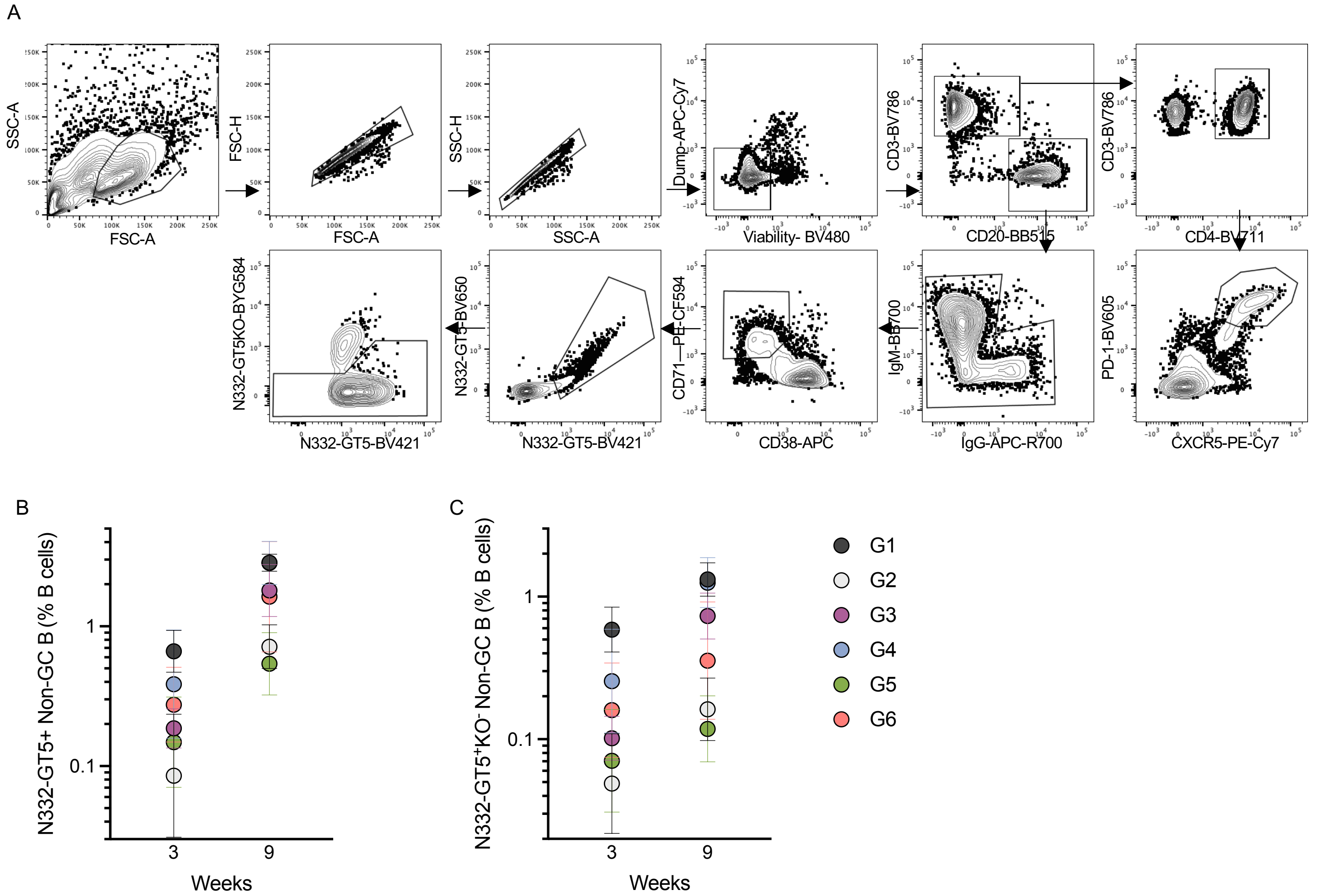

Supplementary Figure 4: Germinal center flow cytometry gating. Related to Figure 2.

A) Gating strategy for LN FNA analysis. GC-T_{FH} gating and B_{GC} gates used for quantification are shown with antigen- and epitope-specific gates. Epitope-specific B_{GC} cells were sorted.

B) Antigen-specific non-GC B cell frequency (N332-GT5-AF647$^+$N332-GT5-BV421$^+$) as a percentage of total B cells (CD20$^+$). Geometric mean and SD are shown for each group at week 3 and 9. **C)** Antigen-specific non-GC B cell frequency (N332-GT5-AF647$^+$N332-GT5-BV421$^+$N332-GT5KO-PE$^-$) as a percentage of total B cells (CD20$^+$). Geometric mean and SD are shown for each group at week 3 and 9.

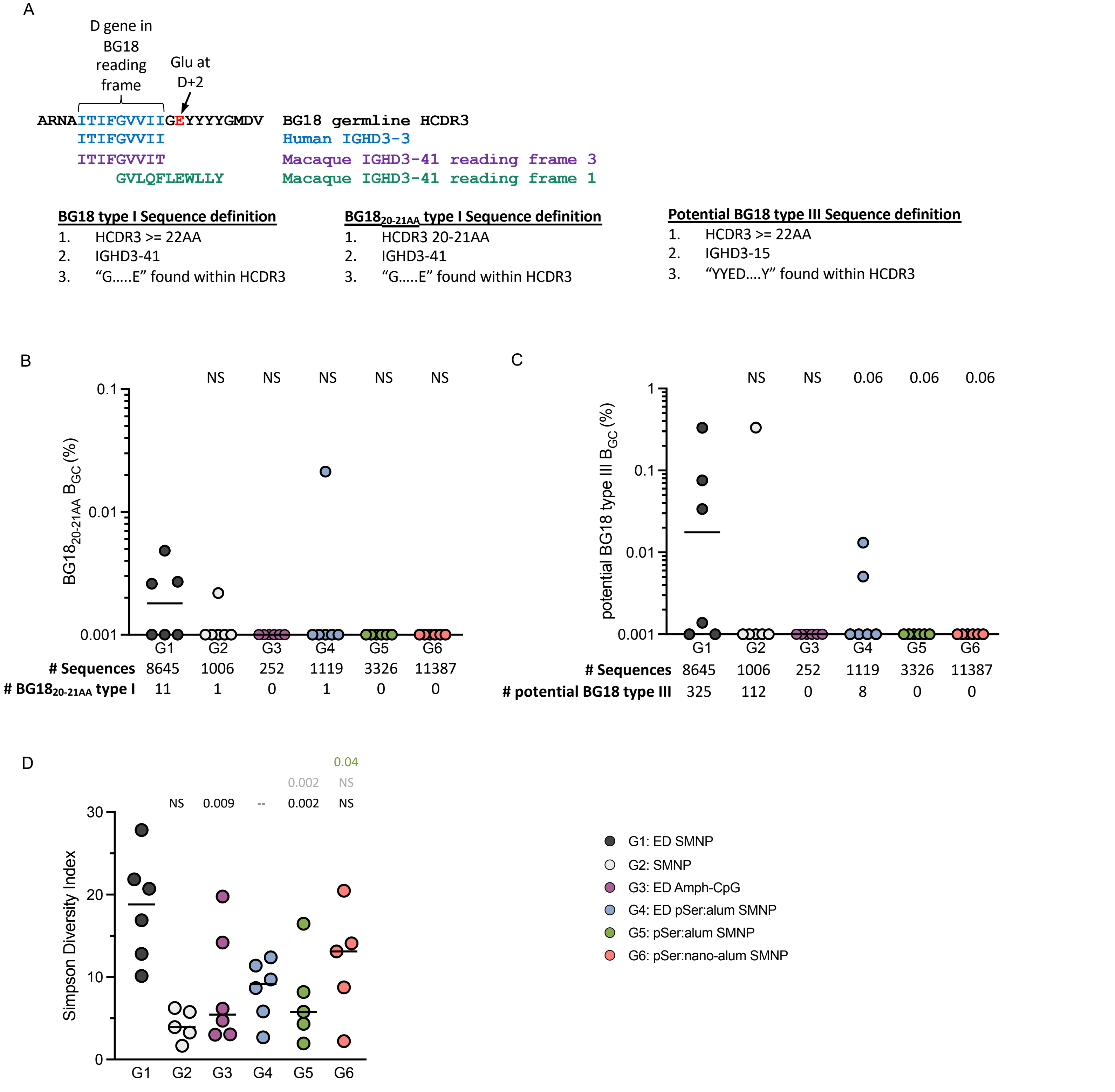

Supplementary Figure 5: BG18 sequencing. Related to Figure 3.

A) Sequence alignment of germline BG18 H-CDR3 with macaque IGHD3-41 and sequence definitions of BG18 type I, $BG18_{20\text{-}21AA}$, and potential BG18 type III BCRs.

B) Frequency of $BG18_{short}$ B_{GC} cells among total B cells, plotted per animal. Numbers below indicate total number of paired BCR sequences recovered from each group and the total number of $BG18_{short}$ BCRs recovered.

C) Frequency of potential BG18 type III B_{GC} cells among total B cells, plotted per animal. Numbers below indicate total number of paired BCR sequences recovered from each group and the total number of potential BG18 type III BCRs recovered.

D) Simpson diversity index of BCR sequences plotted per animal.

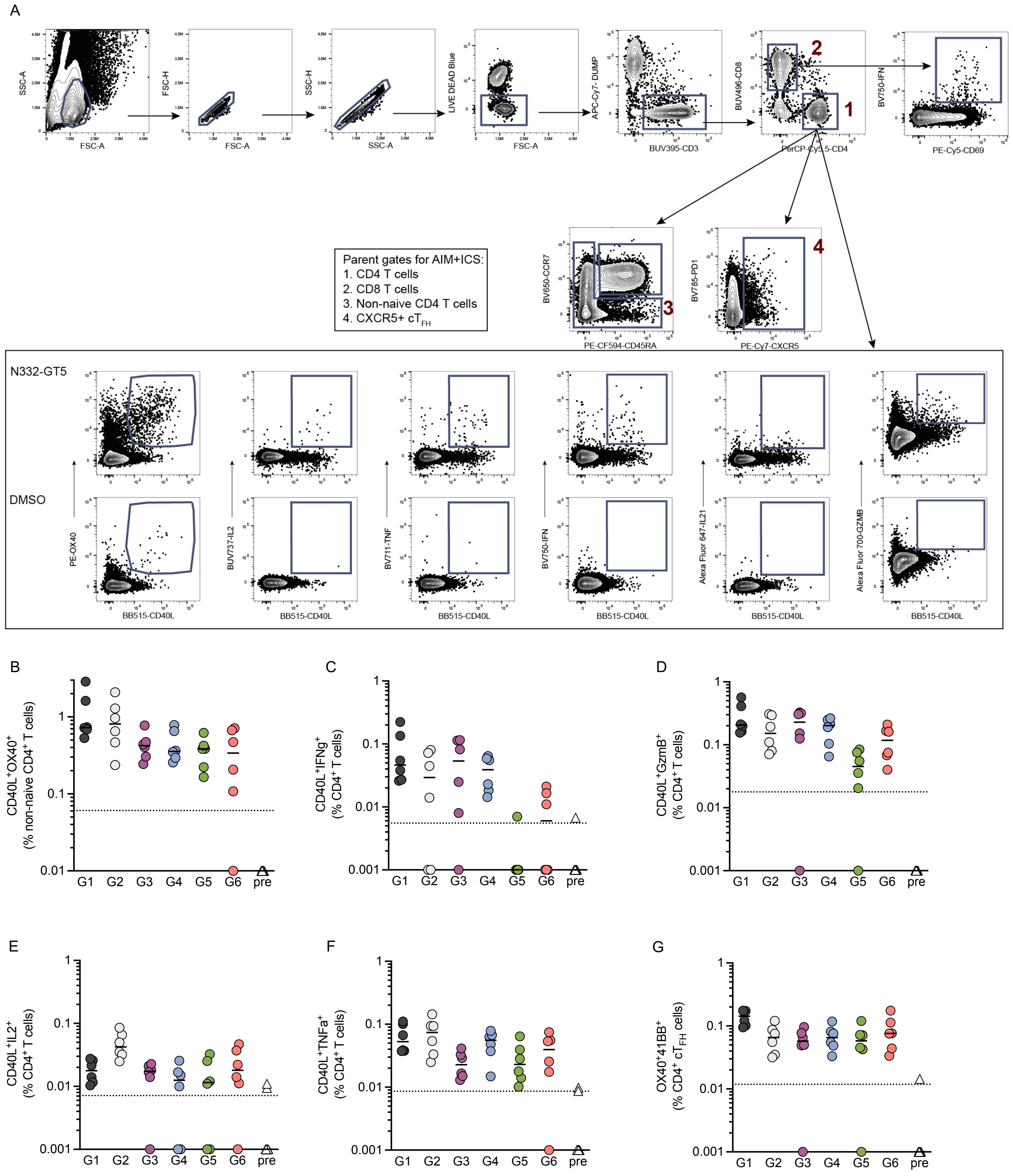
A
SSC-A
FSC-A
FSC-H
SSC-H
LIVE DEAD Blue
APC-Cy7- DUMP
BUV395-CD3
BUV496-CD8
PerCP-Cy5.5-CD4
BV750-IFN
PE-Cy5-CD69
1
2
3
4
Parent gates for AIM+ICS:
1. CD4 T cells
2. CD8 T cells
3. Non-naive CD4 T cells
4. CXCR5+ cTFH
BV650-CCR7
PE-CF594-CD45RA
BV785-PD1
PE-Cy7-CXCR5
N332-GT5
DMSO
PE-OX40
BUV737-IL2
BV711-TNF
BV750-IFN
Alexa Fluor 647-IL21
Alexa Fluor 700-GZMB
BB515-CD40L
B
CD40L+OX40+ (% non-naive CD4+ T cells)
C
CD40L+IFNg+ (% CD4+ T cells)
D
CD40L+GzmB+ (% CD4+ T cells)
E
CD40L+IL2+ (% CD4+ T cells)
F
CD40L+TNFa+ (% CD4+ T cells)
G
OX40+41BB+ (% CD4+ cTFH cells)
G1
G2
G3
G4
G5
G6
pre

Supplementary Figure 6: AIM ICS gating and data. Related to Figure 4.

A) Flow cytometry gating for AIM ICS T cell assay.

B) Frequency of AIM^+ ($CD40L^+OX40^+$) T cells out of total $CD4^+$ T cells.

C) Frequency of AIM^+ICS^+ ($CD40L^+IFN\gamma^+$) T cells out of total $CD4^+$ T cells.

D) Frequency of AIM^+ICS^+ ($CD40L^+GzmB^+$) T cells out of total $CD4^+$ T cells.

E) Frequency of AIM^+ICS^+ ($CD40L^+IL2^+$) T cells out of total $CD4^+$ T cells.

F) Frequency of AIM^+ICS^+ ($CD40L^+TNF\alpha^+$) T cells out of total $CD4^+$ T cells.

G) Frequency of AIM^+ ($OX40^+41BB^+$) cT_{FH} cells out of total $CD4^+$ cT_{FH} cells.

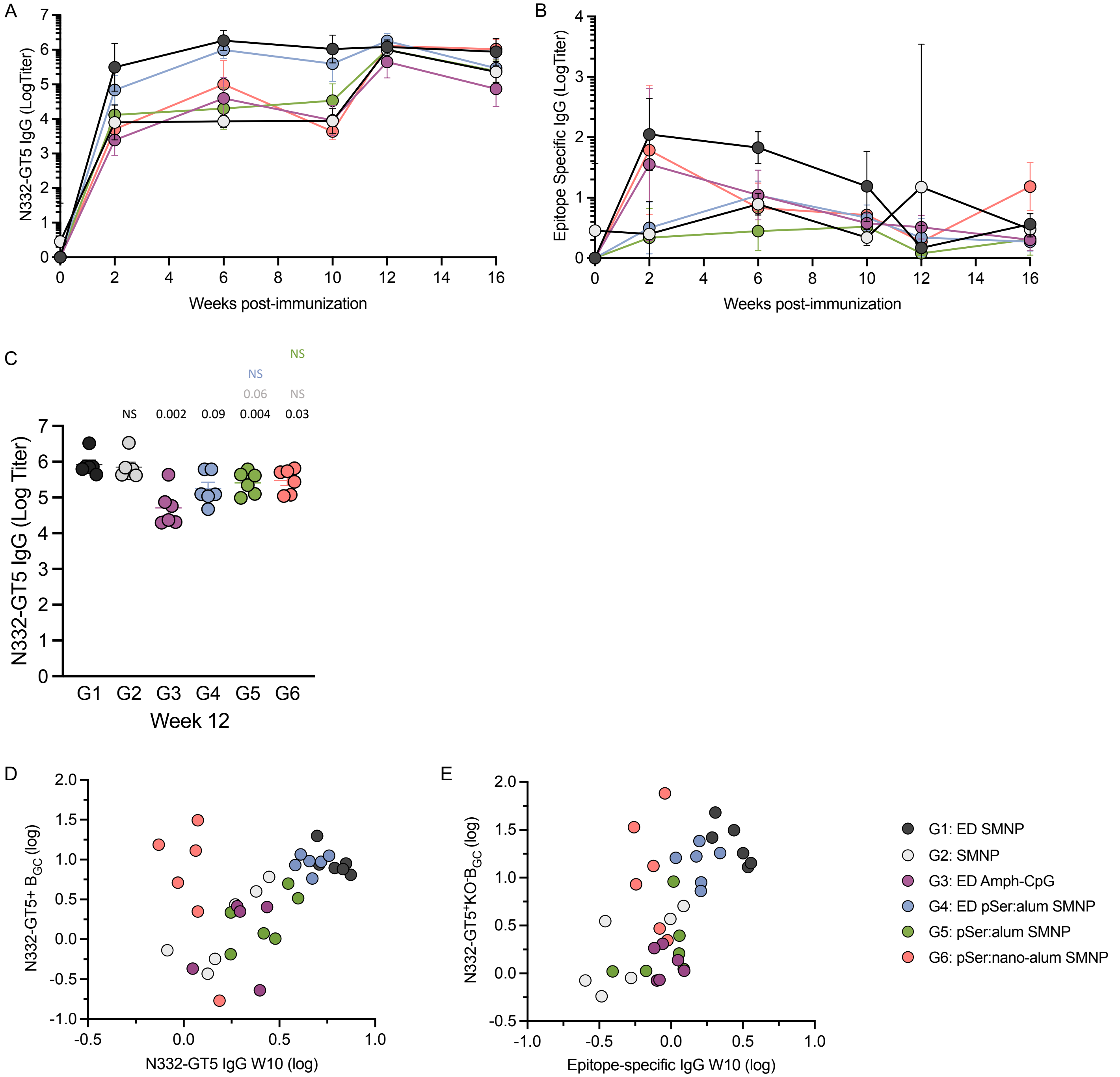

	r	p-value
**All groups**	0.41	0.015
**Excluding G6**	0.80	<0.0001

	r	p-value
**All groups**	0.57	0.0003
**Excluding G6**	0.76	<0.0001

Supplementary Figure 7: ELISA data and B_{GC} correlations. Related to Figure 4 and 6.

A) Longitudinal endpoint titer curves of total antigen-specific serum IgG measured by ELISA.

B) Longitudinal endpoint titer curves of total epitope-specific serum IgG measured by ELISA. Geometric mean and SD are plotted for each group at each time point on a Log(titer) axis. Median log10 endpoint titers G1 vs G2, 1.81 vs 0.39.

C) Endpoint titers at week 12 plotted on a per animal basis.

D) Correlation between total post-prime antigen-specific B_{GC} and week 10 antigen-specific IgG AUC. r and p-value are from pearson correlation analysis run with all groups included or excluding G6.

E) Correlation between total post-prime epitope-specific B_{GC} and week 10 epitope-specific IgG AUC. r and p-value are from pearson correlation analysis run with all groups included or excluding G6.

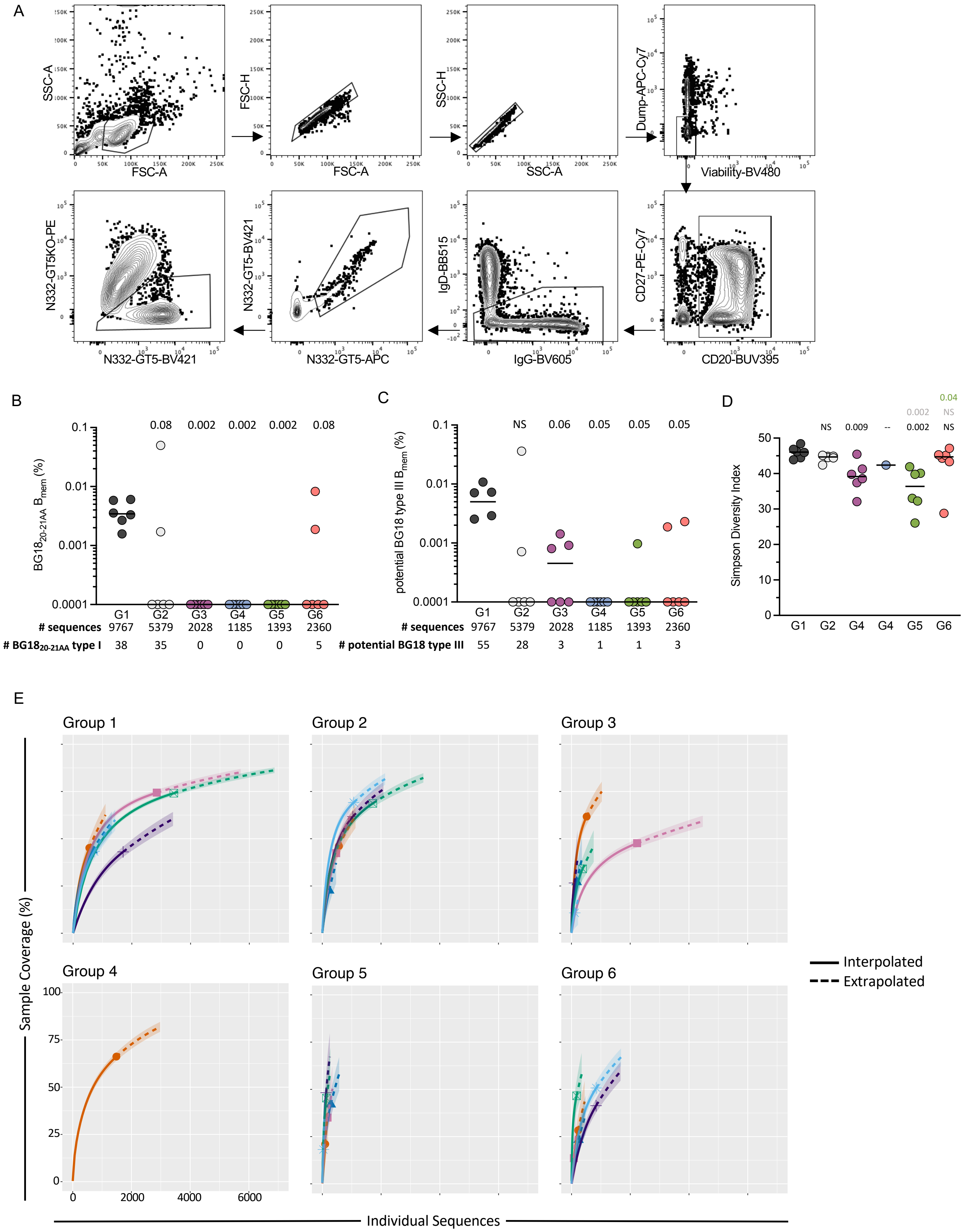
A
SSC-A
FSC-A
FSC-H
FSC-A
SSC-H
SSC-A
Dump-APC-Cy7
Viability-BV480
CD27-PE-Cy7
CD20-BUV395
IgD-BB515
IgG-BV605
N332-GT5-BV421
N332-GT5-APC
N332-GT5KO-PE
N332-GT5-BV421
B
BG18 20-21AA Bmem (%)
0.08 0.002 0.002 0.002 0.08
G1 G2 G3 G4 G5 G6
sequences 9767 5379 2028 1185 1393 2360
BG18 20-21AA type I 38 35 0 0 0 5
C
potential BG18 type III Bmem (%)
NS 0.06 0.05 0.05 0.05
G1 G2 G3 G4 G5 G6
sequences 9767 5379 2028 1185 1393 2360
potential BG18 type III 55 28 3 1 1 3
D
Simpson Diversity Index
0.04
0.002 NS
NS 0.009 -- 0.002 NS
G1 G2 G4 G4 G5 G6
E
Group 1
Group 2
Group 3
Group 4
Group 5
Group 6
Sample Coverage (%)
Individual Sequences
Interpolated
Extrapolated

Supplementary Figure 8: B_{mem} flow cytometry and sequencing. Related to Figure 5.

A) Flow cytometry gating strategy for isolating epitope-specific B_{mem} cells from PBMCs.

B) Frequency of $BG18_{short}$ B_{GC} cells among total B cells, plotted per animal. Numbers below indicate total number of paired BCR sequences recovered from each group and the total number of $BG18_{short}$ BCRs recovered.

C) Frequency of potential BG18 type III B_{GC} cells among total B cells, plotted per animal. Numbers below indicate total number of paired BCR sequences recovered from each group and the total number of potential BG18 type III BCRs recovered.

D) Simpson diversity index of BCR sequences plotted per animal. Statistical significance was tested using unpaired two-tailed Mann-Whitney tests with the p-values listed on each graph representative of the tests carried out, NS listed when p-value was >0.1.

E) iNext plots showing the results of rarefaction analysis performed on the week 12 B_{mem} sequences to determine the % sequence coverage based on the number and clonality of the recovered sequences. Each group is plotted separately with each line representing a single animal. Only animals that had more than 5 unique clones were included in the analysis.

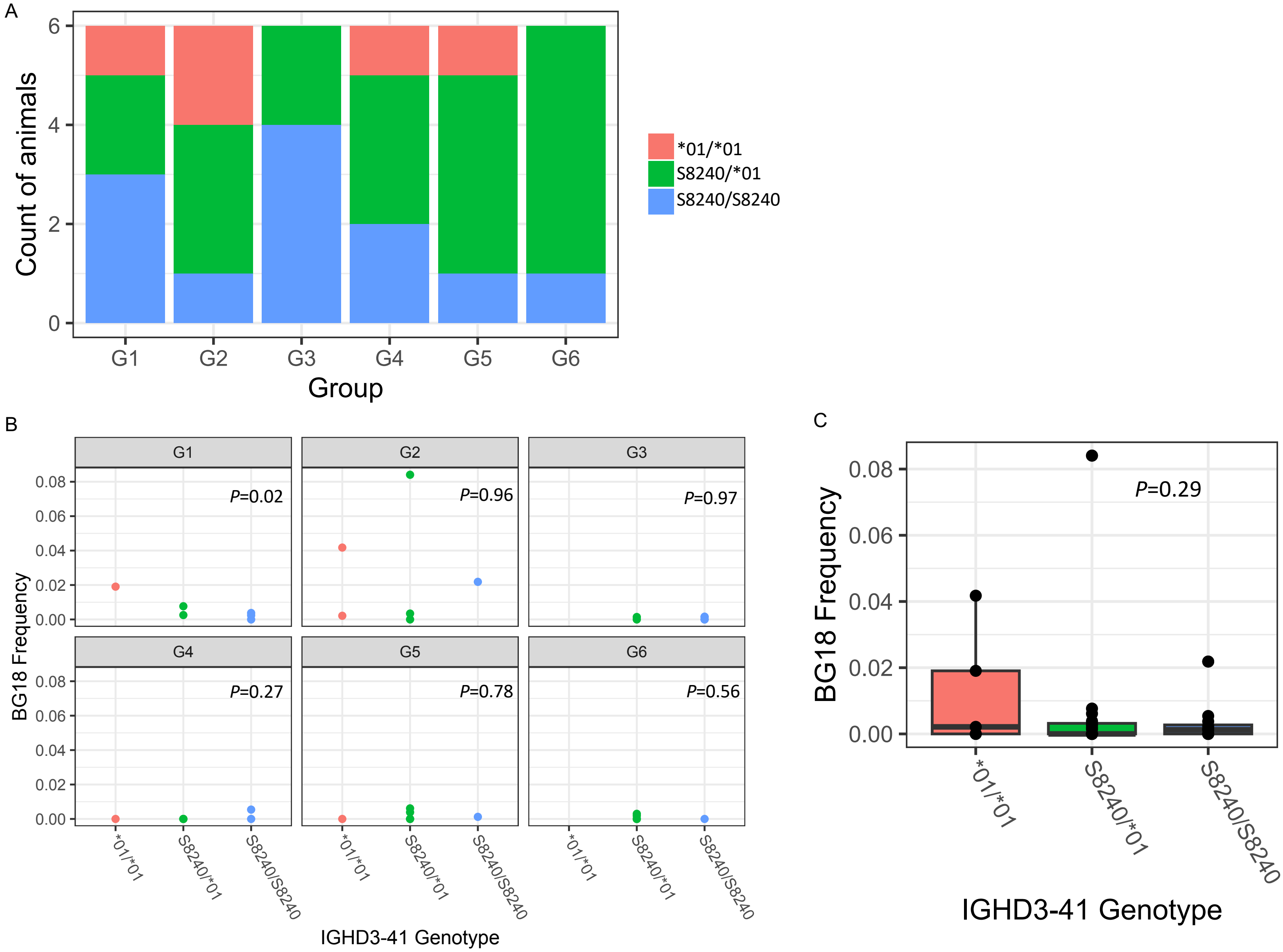

Supplementary Figure 9: IGHD3-41 Immunogenetics. Related to Figure 5.
A) IGHD3-41 genotype distribution for each animal separated by group.
B) Week 12 BG18 type I B_{mem} frequency plotted by genotype and group. Each dot represents an individual animal.
C) Week 12 BG18 type I B_{mem} frequency plotted by genotype for all 42 animals together. Statistical significance was tested using ANOVA with the p-values listed on each graph. For B) p-values represent within-group comparisons.

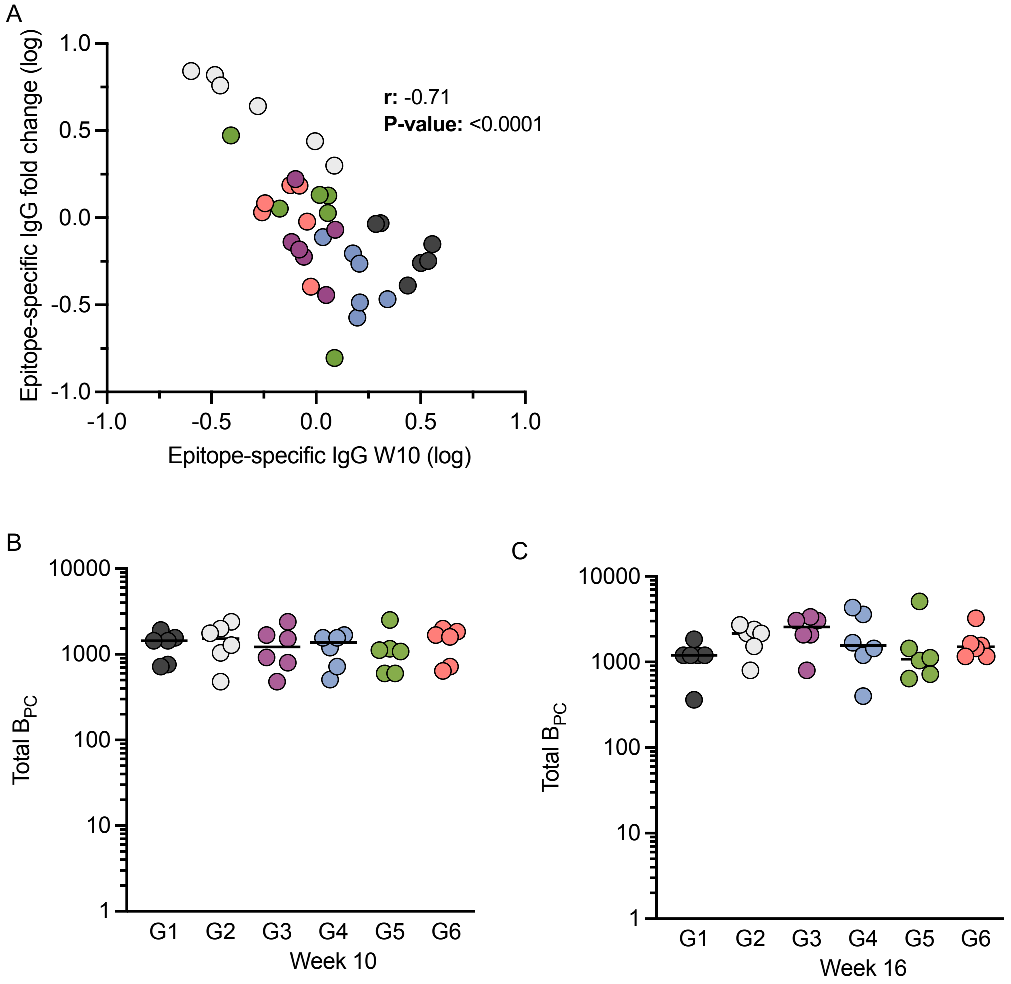

Supplementary Figure 10: Serum IgG correlations and B_{PC}. Related to Figure 6.
A) Correlation between epitope-specific fold change increase from week 10 to 12 and epitope-specific week 10 AUC. r and p-value are from pearson correlation analysis.
B-C) Total IgG^+ BM-B_{PC} measured from bone marrow aspirates by ELISpot assay at weeks 10 and 16.

A

B

C

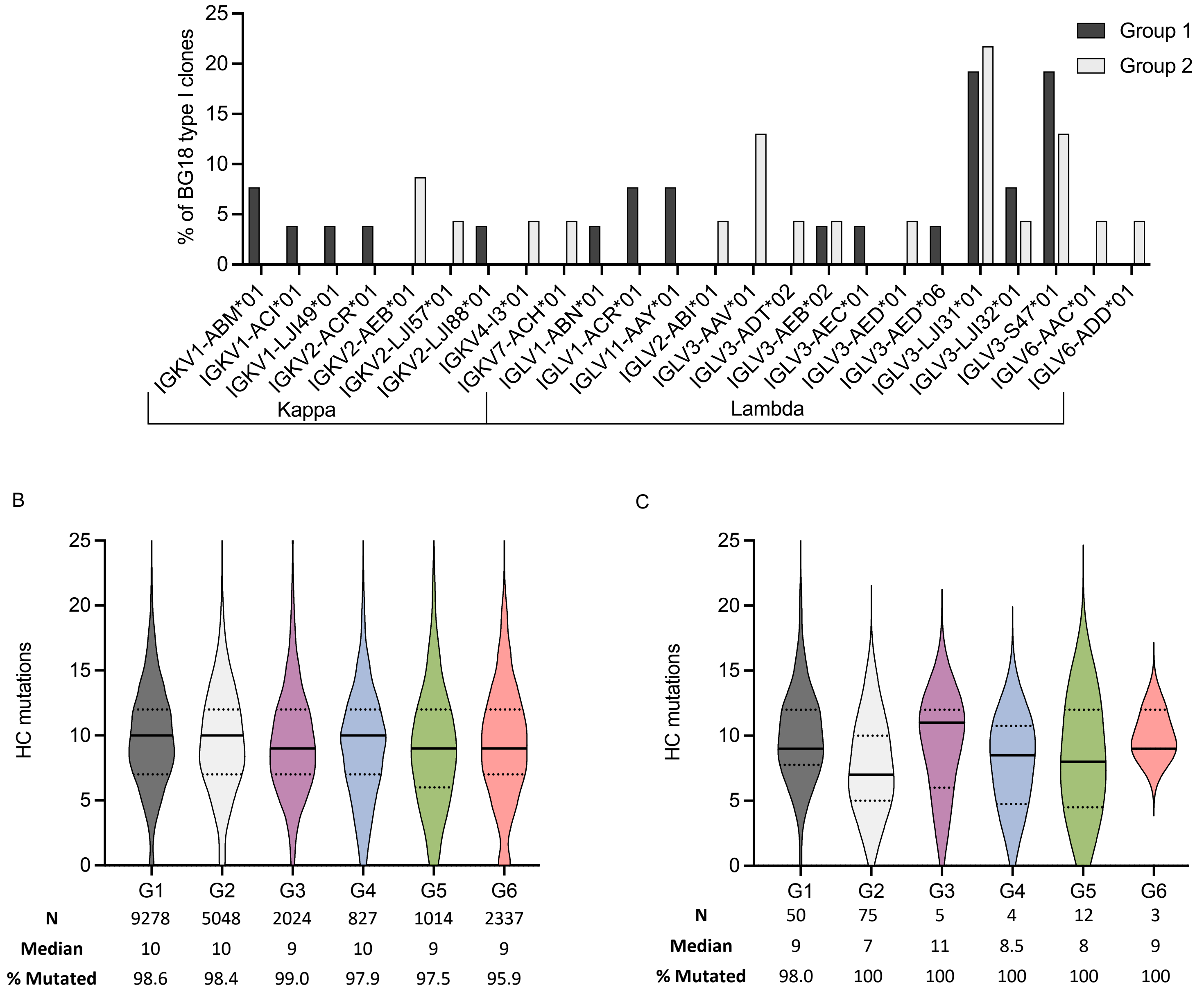

D

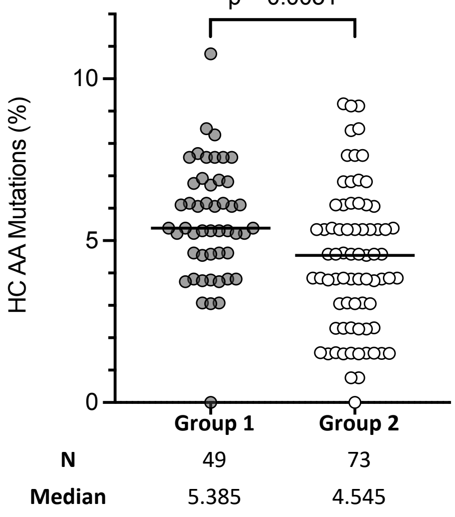

E

	PSV:	BG505_V1$_{GT5}$	BG505_V1$_{5.4}$	BG505_V1$_{5.5}$	BG505_V1$_{5.6}$	BG505-T332N	MLV
Group 1	RM_RHu20_16	0.003	0.036	>50	>50	>50	>50
	RM_RTo20_21	0.017	0.73	>50	>50	>50	>50
	RM_RUi20_19	4.4	>50	>50	>50	>50	>50
	RM_RQh20_20	0.28	>50	>50	>50	>50	>50
Group 2	RM_RTo19_08	0.13	0.39	>50	>50	>50	>50
	RM_RWc20_14	>50	>50	>50	>50	>50	>50
	RM_RCk19_09	0.10	0.16	>50	>50	>50	>50
	RM_NB18_04	0.008	0.53	>50	>50	>50	>50
	BG18	0.001	0.006	0.003	0.001	0.003	>25
	Den3	>50	>50	>50	>50	>50	>50

IC50 (μg/mL)
<0.01
0.01 - 0.1
0.1 - 1
1 - 50
>50

V1 loop

CTNYAPKLRSMMRGELKNC BG505_V1$_{GT5}$
CTNYTPKLRSMMRGELKNC BG505_V1$_{5.4}$
CTNYAPNLTSMMRGELKNC BG505_V1$_{5.5}$
CTNYTPNLTSMMRGELKNC BG505_V1$_{5.6}$

Supplementary Figure 11: Light chain usage and SHM across groups. Related to Figure 7.
A) Graph of the distribution of IGHK and IGHL genes used by BG18 type I clones in groups 1 and 2.
B) Heavy chain nucleotide mutations plotted for each group of total week 12 B_{mem} cells. Number of sequences, median mutation count, and percentage of all cells with 1 or more mutations are listed below graphs.
C) Heavy chain nucleotide mutations plotted for each group of week 12 BG18 type I B_{mem}. Number of sequences, median mutation count, and percentage of all cells with 1 or more mutations are listed below graphs.
D) Heavy chain amino acid mutation percentage for all BG18 type I B_{mem} cells from groups 1 and 2. Median mutation percentage listed below.
E) High affinity BG18 type I mAbs from 8 animals (four from each group) were evaluated in a TZM-bl assay for their ability to neutralize BG505-T332N and 4 sensitive viruses derived from BG505 but with modified V1 loops based on N332-GT5. BG18 is a positive control mAb and Den3 is a negative control mAb.

Table S1. Week 3 BG18 type I B_{GC} sequences

NHP	Clone	VH	D	JH	HCDR3	HCDR3 length	HC nucleotide mutations	VL/VK	JL/JK	LCDR3	LC nucleotide mutations
RHu20	18604	IGHV3-52*01	IGHD3-41*01	IGHJ5-2*01,IGHJ5-5*01	CARNGITIFGLVILGEFYNSLDVW	22	1	IGLV3-AEC*01	IGLJ3*01	CQSADSSGNHPVF	2
	20527	IGHV1-NL_1*01_S2052	IGHD3-41*01	IGHJ6*01,IGHJ6-6*01	CARDGITIFGLVIFGESDYYGLDSW	23	0	IGLV3-S47*01_S0001	IGLJ3*01	CQVWDISSDHPVF	1
		IGHV1-NL_1*01_S2052	IGHD3-41*01	IGHJ6*01,IGHJ6-6*01	CARDVITIFGLVILGESDYYGLDSW	23	3	IGLV3-S47*01_S0001	IGLJ3*01	CQVWDISSDHPVF	1
		IGHV1-NL_1*01_S2052	IGHD3-41*01	IGHJ6*01,IGHJ6-6*01	CARDGITIFGLVIFGESDYYGLDSW	23	0	IGLV3-S47*01_S0001	IGLJ3*01	CQVWDISSDHPVF	3
		IGHV1-NL_1*01_S2052	IGHD3-41*01	IGHJ6*01,IGHJ6-6*01	CAGDGFTIFGLVILGESDYSGLDSW	23	4	IGLV3-S47*01_S0001	IGLJ3*01	CQVWDITSDHPVF	1
		IGHV1-NL_1*01_S2052	IGHD3-41*01	IGHJ6*01,IGHJ6-6*01	CARDGITVFGLVIFGESDYYGLDSW	23	1	IGLV3-S47*01_S0001	IGLJ3*01	CQVWDISSDHPVF	0
	20528	IGHV4-149*01_S0794	IGHD3-41*01	IGHJ6*01,IGHJ6-6*01	CARDLLEIFGLVIFGEVDYYGLDSW	23	3	IGKV1-LJI49*01	IGKJ1-I1*01	CQQFNIYPRTF	1
		IGHV4-149*01_S0794	IGHD3-41*01	IGHJ6*01,IGHJ6-6*01	CARDLLEIFGLVIFGEVDYYGLDSW	23	0	IGKV1-LJI49*01	IGKJ1-I1*01	CQQRNSYPRTF	2
		IGHV4-149*01_S0794	IGHD3-41*01	IGHJ6*01,IGHJ6-6*01	CARDLLEIFGLVIFGEVDYYGLDSW	23	1	IGKV1-LJI49*01	IGKJ1-I1*01	CQQRNSYPRTF	2
		IGHV4-149*01_S0794	IGHD3-41*01	IGHJ6*01,IGHJ6-6*01	CARDLLEIFGLVIFGEVDYYGLDSW	23	0	IGKV1-LJI49*01	IGKJ1-I1*01	CQQHNTYPRTF	4
		IGHV4-149*01_S0794	IGHD3-41*01	IGHJ6*01,IGHJ6-6*01	CARDLLEIFGLVIFGEVDYYDLDSW	23	2	IGKV1-LJI49*01	IGKJ1-I1*01	CQQRNSYPRTF	1
		IGHV4-149*01_S0794	IGHD3-41*01	IGHJ6*01,IGHJ6-6*01	CARDLLEIFGLVIFGEVDYYGLDSW	23	3	IGKV1-ABZ*01	IGKJ1-I1*01	CQQRNSYPRTF	2
		IGHV4-149*01_S0794	IGHD3-41*01	IGHJ6*01,IGHJ6-6*01	SARDLLEIFGLVIFGEVDYYGLDSW	23	7	IGKV1-LJI49*01	IGKJ1-I1*01	CQQRNSYPRTF	1
RQh20	18554	IGHV4-79*01	IGHD3-41*01_S8240	IGHJ3*01,IGHJ3-2*01	CARGDITIFGVVITPESSEAFDFW	22	2	IGLV6-AAC*01,IGLV6-AAC*02	IGLJ3-S3*01	CQSYDSYNVLF	2
		IGHV4-79*01	IGHD3-41*01_S8240	IGHJ3*01,IGHJ3-2*01	CARGDITIFGVVITPESSEAFDFW	22	2	IGLV6-AAC*05,IGLV6-AAC*05_S8052	IGLJ3-S3*01	CQSYDSYNVLF	2
	20295	IGHV4-NL_21*01_S4478	IGHD3-41*01_S8240	IGHJ1*01,IGHJ1-1*01	CARGGGRIGVLQFLEWLSETEYFEFW	24	0	IGLV3-AED*06	IGLJ3*01	CQVWDSSSDHWVF	6
		IGHV4-NL_21*01_S4478	IGHD3-41*01_S8240	IGHJ1*01,IGHJ1-1*01	CARGGGRIGVLQFLEWLSETEYFEFW	24	0	IGLV3-AED*06	IGLJ3*01	CQVWDSSSDHWVF	5
	20774	IGHV4-NL_22*01_S6977	IGHD3-41*01_S8240	IGHJ6*01,IGHJ6-6*01	CAREGGSVFGVVIFTEFNYYGLDSW	23	0	IGLV3-LJI31*01	IGLJ1-S1*01	CQVWDSSSKYYIF	0
RRt20	18555	IGHV4-NL_22*01_S6977	IGHD3-41*01_S8240	IGHJ2*01,IGHJ2-I1*01	CATLGITIFGVVIFGELDWYFDLW	22	0	IGLV1-LJI69*01	IGLJ2-S2*01	CSAWDSSLSALF	1
		IGHV4-NL_22*01_S6977	IGHD3-41*01_S8240	IGHJ2*01,IGHJ2-I1*01	CATLGITIFGVVIFGELDWYFDLW	22	2	IGLV1-LJI69*01	IGLJ2-S2*01	CSAWDSSLSGLF	2
		IGHV4-NL_22*01_S6977	IGHD3-41*01_S8240	IGHJ2*01,IGHJ2-I1*01	CATLGITIFGVVIFGEVDWYFDLW	22	4	IGLV1-LJI69*01	IGLJ2-S2*01	CSAWDSSLSGLF	1
		IGHV4-NL_22*01_S6977	IGHD3-41*01_S8240	IGHJ2*01,IGHJ2-I1*01	CATLGITIFGVLIFGELDWYFDLW	22	5	IGLV1-LJI69*01	IGLJ2-S2*01	CSAWDSSLSGLF	0
		IGHV4-NL_22*01_S6977	IGHD3-41*01_S8240	IGHJ2*01,IGHJ2-I1*01	CATLGITIFGVVIFGELDWYFDLW	22	2	IGLV1-LJI69*01	IGLJ2-S2*01	CSAWDSSLSGLF	0
		IGHV4-NL_22*01_S6977	IGHD3-41*01_S8240	IGHJ2*01,IGHJ2-I1*01	CATLGITIFGVVIFGEVDWYFDLW	22	1	IGLV1-LJI69*01	IGLJ2-S2*01	CSAWDSSLSGLF	1
		IGHV4-NL_22*01_S6977	IGHD3-41*01_S8240	IGHJ2*01,IGHJ2-I1*01	CATLGITIFGVVIFGELDWYFDLW	22	0	IGLV1-LJI69*01	IGLJ2-S2*01	CSAWDSSLSGLF	0
		IGHV4-NL_22*01_S6977	IGHD3-41*01_S8240	IGHJ2*01,IGHJ2-I1*01	CATLGITIFGVVIFGELDWYFDLW	22	3	IGLV1-LJI69*01	IGLJ2-S2*01	CSVWDSSLSGLF	0
		IGHV4-NL_22*01_S6977	IGHD3-41*01_S8240	IGHJ2*01,IGHJ2-I1*01	CATLGITIFGVVIFGELDWYFDLW	22	1	IGLV1-LJI69*01	IGLJ2-S2*01	CSAWDSSLSGLF	1
		IGHV4-NL_22*01_S6977	IGHD3-41*01_S8240	IGHJ2*01,IGHJ2-I1*01	CATLGITIFGVVIFGELDWYFDLW	22	0	IGLV1-LJI69*01	IGLJ2-S2*01	CSAWDSSLSGLF	2
		IGHV4-NL_22*01_S6977	IGHD3-41*01_S8240	IGHJ2*01,IGHJ2-I1*01	CATLGITIFGVVIFGELDWYFDLW	22	0	IGLV1-LJI69*01	IGLJ2-S2*01	CSAWDSSLSGLF	1
		IGHV4-NL_22*01_S6977	IGHD3-41*01_S8240	IGHJ2*01,IGHJ2-I1*01	CTTLGITIFGVVIFGELDWYFDLW	22	0	IGLV1-LJI69*01	IGLJ2-S2*01	CSAWDSSLSGLF	1
		IGHV4-NL_22*01_S6977	IGHD3-41*01_S8240	IGHJ2*01,IGHJ2-I1*01	CATLGITIFGMVIFGEVDWYFDLW	22	2	IGLV1-LJI69*01	IGLJ2-S2*01	CSAWDSSLSGLF	1
		IGHV4-NL_22*01_S6977	IGHD3-41*01_S8240	IGHJ2*01,IGHJ2-I1*01	CATLGITIFGVVIFGELDWYFDLW	22	0	IGLV1-LJI69*01	IGLJ2-S2*01	CSTWDSSLSGLF	0
		IGHV4-NL_22*01_S6977	IGHD3-41*01_S8240	IGHJ2*01,IGHJ2*01_S5087,IGHJ2-I1*01	CATLGITIFGVVIFGELDWYFDFW	22	2	IGLV1-LJI69*01	IGLJ2-S2*01	CSAWDSSLSGLF	0
		IGHV4-NL_22*01_S6977	IGHD3-41*01_S8240	IGHJ2*01,IGHJ2-I1*01	CATLGITIFGVIIFGEVDWYFDLW	22	2	IGLV1-LJI69*01	IGLJ2-S2*01	CSVWDSSLSGLF	1

Table S2. Week 12 BG18 type I B_{mem} sequences

	NHP	Clone	VH	D	JH	HCDR3	HCDR3 length	HC nucleotide mutations	VL/VK	JL/JK	LCDR3	LC nucleotide mutations
G1	RHu20	20511	IGHV3-AFE*01_S4619	IGHD3-41*01	IGHJ6*01	CARNLRKIFGVVILGESEDYGLDSW	23	15	IGLV3-S47*01_S0001	IGLJ1-S1*01	CQLWDSNNDHYIF	10
		20512	IGHV4-AFQ*01	IGHD3-41*01	IGHJ6*01	CARIRITIFGVVALKETEENGLDSW	23	9	IGLV3-S47*01_S0001	IGLJ3-S3*01	CQVWDISSDHVLF	4
		20513	IGHV4-AFQ*01_S2532	IGHD3-41*01	IGHJ6*01	CARGHITIFGVVILGEIDYYGLDSW	23	13	IGLV3-LJI32*01	IGLJ3-S3*01	CQVWDSSSHLVLF	10
		20517	IGHV4-150*01_S9374	IGHD3-41*01	IGHJ6*01	CARESITIFGLVTFREFNYNGLDSW	23	16	IGLV3-LJI31*01_S0001	IGLJ3*01	CQVWDSSSDHRIF	5
			IGHV4-150*01_S9374	IGHD3-41*01	IGHJ6*01	CARDNIAIFGLVVFREFNYHGLDSW	23	9	IGLV3-LJI31*01_S0001	IGLJ3*01	CQVWDNNTYYQIF	5
			IGHV4-150*01_S9374	IGHD3-41*01	IGHJ6*01	CARESITIFGLVTFREFNYNGLDSW	23	8	IGLV3-LJI31*01_S0001	IGLJ3*01	CQVWDSSSDHRIF	7
			IGHV4-150*01_S9374	IGHD3-41*01	IGHJ6*01	CARDSIGIFGLLEFREFNYYGLDSW	23	13	IGLV3-LJI31*01_S0001	IGLJ3*01	CQVWDSSSDHRVF	4
		20518	IGHV3-52*01	IGHD3-41*01	IGHJ5-2*01	CARNSITIFGVVIFKEYWLHSLDVW	23	11	IGLV3-S47*01_S0001	IGLJ3*01	CQVWDISSDHPVF	6
			IGHV3-52*01	IGHD3-41*01	IGHJ5-2*01	CARTSITIFGVIIFKEYWYNSLDVW	23	7	IGLV3-S47*01_S0001	IGLJ3*01	CQVWDISSDHPVF	4
		20527	IGHV1-NL_1*01_S2052	IGHD3-41*01	IGHJ6*01	CSRERITIFGVVILGETDYHGLDSW	23	5	IGLV3-S47*01_S0001	IGLJ3*01	CQVWDISSDHPVF	5
			IGHV1-NL_1*01_S2052	IGHD3-41*01	IGHJ6*01	CSRERVTIFGVVIFGETEYHGLDSW	23	7	IGLV3-S47*01_S0001	IGLJ3*01	CQVWDNTNDHPVF	8
		20528	IGHV4-149*01_S0794	IGHD3-41*01	IGHJ6*01	CARDLLEIFGLLIFGEVENYGLDSW	23	9	IGKV1-LJI49*01	IGKJ1-I1*01	CQQCESDPRTF	9
			IGHV4-149*01_S0794	IGHD3-41*01	IGHJ6*01	CARDLLEIFGLLIFGEVENYGLDSW	23	15	IGKV1-LJI49*01	IGKJ1-I1*01	CQQCNSDPRTF	9
	RQh20	20383	IGHV2-69*01_S5121	IGHD3-41*01_S8240	IGHJ5-4*02	CARLHISVWGVIQFSEWSEEDNWFNVW	25	9	IGLV1-ABN*01	IGLJ3-S3*01	CATWDDSLSGVLF	8
			IGHV2-69*01_S5121	IGHD3-41*01_S8240	IGHJ5-4*02	CARLHISVWGVIQFSEWSEEDNWFDVW	25	7	IGLV1-ABN*01	IGLJ3-S3*01	CAAWDDSLSGVLF	2
		20774	IGHV4-NL_22*01_S6977	IGHD3-41*01_S8240	IGHJ6*01	CAREGGSVFGVLIFDEVNYHGLDSW	23	9	IGLV3-LJI31*01	IGLJ1-S1*01	CQVWDSSSEYYIF	8
			IGHV4-NL_22*01_S6977	IGHD3-41*01_S8240	IGHJ6*01	CAREGGSVFGVLIFDEVNYHGLDSW	23	6	IGLV3-LJI31*01	IGLJ1*01	CQVWDDSSEYHIF	11
		20775	IGHV3-128*01	IGHD3-41*01_S8240	IGHJ1*01	CTRVGIKIFGVVLLGEFDRDFFDFW	23	9	IGLV3-LJI31*01_S0001	IGLJ2-S2*01	CQVWDSDSDHVLF	4
		20776	IGHV4-72*01_S2051	IGHD3-41*01_S8240	IGHJ4*01	CARHNRESFGVLQFLEWILKGFDYW	23	6	IGLV3-LJI31*01_S0001	IGLJ2-S2*01	CQVWDNSGDHISF	5
			IGHV4-72*01_S2051	IGHD3-41*01_S8240	IGHJ4*01	CARHIRESFGLSQFLEWVLKGFDYW	23	8	IGLV3-LJI31*01_S0001	IGLJ3-S3*01	CQVWDNSGDHVSF	9
	RRt20	20415	IGHV4-NL_17*01_S0936	IGHD3-41*01_S8240	IGHJ5-1*01	CARDGGITVFGVVDFGEIDENNRFDVW	25	12	IGLV1-ACR*01	IGLJ3-S3*01	CSAWDSSLSTVLF	10
			IGHV4-NL_17*01_S0936	IGHD3-41*01_S8240	IGHJ5-1*01	CAREGGITIFGVVAFGELDEDNRPDVW	25	12	IGLV1-ACR*01	IGLJ3-S3*01	CSAWDSSLSTVLF	7
		20728	IGHV4-NL_36*01_S0936	IGHD3-41*01_S8240	IGHJ6*01	CVRAQTSIYGVVIFGEFDWNGLDSW	23	11	IGLV11-AAY*01	IGLJ3*01	CQVYDSSSNWVF	8
		20740	IGHV3-ABY*01_S4607	IGHD3-41*01	IGHJ5-1*01	CTREGRGLFGLVVFSEWEDNGFDVW	23	12	IGKV1-ABM*01	IGKJ3-I3*01	CQHYYSTPFTF	5
			IGHV3-ABY*01_S4607	IGHD3-41*01	IGHJ5-1*01	CTREGRGLFGLVVFSEWEDNGFDVW	23	12	IGKV1-ABM*01	IGKJ3-I3*01	CQHYYSTPFTF	5
	RTo20	12661	IGHV4-79*02_S9501	IGHD3-41*01_S8240	IGHJ5-4*02	CARGHRITVFGVIIFGEIGGENNWFDVW	26	20	IGKV2-LJI88*01	IGKJ4-I4*01	CMQTLQTPLTF	5
		20364	IGHV4-144*01_S3008	IGHD3-41*01_S8240	IGHJ5-4*02	CSRNGGITIFGVVIFGEFQTNNWFDVW	25	11	IGLV3-LJI32*01	IGLJ2-S2*01	CQVWDISSDRPLF	12
		20373	IGHV4-NL_17*01_S0936	IGHD3-41*01	IGHJ5-1*01	CAKNGGITVFGLVVFGEIDEKNRFDVW	25	9	IGLV1-ACR*01	IGLJ3-S3*01	CSTWDSSLSTVLF	8
			IGHV4-NL_17*01_S0936	IGHD3-41*01	IGHJ5-1*01	CARDGGITIFGLVDFGELDENNRLDVW	25	6	IGLV1-ACR*01	IGLJ3-S3*01	CSAWDSSLSTVLF	1
			IGHV4-NL_17*01_S0936	IGHD3-41*01	IGHJ5-1*01	CARDGGITVFGVVDFGELDENNRFDVW	25	12	IGLV1-ACR*01	IGLJ3-S3*01	CSAWDDGLTTVLF	5
			IGHV4-NL_17*01_S0936	IGHD3-41*01	IGHJ5-1*01	CAREGGITIFGVVAFGELDEDNRLDVW	25	9	IGLV1-ACR*01	IGLJ3-S3*01	CSAWDSSLSIVLF	8
			IGHV4-NL_17*01_S0936	IGHD3-41*01	IGHJ5-1*01	CARDGGITIYGVVDFGELDENNRFDVW	25	8	IGLV1-ACR*01	IGLJ3-S3*01	CSTWDSSLNTLLF	1
		20596	IGHV3-172*01_S2175	IGHD3-41*01	IGHJ5-1*01	CTREIRGIFGLVVFSEWEYYGFDVW	23	7	IGKV1-ABM*01	IGKJ3-I3*01	CQHYYTTPFTF	5
			IGHV3-172*01_S2175	IGHD3-41*01	IGHJ5-1*01	CARKGRGIFGLVTFSEWEYYGFDVW	23	8	IGKV1-ABM*01	IGKJ3-I3*01	CQHYYNTPFTF	6
			IGHV3-ABY*01_S4607	IGHD3-41*01	IGHJ5-1*01	CTREGRGLFGLVIFSEWEDNGFDVW	23	12	IGKV1-ABM*01	IGKJ3-I3*01	CQHYYSTPFTF	5
			IGHV3-172*01_S2175	IGHD3-41*01	IGHJ5-1*01	CTRAGRGIFGLVIFSEWEDIGFDVW	23	7	IGKV1-ABM*01	IGKJ3-I3*01	CQHYYSTPFTF	3
			IGHV3-ABY*01_S4607	IGHD3-41*01	IGHJ5-1*01	CTREGRGLFGLVVFSEWEDNGFDVW	23	12	IGKV1-ABM*01	IGKJ3-I3*01	CQHYYSTPFTF	5
			IGHV3-ABY*01_S4607	IGHD3-41*01	IGHJ5-1*01	CTREGRGLFGLVVFSEWEDNGFDVW	23	12	IGKV1-ABM*01	IGKJ3-I3*01	CQHYYSTPFTF	5
			IGHV3-ABY*01_S4607	IGHD3-41*01	IGHJ5-1*01	CTREGRGLFGLVVFSEWEDNGFDVW	23	12	IGKV1-ABM*01	IGKJ3-I3*01	CQHYYSTPFTF	5
			IGHV3-ABY*01_S4607	IGHD3-41*01	IGHJ5-1*01	CTREGRGLFGLVIFSEWEDNGFDVW	23	11	IGKV1-ABM*01	IGKJ3-I3*01	CQHYYSTPFTF	6
			IGHV3-172*01_S2175	IGHD3-41*01	IGHJ5-1*01	CTREGRRIFGLVIFSEWEFNGFDVW	23	9	IGKV1-ABM*01	IGKJ3-I3*01	CQHYYTTPFTF	5
		20598	IGHV4-AFQ*01_S0868	IGHD3-41*01	IGHJ5-1*01	CARLSTSIFGLVVFDEVEFNRFDVW	23	9	IGKV1-ACI*01	IGKJ3-I3*01	CQHYDSAPFTF	5
		20618	IGHV4-NL_36*01_S0936	IGHD3-41*01_S8240	IGHJ6*01	CARAQISIYGVVIFGEFDYNGLDSW	23	4	IGLV11-AAY*01	IGLJ3*01	CQVYDSSGNWVF	5
			IGHV4-NL_36*01_S0936	IGHD3-41*01_S8240	IGHJ6*01	CARAQISIFGVVIFGEFDWNGLDSW	23	9	IGLV11-AAY*01	IGLJ3*01	CQVYDSSSNWVF	9
	RUi20	20708	IGHV4-117*01_S4901	IGHD3-41*01_S8240	IGHJ6*01	CARDGPITIFGVVIFHEYYNGLDSW	23	11	IGLV3-LJI31*01	IGLJ3*01	CLVWDDTGKYVF	8
		20714	IGHV1-138*01_S3445	IGHD3-41*01_S8240	IGHJ5-1*01	CARGKIAIFGVVIFREYFQRRLDVW	23	16	IGKV2-ACR*01	IGKJ2-I2*01	CMQYLHMYSF	9
		20721	IGHV4-117*01	IGHD3-41*01_S8240	IGHJ5-2*01	CARSGVRGVLQFLEWLDEHNSLDVW	23	0	IGLV3-AED*06	IGLJ1-S1*01	CQVWDSSSDHYIF	5
G2	NA93	20855	IGHV4-117*01_S4879	IGHD3-41*01_S8240	IGHJ5-2*01	CARERVTIFGVVILGEFVGAGLDVW	23	4	IGLV3-S47*01_S0001	IGLJ3*01	CQVWDITTDHWVF	7
			IGHV4-117*01_S4879	IGHD3-41*01_S8240	IGHJ5-2*01	CARERVAIFGVVIFGEFEGAGLDVW	23	8	IGLV3-S47*01_S0001	IGLJ3*01	CLVWDIRSDHWVF	8
			IGHV4-117*01_S4879	IGHD3-41*01_S8240	IGHJ5-2*01	CARERVTIFGVVIFGEFEGAGLDVW	23	12	IGLV3-S47*01_S0001	IGLJ3*01	CLVWDIRSDHWVF	11
			IGHV4-117*01_S4879	IGHD3-41*01_S8240	IGHJ5-2*01	CARERVTIFGVVIFGEFVGAGLDVW	23	6	IGLV3-S47*01_S0001	IGLJ3*01	CQVWDITTDHWVF	6
			IGHV4-117*01_S4879	IGHD3-41*01_S8240	IGHJ5-2*01	CARERVTIFGVVLFGEFVGAGLDVW	23	6	IGLV3-S47*01_S0001	IGLJ3*01	CQVWDITTDHWVF	5
			IGHV4-117*01_S4879	IGHD3-41*01_S8240	IGHJ5-2*01	CARERVKIFGVVIFGEFEGAGLDVW	23	5	IGLV3-S47*01_S0001	IGLJ3*01	CQVWDIRSDHWVF	8
		20856	IGHV4-117*01_S6342	IGHD3-41*01_S8240	IGHJ5-2*01	CARNRITIFGVVIFGEVRVNSLDVW	23	6	IGKV2-LJI57*01	IGKJ3-I3*01	CMQYTHIPFTF	3
		20858	IGHV4-AFQ*01_S0868	IGHD3-41*01_S8240	IGHJ6*01	CARRGRREWGVLQFLEWDPYGLDSW	23	3	IGKV2-AEB*01	IGKJ2-I2*01	CMQGLEFPYSF	3
		20862	IGHV4-144*01_S3113	IGHD3-41*01_S8240	IGHJ5-2*01	CARHAITIFGVVTLGEVFHTSLDVW	23	8	IGLV3-LJI31*01	IGLJ3-S3*01	CQVWDSSDHVLF	12
			IGHV4-144*01_S3113	IGHD3-41*01_S8240	IGHJ5-2*01	CSRYTITIFGVVTLGEVFHNSLDVW	23	3	IGLV3-LJI31*01_S0001	IGLJ2-S2*01	CQVWDSSSDHVLF	10
		20864	IGHV2-69*02_S0779	IGHD3-41*01_S8240	IGHJ4*01	CVRVGIFAWGILQFLEWSTYHFDYW	23	7	IGLV3-S47*01_S0001	IGLJ3*01	CQVWDVSSDHPVF	6
			IGHV2-69*02_S0779	IGHD3-41*01_S8240	IGHJ4*01	CTRVGVLAWGLLQFLEWSTYYFDYW	23	11	IGLV3-S47*01_S0001	IGLJ3*01	CQVWDVSSDHPLF	8
			IGHV2-69*02_S0779	IGHD3-41*01_S8240	IGHJ4*01	CSRVGVFAWGVSQFSEWSPYNFDNW	23	11	IGLV3-S47*01_S0001	IGLJ2-S2*01	CQVWDISSDQPLF	8
			IGHV2-69*02_S0779	IGHD3-41*01_S8240	IGHJ4*01	CTRVGVLAWGLLQFLEWSTYYFDYW	23	10	IGLV3-S47*01_S0001	IGLJ3*01	CQVWDVSSDHPLF	7
		20871	IGHV4-67*01_S8901	IGHD3-41*01_S8240	IGHJ5-1*01	CARRSVTIFGVVILGEFEDNRFDVW	23	7	IGLV3-LJI31*01_S0001	IGLJ1-S1*01	CQVWDSTSDHYIF	8
	NB18	20889	IGHV4-NL_22*01_S6977	IGHD3-41*01	IGHJ6*01	CAREGITLFGLAVFGEYEYNGFDSW	23	12	IGLV6-ADD*01	IGLJ2-S2*01	CQSADGSYNVLF	6
	RCk19	20861	IGHV2-69*01	IGHD3-41*01	IGHJ5-4*01	CARVRFTVFGLVVFGEYSQGRFDVW	23	5	IGLV3-AED*01	IGLJ3-S3*01	CQVWDSSSKYALF	13
			IGHV2-69*01	IGHD3-41*01	IGHJ5-4*01	CARLQFTVFGLVVFGEYSQGRFDVW	23	12	IGLV3-AED*01	IGLJ3-S3*01	CQVWDSSNKYALF	14
	RTo19	20325	IGHV4-AEX*01_S9508	IGHD3-41*01_S8240	IGHJ4*01	CARGSITIFGVVQFQEYEEDAWLDTW	24	14	IGKV4-I3*01	IGKJ2-I2*01	CQQYYSTPYTF	4
			IGHV4-AEX*01_S9508	IGHD3-41*01_S8240	IGHJ4*01	CARGSITIFGVVQLQEYDEDAYFDYW	24	9	IGKV4-I3*01	IGKJ2-I2*01	CQQYYSTPYSF	4
			IGHV4-AEX*01_S9508	IGHD3-41*01_S8240	IGHJ4*01	CARGTITIFGVVQFQEYDEDGWLDYW	24	3	IGKV4-I3*01	IGKJ2-I2*01	CQQSYSTPYSF	5
			IGHV4-AEX*01_S9508	IGHD3-41*01_S8240	IGHJ4*01	CARGSITIFGVVQFQEYEEDAWLDTW	24	13	IGKV4-I3*01	IGKJ2-I2*01	CQQYYSTPYTF	0
		20488	IGHV3-50*01_S1879	IGHD3-41*01	IGHJ6*01	CARSRITVFGLVQFNEDDYGHHGLESW	25	8	IGLV3-LJI31*01_S0001	IGLJ3*01	CQVWDSSSDHWVF	13
			IGHV3-50*01_S1879	IGHD3-41*01	IGHJ6*01	CARNRITIFGLVQFDEDDYGQYGLDSW	25	14	IGLV3-LJI31*01_S0001	IGLJ3*01	CQVWDINSDHWVF	14
			IGHV3-50*01_S1879	IGHD3-41*01	IGHJ6*01	CARNRITVFGLVQFNEDDYGHYGLDSW	25	6	IGLV3-LJI32*01	IGLJ3*01	CQVWNIGSDHWVF	9
			IGHV3-50*01_S1879	IGHD3-41*01	IGHJ6*01	CARNRITIFGLVQFNEDDYGHYGLDSW	25	10	IGLV3-LJI32*01	IGLJ3*01	CQVWDIGSDHWLF	10
			IGHV3-50*01_S1879	IGHD3-41*01	IGHJ6*01	CTRSRITIFGLVQFNEDDYGHYGFDSW	25	8	IGLV3-LJI31*01_S0001	IGLJ3*01	CQVWDSSSDHWLF	12
			IGHV3-50*01_S1879	IGHD3-41*01	IGHJ6*01	CARSRITVFGLVQFDEDDYGHYGLDSW	25	5	IGLV3-LJI32*01	IGLJ3*01	CQVWDSSSDQWVF	13
			IGHV3-50*01_S1879	IGHD3-41*01	IGHJ6*01	CATNRITIFGLVQFDEDDYGQYGLDSW	25	4	IGLV3-LJI31*01_S0001	IGLJ3*01	CQVWDSNSDHWVF	11
			IGHV3-50*01_S1879	IGHD3-41*01	IGHJ6*01	CARSRITIFGLVQFNEDDYGHYGFDSW	25	4	IGLV3-LJI31*01_S0001	IGLJ3*01	CQVWDSSSDHWLF	13
			IGHV3-50*01_S1879	IGHD3-41*01	IGHJ6*01	CARNRITIFGLVQFNEDDYGHYGLDSW	25	5	IGLV3-LJI32*01	IGLJ3*01	CQVWDISSDHWVF	14
			IGHV3-50*01_S1879	IGHD3-41*01	IGHJ6*01	CARNRITIFGLVQFDEDDYGQYGLDSW	25	14	IGLV3-LJI31*01_S0001	IGLJ3*01	CQVWDINSDHWVF	14
		20818	IGHV4-117*01	IGHD3-41*01	IGHJ6*01	CARDGITVFGLIILGEISFYGLDSW	23	8	IGLV2-ABI*01_S9741	IGLJ3*01	CGSYRNGITFIF	6
		20823	IGHV2-173*01	IGHD3-41*01_S8240	IGHJ6*01	CARNRVTIFGVVVLGEYYYYGLDSW	23	2	IGLV3-LJI31*01_S0001	IGLJ6-S6*01	CQVWDRSSDHHDVF	7
			IGHV2-173*01	IGHD3-41*01_S8240	IGHJ6*01	CARNRFTIFGLVIMGEYYYYGLDSW	23	9	IGLV3-LJI31*01_S0001	IGLJ6-S6*01	CQVWDSSSDHHDVF	11
			IGHV2-173*01	IGHD3-41*01_S8240	IGHJ6*01	CARNRITIFGVVILGEYYYYGLDSW	23	5	IGLV3-LJI31*01_S0001	IGLJ6-S6*01	CQVWDRSSDHHDVF	8
			IGHV2-173*01	IGHD3-41*01_S8240	IGHJ6*01	CARNRVTIFGVVVLGEYYYYGLDSW	23	2	IGLV3-LJI31*01_S0001	IGLJ6-S6*01	CQVWDRSSDHHDVF	7
		20825	IGHV4-93*01	IGHD3-41*01	IGHJ6*01	CAREDFTIFGLVTYKEGYYSGLDSW	23	10	IGLV3-AAV*01	IGLJ3*01	CYSGDDNSRVF	5
			IGHV4-93*01	IGHD3-41*01	IGHJ6*01	CAREGITIFGLVTFKEYYYHGLDSW	23	6	IGKV1-ABM*01	IGLJ3*01	CYSGDDNSRVF	5
		20826	IGHV4-144*01_S3113	IGHD3-41*01	IGHJ6*01	CARDHLTIFGLVIMNELDYNGLDSW	23	10	IGLV3-S47*01_S0001	IGLJ1-S1*01	CQVWDINGDYIF	2
			IGHV4-144*01_S3113	IGHD3-41*01	IGHJ6*01	CARDHFTVFGLVSLHELDYYGLDSW	23	5	IGLV3-S47*01_S0001	IGLJ1-S1*01	CQVWDVTNNYIF	10
			IGHV4-144*01_S3113	IGHD3-41*01	IGHJ6*01	CARDHLSMFGLAVFHESDYYGLDSW	23	7	IGLV3-S47*01_S0001	IGLJ1-S1*01	CQVWGISGDYIF	6
			IGHV4-144*01_S3113	IGHD3-41*01	IGHJ6*01	CARDHLSMFGLAVFHESDYYGLDSW	23	6	IGLV3-S47*01_S0001	IGLJ1-S1*01	CQVWGISGDYIF	3
			IGHV4-144*01_S3113	IGHD3-41*01	IGHJ6*01	CARDHITVFGLVIMNEFDYYGLDSW	23	11	IGLV3-S47*01_S0001	IGLJ1-S1*01	CQVWDISNDYIF	5
			IGHV4-144*01_S3113	IGHD3-41*01	IGHJ6*01	CARDHMTIFGLVIMNELDYYGLDSW	23	11	IGLV3-S47*01_S0001	IGLJ1-S1*01	CQVWDISGDYIF	2
			IGHV4-144*01_S3113	IGHD3-41*01	IGHJ6*01	CARDHMTLFGLLIMNELDYYGLDSW	23	14	IGLV3-S47*01_S0001	IGLJ1-S1*01	CQVWDISGDYIF	4
		20830	IGHV4-NL_40*01_S0696	IGHD3-41*01	IGHJ6*01	CARDRYTIFGLVVMNEVQSYGLDSW	23	8	IGLV3-AAV*01	IGLJ3*01	CYSGDGNSWVF	5
		20832	IGHV2-69*02_S0779	IGHD3-41*01	IGHJ4*01	CARVSFTIFGLVIMGEFNGPWFDFW	23	9	IGLV3-AEB*02	IGLJ1*01	CHSTDSSGNHSIF	6
		20833	IGHV4-149*01_S0794	IGHD3-41*01_S8240	IGHJ4*01	CARLISLTIFGVVALGETETEFDYW	23	15	IGKV7-ACH*01	IGKJ1-I1*01	CLQTKNSPRTF	6
		20837	IGHV2-ABU*01_S6524	IGHD3-41*01	IGHJ5-1*01	CAREVVTIFGLVYFREYEDNRFDVW	23	12	IGLV3-ADT*02	IGLJ3*01	CASWDNSDTHLIF	7
		20840	IGHV4-174*02	IGHD3-41*01	IGHJ5-1*01	CVRLGGTIFGLVIFGELDWDRFDVW	23	9	IGLV3-LJI32*01	IGLJ6-S6*01	CQVWDNSSDHPVF	15
			IGHV4-174*02	IGHD3-41*01	IGHJ5-1*01	CVRLGGTIFGLVIFGELDWDRFDVW	23	7	IGLV3-LJI32*01	IGLJ6-S6*01	CQVWDNSSDHPVF	9
			IGHV4-174*02	IGHD3-41*01	IGHJ5-1*01	CAKLGGTVFGLVVFGEVEWDRFDVW	23	7	IGKV1-AAY*01	IGKJ4-I4*01	CQQYDTDPLTF	5
			IGHV4-174*02	IGHD3-41*01	IGHJ5-1*01	CAKLGGRIFGVVIFGELEWDRFDVW	23	5	IGLV6-ADW*01	IGLJ2-S2*01	CKSYDSTAYVLF	11
			IGHV4-174*02	IGHD3-41*01	IGHJ5-1*01	CVRLGGTVFGLAIFGELDWDRFDVW	23	6	IGLV3-AED*06_S0001	IGLJ6-S6*01	CQVWDNSSDHPVF	15
			IGHV4-174*02	IGHD3-41*01	IGHJ5-1*01	CAKLGGRIFGVVIFGELEWDRFDVW	23	5	IGLV3-LJI32*01	IGLJ6-S6*01	CQVWDSSTDHPAF	7
			IGHV4-174*02	IGHD3-41*01	IGHJ5-1*01	CARLGGRIFGVVIFGELEWDRFDVW	23	5	IGLV3-LJI32*01	IGLJ6-S6*01	CQVWDSSTDHPVF	6
			IGHV4-174*02	IGHD3-41*01	IGHJ5-1*01	CAKLGGRIFGVVIFGELEWDRFDVW	23	5	IGLV3-LJI32*01	IGLJ6-S6*01	CQVWDSSTDQPAF	11
			IGHV4-174*02	IGHD3-41*01	IGHJ5-1*01	CAKLGGTVFGLVIFGEVEWDRFDVW	23	9	IGKV1-AAY*01	IGKJ4-I4*01	CQQYDNDPLTF	3
		20843	IGHV4-93*01	IGHD3-41*01	IGHJ2*01_S5087	CAREGGAVFGLVIFGEVDYWFFDIW	23	7	IGLV3-AAV*01	IGLJ3*01	CYSADGNSRVF	5
			IGHV4-93*01	IGHD3-41*01	IGHJ2*01_S5087	CAREGGAVFGLVIFGEVDYWFFDIW	23	7	IGLV3-AAV*01	IGLJ3*01	CYSADGNSRVF	5
			IGHV4-93*01	IGHD3-41*01	IGHJ2*01_S5087	CAREGGVIFGLVIFGEVDYLYFDIW	23	10	IGLV3-AAV*01	IGLJ3*01	CCSGDDNSRVF	5
			IGHV4-93*01	IGHD3-41*01	IGHJ2*01_S5087	CAREGGAIFGLVIFGEVDYLYFDIW	23	8	IGLV3-AAV*01	IGLJ3*01	CCSGDDNSRVF	3
			IGHV4-93*01	IGHD3-41*01	IGHJ2*01_S5087	CAREGGAIFGLVVLGEIDYGFFDIW	23	11	IGLV3-AAV*01	IGLJ3*01	CFSGDDNRRVF	2
			IGHV4-93*01	IGHD3-41*01	IGHJ2*01_S5087	CAREGGGIFGLVIFGEVDYLYFDIW	23	8	IGLV3-AAV*01	IGLJ3*01	CCSGDDNSRVF	7
			IGHV4-93*01	IGHD3-41*01	IGHJ2*01_S5087	CARETGAVFGLVIMGEIDYLYLDIW	23	11	IGLV3-AAV*01	IGLJ3*01	CYSGDDNSRVF	4
			IGHV4-93*01	IGHD3-41*01	IGHJ2*01_S5087	CAREYGAIFGLVILGEVDYWYFDIW	23	1	IGLV3-AAV*01	IGLJ3*01	CSSGDDNSRVF	2
	RWc20	12681	IGHV4-150*01_S5542	IGHD3-41*01	IGHJ5-2*01	CVRDNRITIFGLVIFKEIEPSGNSLDVW	26	2	IGLV6-AAC*01	IGLJ3-S3*01	CQSADDSYTVLF	7
			IGHV4-150*01_S5542	IGHD3-41*01	IGHJ5-2*01	CVRDNRITVFGLVIFKEIEPSGNSLDVW	26	6	IGLV6-AAC*01	IGLJ3-S3*01	CQSADDNYTVLF	5
			IGHV4-150*01_S5542	IGHD3-41*01	IGHJ5-2*01	CVRDNRITIFGLVIFKEIEPSGNSLDVW	26	2	IGLV6-AAC*01	IGLJ3-S3*01	CQSADDSYTVLF	7
			IGHV4-150*01_S5542	IGHD3-41*01	IGHJ5-2*01	CVRDNRITIFGLVIFKEIEPSGNSLDVW	26	2	IGLV6-AAC*01	IGLJ3-S3*01	CQSADDSYTVLF	7
		18861	IGHV3-NL_17*01_S8244	IGHD3-41*01	IGHJ5-4*01	CTTGPRLTIFGLVIFREYDRFDVW	22	11	IGKV2-AEB*01_S5603	IGKJ1-I1*01	CMQTLEFPWTF	6
			IGHV3-NL_17*01_S8244	IGHD3-41*01	IGHJ5-4*01	CTTGPRLTTFGLVLFREYDRFDVW	22	4	IGKV2-AEB*01_S5603	IGKJ1-I1*01	CMQTLEFPWTF	2
			IGHV3-NL_17*01_S8244	IGHD3-41*01	IGHJ5-4*01	CTTGPRLTIFGLVVFREYDRFDVW	22	10	IGKV2-AEB*01_S5603	IGKJ1-I1*01	CMQTLEFPWTF	5
		20873	IGHV4-NL_33*01_S5330	IGHD3-41*01	IGHJ5-4*01	CARERITIFGLVIFGEGHQNRFDVW	23	16	IGLV3-LJI31*01_S0001	IGLJ1-S1*01	CQVWDSNSDHYIF	6
G3	RCj20	20808	IGHV4-93*01	IGHD3-41*01	IGHJ6*01	CAREVLTIFGLASFQEWDYYGLDSW	23	8	IGLV3-LJI31*01_S0001	IGLJ2-S2*01	CQVWDSSSDYLF	5
	RGi20	20797	IGHV4-NL_31*01_S7693	IGHD3-41*01_S8240	IGHJ6*01	CAREGTIIFGVVILGESNYSGLDSW	23	11	IGLV1-ADA*01	IGLJ3*01	CEAWDDSLNSRMF	4
			IGHV4-NL_31*01_S7693	IGHD3-41*01_S8240	IGHJ6*01	CAREGTIIFGVVILGESNYSGLDSW	23	12	IGLV1-ADA*01	IGLJ3*01	CEAWDDSLNSRMF	4
	RPf20	18747	IGHV3-AET*01_S5460	IGHD3-41*01_S8240	IGHJ4*01	CIRTGGIFGVIILREVDYGALDYW	22	4	IGLV3-AAV*02	IGLJ1-S1*01	CYSGDDNTYIF	8
G4	RTm20	20301	IGHV4-NL_27*01_S1458	IGHD3-41*01_S8240	IGHJ4*01	CARGATTIFGVVIFGETTFVGYFDYW	24	11	IGKV2-AEB*01	IGKJ2-I2*01	CMQALEFPYSF	4
		20302	IGHV4-150*01_S8038	IGHD3-41*01_S8240	IGHJ5-4*02	CARDGVEVWGLVQFLEWSRRYWFDVW	24	10	IGLV3-AEC*01	IGLJ3-S3*01	CQSADSSAHHVLF	5
		20784	IGHV3-122*01	IGHD3-41*01_S8240	IGHJ6*01	CAREGITLFGVAALEEYYYHGLDSW	23	4	IGLV3-AAV*02	IGLJ3*01	CSSGDDNGWVF	7
		20785	IGHV4-117*01	IGHD3-41*01_S8240	IGHJ6*01	CARDRVTIFGVVKFNELTYDGLDSW	23	7	IGKV2-ADV*01	IGKJ1-I1*01	CMQTLQTPWTF	7
G5	RBu20	20495	IGHV2-69*02_S0779	IGHD3-41*01_S8240	IGHJ4*01	CARVLGITIFGVVIFGEYSSTPYFDYW	25	12	IGKV1-ACE*01	IGKJ4-I4*01	CQQHNSHPPTF	7
		20898	IGHV3-187*02_S8458	IGHD3-41*01_S8240	IGHJ5-1*01	CTPRNFTIFGVVLFKEVGYNRFDVW	23	7	IGLV3-LJI31*01_S0001	IGLJ3-S3*01	CQVWDSSSDHVLF	7
	RHq19	20342	IGHV3-88*01	IGHD3-41*01	IGHJ5-4*02	CARNSITIFGLVKFRELESENWFDVW	24	12	IGKV1-ABM*01_S8638	IGKJ3-I3*01	CQHYYSIPFTF	1
		20896	IGHV3-187*02	IGHD3-41*01_S8240	IGHJ4*01	CTRDTSAIWGLLQFSESTTISHDLW	23	6	IGLV3-AAV*01	IGLJ2-S2*01	CYSGDGNSGLF	7
			IGHV3-187*02_S8458	IGHD3-41*01_S8240	IGHJ4*01	CTRESSAIWGLLQFSESTIISHDLW	23	14	IGLV3-AAV*01	IGLJ2-S2*01	CYSGDGNSGLF	8
			IGHV3-187*02	IGHD3-41*01_S8240	IGHJ4*01	CTRESSAIWGLSQFLESTTISHDLW	23	4	IGLV3-AAV*01	IGLJ2-S2*01	CYSGDGNSGLF	4
	RLt18	18872	IGHV4-79*01	IGHD3-41*01_S8240	IGHJ5-2*01	CAREGITVFGVVMFGETHKSLDVW	22	9	IGLV3-AEC*01	IGLJ1*01	CQSADSSGNFIF	6
		20337	IGHV3-73*02_S0085	IGHD3-41*01	IGHJ6*01	CTREGSTIFGLVIFGEVSSDYHLDSW	24	15	IGLV8-ABK*01	IGLJ1*01	CLLYMDSDIYIF	10
		20886	IGHV3-53*01	IGHD3-41*01_S8240	IGHJ6*01	CAREGVTIFGVVMLGEVARDGLDSW	23	3	IGLV3-LJI31*01_S0001	IGLJ3-S3*01	CQVWDSSSDHVLF	14
		20888	IGHV4-67*01_S0439	IGHD3-41*01_S8240	IGHJ6*01	CARSLLTIFGVVTLGEYTWDFFDSW	23	9	IGLV2-ABI*01_S7244	IGLJ1*01	CGSYRGGSTYIF	8
G6	RCf19	20361	IGHV3-50*01_S1879	IGHD3-41*01	IGHJ6*01	CARGGITIFGLVKFSEVEDNYGLDSW	24	12	IGLV2-ABE*01_S2946	IGLJ1*01	CGSYAGSSTYIF	9
	RFu20	12683	IGHV4-NL_21*01_S1118	IGHD3-41*01_S8240	IGHJ5-1*01	CARNQERITIFGVVIFGEVATTNRFDVW	26	9	IGLV3-S47*01_S0001	IGLJ2-S2*01	CQVWDITTDHGLF	5
		18893	IGHV3-NL_17*01_S8244	IGHD3-41*01	IGHJ4*01	CTKENITVFGLVVFREYEEHLDYW	22	9	IGLV3-AAV*01	IGLJ3*01	CYSGDGNSRVF	7

Sequences highlighted in blue were produced as antibodies and tested for binding. Sequences highlighted in brown were additionally tested for neutralization.

Table S3. ***IGHD3-41*** **genotypes**

Group	Animal ID	IGHD3-41 Genotype
G1	RHu20	*01/*01
	RQh20	S8240/S8240
	RQR20	S8240/S8240
	RRt20	S8240/*01
	RTo20	S8240/*01
	RUl20	S8240/S8240
G2	NA93	S8240/S8240
	NB18	*01/*01
	NF14	S8240/*01
	RCk19	S8240/*01
	RTo19	S8240/*01
	RWc20	*01/*01
G3	RCj20	S8240/*01
	RFk20	S8240/*01
	RGi20	S8240/S8240
	RKn20	S8240/S8240
	RPf20	S8240/S8240
	RSo20	S8240/S8240
G4	RIe20	S8240/*01
	RQp20	*01/*01
	RTm20	S8240/S8240
	RVt20	S8240/S8240
	RVu20	S8240/*01
	RZu20	S8240/*01
G5	RBu20	S8240/S8240
	RHq19	S8240/*01
	RLt18	S8240/*01
	RTk18	S8240/*01
	RVo19	*01/*01
	RWh19	S8240/*01
G6	MR83	S8240/*01
	MT09	S8240/*01
	RCf19	S8240/*01
	RFu20	S8240/*01
	RLN20	S8240/*01
	RYm20	S8240/S8240

*01, frame 3: ITIFGLVII; frame 1: VLQYLDWLLY
*01_S8240, frame 3: ITIFGVVIT; frame 1: VLQFLEWLLH
